# Heterodinuclear Co(III)Na(I) Catalysts for the Ring-Opening Copolymerization of Propene Oxide and Carbon Dioxide

**DOI:** 10.1021/acs.macromol.5c01529

**Published:** 2025-07-04

**Authors:** Frederica Butler, Francesca Fiorentini, Katharina H. S. Eisenhardt, Charlotte K. Williams

**Affiliations:** Department of Chemistry, 6396 University of Oxford, Chemistry Research Laboratory, Oxford OX1 3TA, United Kingdom

## Abstract

The ring-opening copolymerization of propene oxide and carbon dioxide is an effective route to make polycarbonates. Catalysts showing high activities, high polymer selectivities, molecular weight control, and tolerance to impurities are rare. Here, a series of four Co­(III)­Na­(I) catalysts are tested for the ring-opening copolymerization of propene oxide and carbon dioxide. The complexes have systematic variations in the ligand structure in which both Co­(III) and Na­(I) binding sites are modified. Significant differences in catalyst performance are observed, with the lead catalyst featuring six oxygen donors and an ethylene diamine linker. This catalyst shows excellent activity and high poly­(propene carbonate) selectivity at elevated temperatures (TOF = 1428 h^–1^, poly­(propene carbonate) selectivity = 98%, 20 bar CO_2_, 70 °C), outperforming analogous Co­(III)­K­(I) catalysts. These results inform on the optimal coordination environment and operating conditions for the Co­(III)­Na­(I) catalysts and highlight the benefits of using Na­(I) relative to heavier s-block metals in the heterodinuclear catalysts.

## Graphical abstract

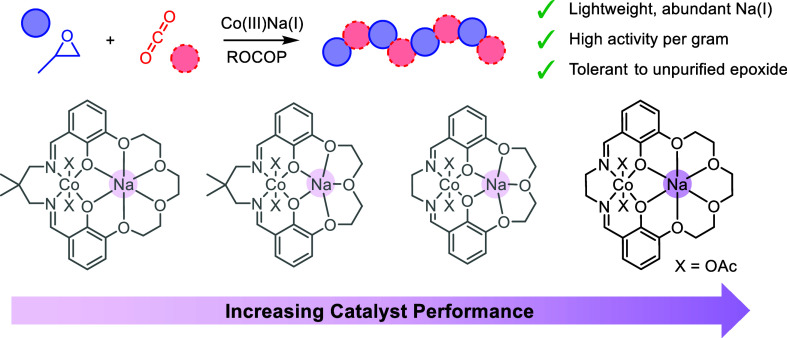

## Introduction

As a common waste product of many industrial processes, carbon dioxide (CO_2_) is an abundant, renewable, and inexpensive feedstock for future chemical and materials synthesis.^1–8^ The catalytic activation of CO_2_ to make polymers is achieved by its ring-opening copolymerization (ROCOP) with epoxides to produce different classes of polycarbonate.^9–13^ The ROCOP of CO_2_ and propene oxide (PO) is particularly desirable as the resulting polycarbonates show mid/low glass transition temperatures (35 °C < *T*_g_ < 42 °C) and hydroxyl-end capped polyols are suited to make flexible foams, elastomers, and surfactants.^14–19^ Additionally, PO is readily available, produced at >10 million tonnes scale worldwide in 2023, and it is widely used to make polyether polyols.^20^

The development of better performing propene oxide/CO_2_ ROCOP catalysts is essential both to accelerate industrialization of these materials and help improve material properties.^9,13^ Desirable catalyst features include high activity (both per mole and per gram of catalyst), high selectivity for polymer, and straightforward synthesis from abundant precursors. Tolerance toward impurities present in commercial, unpurified epoxides such as water and diols is also important as monomer purification is time-consuming and costly. Catalysts exhibiting all these features remain difficult to design, in particular for PO/CO_2_ ROCOP, in which reduced ring strain and the lower barrier to formation of a cyclic side product (relative to more strained epoxides) make high rates and selectivities difficult to achieve.^11,21,22^ Generally, higher reaction temperatures increase catalytic activity and overall conversion but often reduce selectivity as formation of the cyclic carbonate side product is thermodynamically favored.^9,11,23^

Complexes of cobalt­(III), in particular Co­(III) salen complexes incorporating ionic cocatalysts, remain some of the highest performing catalysts for this process.^24–36^ The leading catalysts, reported by Lee and co-workers, feature ligands with four covalently attached cocatalysts; these catalysts achieve outstanding activity and selectivity values.^26^ There are still aspects to improve in these catalysts including the need for stoichiometric (vs catalyst) ionic cocatalysts, such as PPNCl (PPN = bis­(triphenylphosphine­(iminium)), which are toxic and corrosive, and the multistep catalyst syntheses, which result in low overall yields and complicate larger-scale applications.

Heterodinuclear metal complexes show promise as highly active and selective epoxide/CO_2_ ROCOP catalysts and can benefit from performance enhancements attributed to intermetallic synergy.^11,37^ These heterodinuclear catalysts can be simple to synthesize, operate without a cocatalyst, and employ ligands that are easily modifiable, allowing for systematic structure–activity investigations.^38–42^

In 2020, our group reported the first heterodinuclear catalysts for PO/CO_2_ ROCOP, combining Co­(III) and M­(I) (where M­(I) = group 1 metal) coordinated by a diphenolate-di-imine-tetra-ether macrocyclic ligand.^43^ Later, the investigation of a series of Co­(III)­M­(I/II) (where M­(I/II) = K­(I), Na­(I), Ca­(II), Sr­(II), and Ba­(II)) catalysts for PO/CO_2_ ROCOP, under the same conditions (20 bar of CO_2_ pressure, 50 °C), revealed that the Co­(III)­K­(I) complex showed the highest performance. The Co­(III)­K­(I) catalyst showed a turnover frequency (TOF) = 528 h^–1^, poly­(propene carbonate) selectivity = 98%, at 20 bar CO_2_ and 50 °C. The next-best catalyst was the Co­(III)­Na­(I) complex, followed by the Co­(III)­M­(II) catalysts.^38^ A generalizable linear free energy relationship was established between s-block metal Lewis acidity, as quantified by the s-block metal (aqua) complex p*K*_a_, and both the catalytic activity and selectivity.^38^ Further changes to the ancillary ligand structure resulted in a Co­(III)­K­(I) catalyst, coordinated by a diphenolate-di-imine-tetra-ether acyclic ligand, which showed both higher activity and selectivity, with TOF = 1728 h^–1^, poly­(propene carbonate) selectivity = 92%, at 20 bar CO_2_ and 80 °C.^44^

Mechanistic investigations using the Co­(III)­K­(I) catalyst for PO/CO_2_ ROCOP indicated a rate law that is first order in both epoxide and catalyst concentration, supporting a mechanism in which epoxide ring opening is the rate-determining step.^39,43,44^ Density functional theory (DFT) calculations, supported by Eyring analyses, indicated that the rate-determining step involves Co­(III)-PO adduct formation and an attack from the K­(I)-carbonate chain end.^23^ The combination of experimental and theoretical investigations supports a dinuclear metalate mechanism in which a Co­(III)-epoxide adduct is ring-opened by a K­(I)-bound carbonate intermediate to give an alkoxide intermediate. CO_2_ then inserts, rapidly and reversibly, into the alkoxide intermediate to regenerate the carbonate intermediate.^23,44^ An analogous mechanism, including the differentiated roles for the two metals, is expected for the Co­(III)­Na­(I) catalysts (Figure 1).

**Figure 1:**
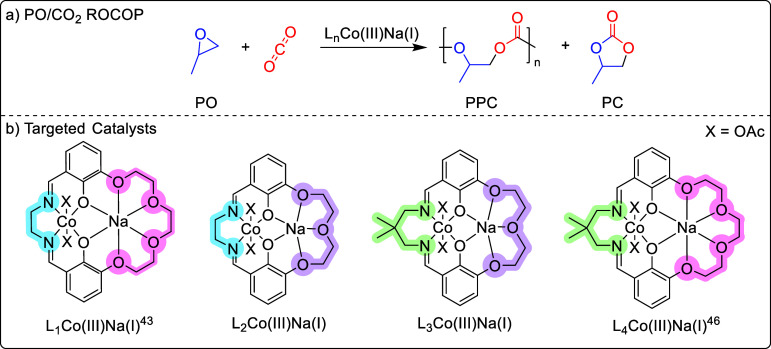
(a) Scheme for PO/CO_2_ ROCOP. (b) Targeted catalysts for PO/CO_2_ ROCOP L_*n*_Co­(III)­Na­(I) (*n* = 1–4). L_1_Co­(III)­Na­(I) and L_4_Co­(III)­Na­(I) are previously reported.^43,46^

So far, almost all heterodinuclear catalyst development for PO/CO_2_ ROCOP has focused on Co­(III)­K­(I) complexes, with the promising Co­(III)­Na­(I) complexes tested only under specific conditions (50 °C, 20 bar of CO_2_) and not subjected to detailed investigation. There are many benefits to using sodium in catalysis: sodium is significantly more abundant on Earth compared to potassium (sea abundance = 1.08 × 10^4^ mgL^–1^ for Na vs 3.99 × 10^2^ mg L^–1^ for K) and is lighter (relative atomic mass = 23.0 g mol^–1^ for Na vs 39.1 g mol^–1^ for K).^45^ Hence, catalysts featuring Co­(III)­Na­(I) should show high activities per gram and warrant a more detailed investigation and understanding.

Here, four Co­(III)­Na­(I) catalysts are tested for the ROCOP of PO and CO_2_, under varying conditions, to identify the optimal ligand coordination environment and operating conditions for the Co­(III)­Na­(I) metal combination (Figure 1). Two of the catalysts, L_2_Co­(III)­Na­(I) and L_3_Co­(III)­Na­(I), are new complexes, and two complexes, L_1_Co­(III)­Na­(I) and L_4_Co­(III)­Na­(I), are previously reported, but here, catalyst performance is investigated under three different sets of conditions that are chosen with regard to future application and industrial relevance.^23,43^ Across the series, systematic modifications are made to the ligands, in particular to the di-imine linker (ethyl and dimethyl propyl linkers) and the number of oxygen donors (five and six) in the ether sites. These ligand structural variations are selected since prior DFT calculations suggest that they exert influence over the metal sites during the rate-determining and selectivity-determining steps.^23^

## Results

### Synthesis and Characterization of L_*n*_Co­(III)­Na­(I) Catalysts (*n* = 1–4)

Complexes L_1_Co­(III)­Na­(I) and L_4_Co­(III)­Na­(I) were synthesized according to literature procedures.^43,46^ Successful synthesis and purity of the desired complexes were confirmed via NMR spectroscopy and elemental analysis (Figures S1–S5 and S22–S26, see the Supporting Information for experimental details). L_2_Co­(III)­Na­(I) and L_3_Co­(III)­Na­(I) are novel catalysts and were each synthesized using a template cyclization methodology. In each case, a methanol solution of the relevant diamine was added dropwise, over the course of 3 h, to a solution of the dialdehyde proligand and NaOAc, under reflux, also dissolved in methanol. The solution was allowed to cool to room temperature, before an equivalent of Co­(OAc)_2_ was added and the solution stirred for 16 h under nitrogen. Then, one equivalent of acetic acid was added and the solution stirred in air for a further 72 h (Scheme 1, see the Supporting Information for experimental details).

**Scheme 1:**
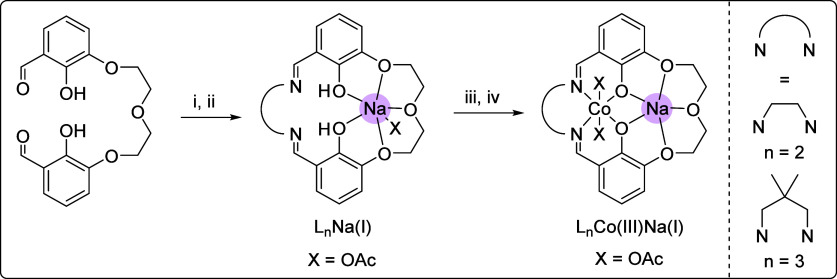
Synthesis of L_*n*_Co­(III)­Na­(I) (*n* = 2, 3)^a^

a (i) Na­(OAc), MeOH, reflux, N_2_; (ii) ethylene diamine (*n* = 2) or 2,2-dimethylpropane-1,3-diamine (*n* = 3), MeOH, reflux, N_2_, 3 h; (iii) Co­(OAc)_2_, MeOH, rt, N_2_, 16 h; (iv) acetic acid, MeOH, rt, air, 72 h.

L_3_Co­(III)­Na­(I) was isolated in good yield (62%), and successful synthesis was confirmed using NMR spectroscopy (Figures S14–S19 and S21). L_3_Co­(III)­Na­(I) was further characterized by MALDI-TOF mass spectrometry, and purity was confirmed by elemental analysis (Figure S20). Single crystals, suitable for X-ray diffraction experiments, were grown via slow evaporation of hexane into a solution of the complex in chloroform at room temperature (Figures S38 and S39).

Although synthesized analogously to L_3_Co­(III)­Na­(I), L_2_Co­(III)­Na­(I) exhibits a more complex speciation in solution compared to the other Co­(III)­Na­(I) complexes. The ^1^H NMR spectrum of L_2_Co­(III)­Na­(I), in CDCl_3_, shows resonances that are broad, whereas its spectrum in MeCN-d_3_ shows clear and resolvable peaks (Figure 2 and Figures S6–S10). The ^1^H NMR spectrum, in MeCN-d_3_, shows a greater number of resonances than would be expected from the symmetry of these complexes, prompting further investigation (Figures S6–S10). DOSY NMR spectroscopy revealed the presence of two species in solution, with distinct diffusion coefficients (10^–9.04^ and 10^–8.93^ m^2^s^–1^) (Figure 2 and Figure S11).^43^ The presence of two species is further supported by MALDI-TOF spectrometry in which two distinct molecular weight distributions are observed (at *m*/*z* = 450.08 and *m*/*z* = 876.98). The *m*/*z* values of the two species are consistent with removal of the acetate coligands and generation of the charged Co­(II) analogues of the proposed solution-phase structures *in situ* during the measurement (Figure S12). The detection of cationic Co­(II) species from starting Co­(III) compounds during the MALDI-TOF spectrometry experiments may assist volatilization and is consistent with prior reports on related compounds.^40,43^ Analysis of the structure, obtained by single-crystal X-ray diffraction experiments on L_2_Co­(III)­Na­(I), reveals that the Na­(I) site lies significantly above the ligand plane (defined by the ether O atoms, with the distance being ∼1.179 Å, O1–O5, Figure 6 and Figures S36 and S37). This solid-state speciation could indicate a weaker coordination of Na­(I) within this ligand framework, perhaps resulting in different complexes or structures in solution. Combining the above spectroscopic data, L_2_Co­(III)­Na­(I) is proposed to exist in MeCN-d_3_ as a mixture of the targeted Co­(III)­Na­(I) complex and a Na­(I)-bridged aggregate (Figure 2).

**Figure 2:**
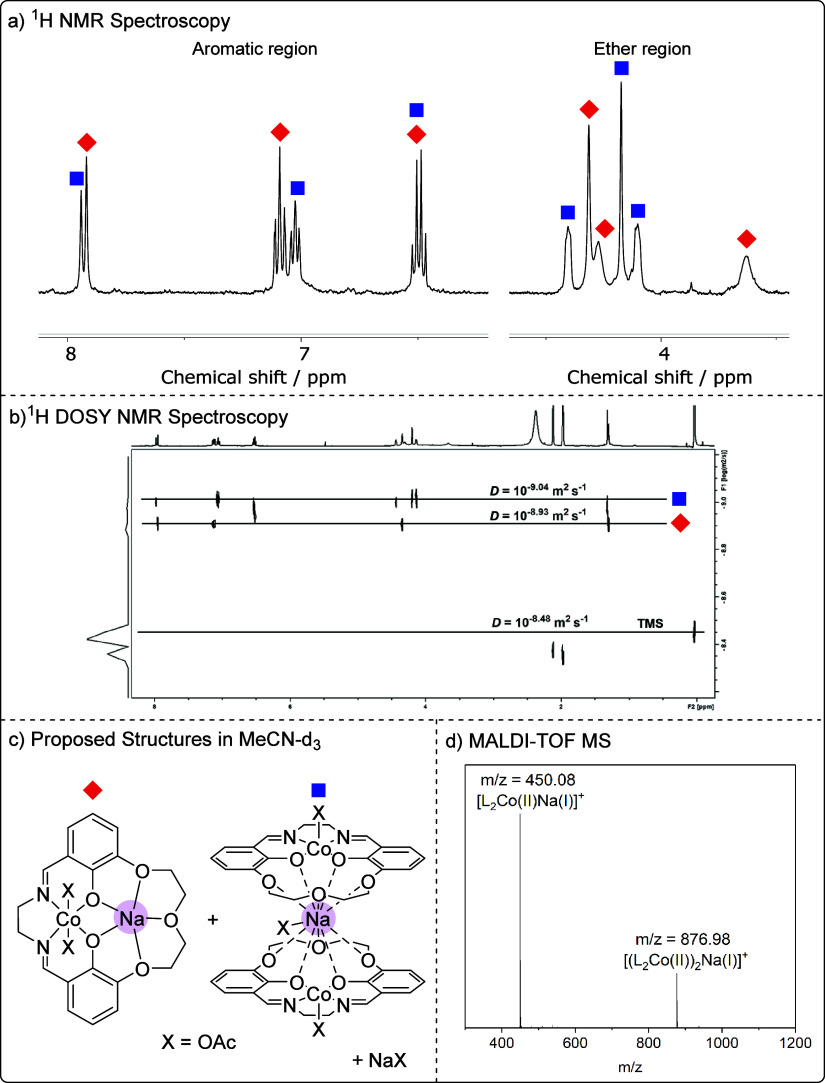
Characterization data for L_2_Co­(III)­Na­(I). (a) ^1^H NMR spectrum (500 MHz, MeCN-d_3_). (b) ^1^H DOSY NMR spectrum (500 MHz, MeCN-d_3_). (c) Proposed structures present in MeCN-d_3_. (d) MALDI-TOF mass spectrum, showing the presence of two species (Figure S12).

**Figure 3:**
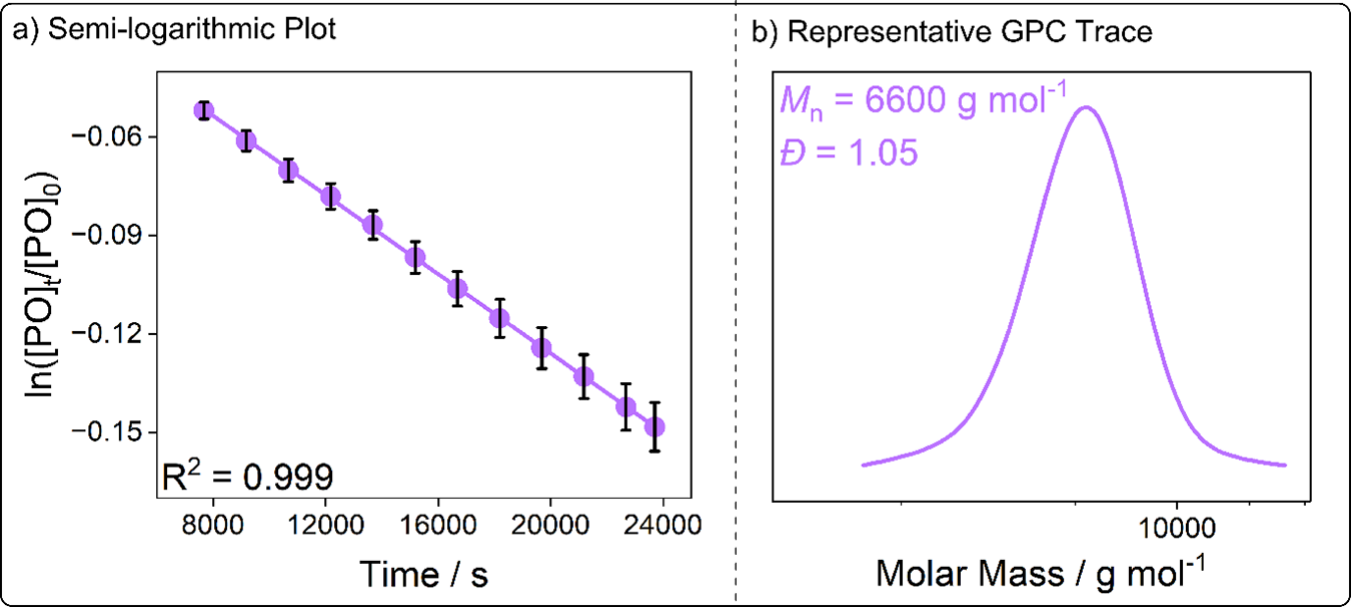
(a) Representative semi-logarithmic plot of ln­([PO]_*t*_/[PO]_0_) vs time, where *k*_obs_ is calculated from the gradient. (b) Representative PPC GPC trace.

**Figure 4:**
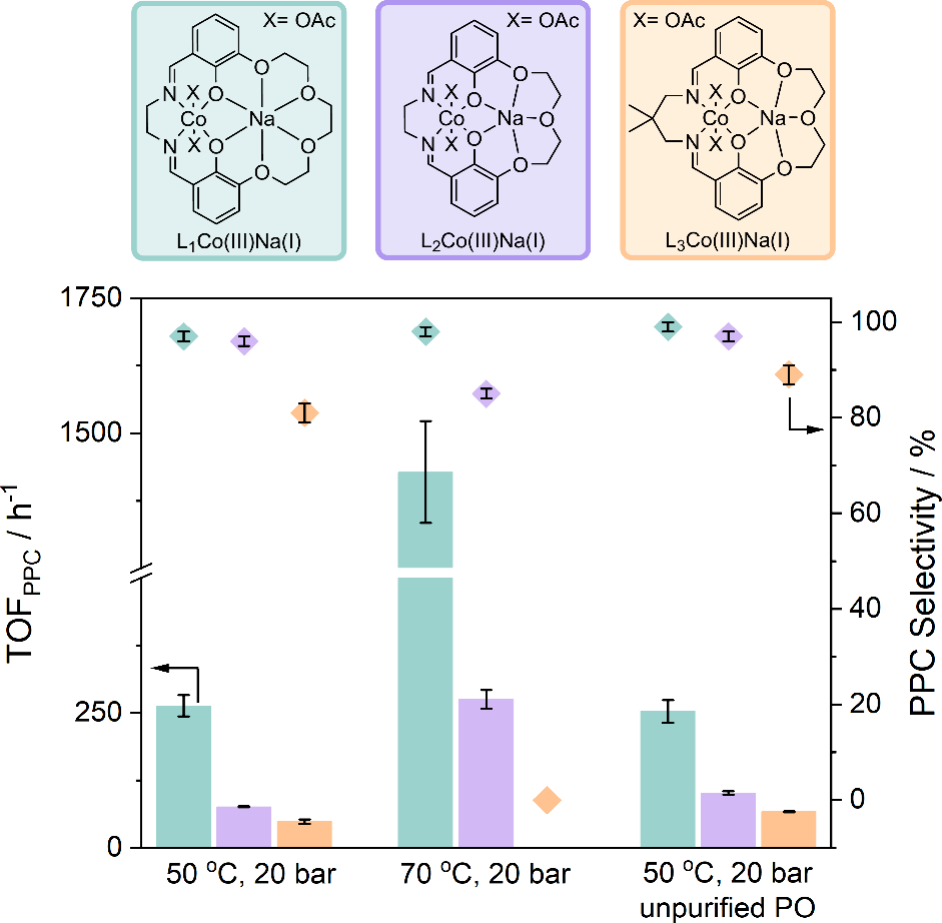
Summary of activity (*k*_obs_) and polycarbonate selectivity data for catalysts L_*n*_Co­(III)­Na­(I) (*n* = 1–3) for the ROCOP of PO and CO_2_ under different conditions.

**Figure 5:**
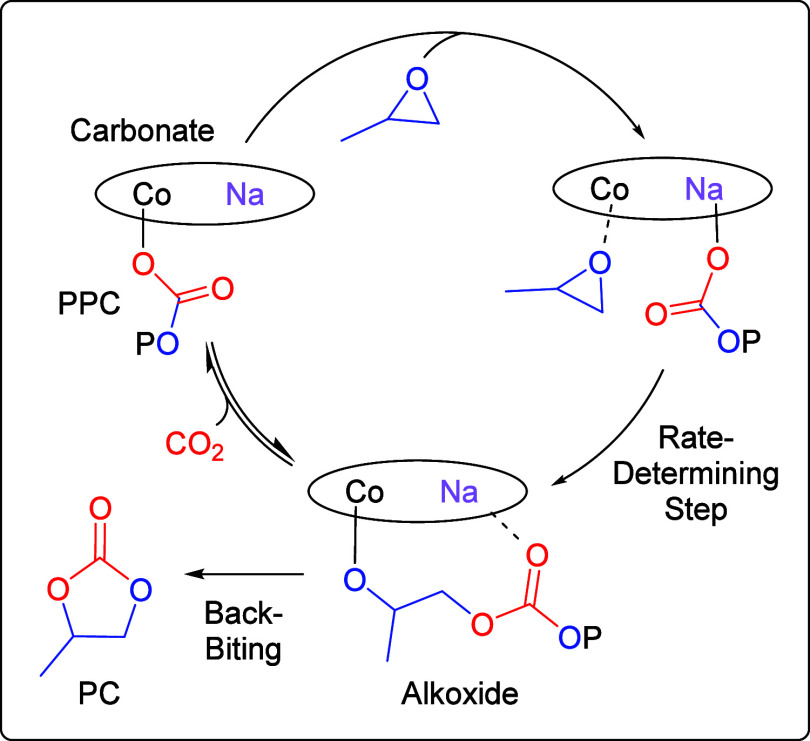
Proposed mechanism based on DFT and experimental investigations for the related Co­(III)­K­(I) catalyst (*P* = growing polymer chain).^23,43^

Variable temperature (VT) ^1^H NMR spectroscopy revealed no notable change in the spectrum (from −40 to 75 °C), implying that the two species are not exchanging over this temperature range (Figure S13). From the integration of the distinct imine peaks in the ^1^H NMR spectrum, the ratio of L_2_Co­(III)­Na­(I) to the Na­(I)-bridged aggregate is estimated as 2:1 in MeCN-d_3_.

### Propene Oxide and Carbon Dioxide ROCOP Catalysis

The series of catalysts L_*n*_Co­(III)­Na­(I) (*n* = 1–4) was tested for the ring-opening copolymerization of PO and CO_2_ under the following conditions: neat PO, 1:20:4000 [catalyst]_0_:[*trans*-1,2-cyclohexanediol]_0_:[PO]_0_ (0.025 mol % catalyst), 20 bar of CO_2_, 50 °C (Table 1 and Figure 3). These low catalyst loadings and low temperatures are highly demanding conditions and were selected to allow for direct comparison against the highest performing dinuclear catalysts in the field.^26,43,44^ *trans*-1,2-Cyclohexanediol was added as a chain transfer agent to control polymer molecular weight and ensure delivery of hydroxyl-end groups.^47,48^ The catalyst loading for L_2_Co­(III)­Na­(I) was calculated for the formula unit C_24_H_26_CoN_2_NaO_9_ (*M*_r_ = 568.40 g mol^–1^), which is consistent with the solid-state characterization (by single-crystal XRD, Figure 5, and elemental analysis, see the Supporting Information for more detailed discussion, Figure S40).

**Table 1:** Data for PO/CO_2_ ROCOP Using L_*n*_Co­(III)­Na­(I) Catalysts (*n* = 1–4)^a^

entry	catalyst	overall TON^b^	CO_2_ Sel./%^c^	PPC Sel./%^d^	TOF_PPC_/h^–1^ ^e^	*k*_obs_/10^–6^ s^–1^ ^f^	*M*_*n*_ [*Đ*]/g mol^–1^ ^g^
1	L_1_Co(III)Na(I)	1577 ± 32	>99	97 ± 1	263 ± 20	20.65 ± 1.59	6600 [1.05]
2	L_2_Co(III)Na(I)	588 ± 21	>99	96 ± 1	76 ± 1	5.99 ± 0.03	3500 [1.05]
3	L_3_Co(III)Na(I)	568 ± 32	96	81 ± 1	49 ± 4	3.82 ± 0.33	2100 [1.05]
4	L_4_Co(III)Na(I)	28 ± 2	>99	0	0	0	0

a Conditions: catalyst (0.025 mol %, 3.6 mM), PO (5 mL, 14.3 M, neat), *trans*-1,2-cyclohexanediol (0.5 mol %, 71 mM), 20 bar CO_2_, 50 °C. All reactions were conducted in duplicate and errors calculated using SE = σ/√*n* (where σ = standard deviation and *n* = number of samples).

b Overall turnover number (TON) = moles of PO consumed/moles of catalyst, calculated from the relative integrals in the ^1^H NMR spectrum of PPC (4.99 ppm, 1 H), PC (4.84 ppm, 1 H), and PPO (3.46–3.75 ppm, 3 H) using mesitylene as an internal standard (6.80 ppm).

c CO_2_ selectivity determined by the relative integrals in the ^1^H NMR spectrum of PPC (4.99 ppm, 1 H) and PC (4.84 ppm, 1 H) compared with PPO (3.46–3.75 ppm, 3 H).

d PPC selectivity determined by the relative integrals in the ^1^H NMR spectrum of PPC (4.99 ppm, 1 H) against PC (4.84 ppm, 1 H). Reported at a common PO conversion (15%) in all cases.

e Turnover frequency to PPC (TOF_PPC_) calculated from *k*_obs_ (Tables S1 and S2, eq S1).

f *k*_obs_ determined from the linear region of the plot of ln­([PO]_*t*_/[PO]_0_) vs time.

g Determined by GPC analysis, in THF, calibrated with narrow-M_*n*_ polystyrene standards; dispersity values in parentheses.

All reactions were conducted in duplicate, showing highly reproducible results and small errors in activity (<10%) and selectivity values (<2%) (Table 1). Catalysts L_1_Co­(III)­Na­(I), L_2_Co­(III)­Na­(I), and L_3_Co­(III)­Na­(I) produce polycarbonates with high CO_2_ incorporation (>96% carbonate linkages) within the targeted molecular weight range (1–10 kg mol^–1^). Polymer molecular weights are close to the theoretical values, and all GPC traces are monomodal with narrow dispersities (*Đ* = 1.05), indicative of well-controlled polymerization catalysis (Table 1, entries 1–3, Figure 3, and Table S3). In the case of L_1_Co­(III)­Na­(I) and L_2_Co­(III)­Na­(I), selectivities for polymer over the cyclic carbonate side product are very high at 50 °C: 97 and 96%, respectively (Table 1, entries 1 and 2).

Reaction rates were monitored by *operando* ATR-IR spectroscopy, observing the increase in the intensity of a peak at 1750 cm^–1^, corresponding to the C=O stretch in the polycarbonate, and a peak at 1810 cm^–1^, corresponding to the C=O stretch in the cyclic carbonate side product. IR absorbance values were calibrated to conversion via integration of the ^1^H NMR spectrum of an aliquot taken at the end of the reaction. Semilogarithmic plots of ln­([PO]_*t*_/[PO]_0_) versus time are linear, with the observed pseudo first order rate constant, *k*_obs_, calculated from the modulus of the gradient (Figure 3). Comparison of the observed catalytic activities reveals significant differences across the series, with L_1_Co­(III)­Na­(I) performing the best and showing *k*_obs_ = 20.65 ± 1.59 × 10^–6^ s^–1^, which equates to a turnover frequency for polycarbonate, TOF_PPC_, of 263 ± 20 h^–1^ and a polymer selectivity of 97 ± 1% (Table 1, entry 1). Catalyst activity, in terms of *k*_obs_ and TOF_PPC_, decreased across the remainder of the series in the following order: L_2_Co­(III)­Na­(I), L_3_Co­(III)­Na­(I), and L_4_Co­(III)­Na­(I) (Table 1, entries 2–4).

Catalyst tolerance to impurities commonly found in commercial epoxides, such as water and diols, is a key feature of high-performance, industrially applicable catalysts as monomer purification is time-consuming and energy intensive. The tolerance of the three active catalysts, L_1_Co­(III)­Na­(I), L_2_Co­(III)­Na­(I), and L_3_Co­(III)­Na­(I), was tested using unpurified (non-distilled) PO at 50 °C and 20 bar CO_2_ (Figure 4 and Table S5). All reactions were run in duplicate, and errors in activity and selectivity were small (Table S5).

For L_1_Co­(III)­Na­(I), L_2_Co­(III)­Na­(I), and L_3_Co­(III)­Na­(I), the catalytic activity and selectivity were well maintained for ROCOP with unpurified PO (Figure 4 and Table S5). Despite the use of unpurified epoxide, the experimental polymer molecular weights were close to the theoretical values for L_1_Co­(III)­Na­(I) and L_2_Co­(III)­Na­(I) (Table S5). In contrast, for L_3_Co­(III)­Na­(I), the experimental molecular weight was significantly reduced relative to the theoretical molecular weight (Table S5, Δ*M*_*n*_ = *M*_theo_ *–* *M*_*n*_ = 2900 g mol^–1^). This same discrepancy was not found when the experimental and theoretical molecular weights for the copolymerization catalyzed by L_3_Co­(III)­Na­(I) were compared using purified PO (Δ*M*_*n*_ = *M*_theo_ – *M*_*n*_ = 200 g mol^–1^, Table S3). This highlights that the most robust catalysts are L_1_Co­(III)­Na­(I) and L_2_Co­(III)­Na­(I), relative to L_3_Co­(III)­Na­(I), in terms of tolerance to residual impurities present in the unpurified epoxide.

L_1_Co­(III)­Na­(I), L_2_Co­(III)­Na­(I), and L_3_Co­(III)­Na­(I) were also tested for PO/CO_2_ ROCOP at an elevated temperature of 70 °C, with all other conditions remaining the same (1:20:4000 [catalyst]_0_:[*trans*-1,2-cyclohexanediol]_0_:[PO]_0_, 20 bar CO_2_,) (Figure 4 and Table S4). Increasing the reaction temperature enables higher rates of polymer formation to be achieved but usually results in some compromise to polymer selectivity as formation of the stable cyclic carbonate side product becomes increasingly favored. It is also known that Co­(III)­(salen)­X/PPNX catalysts are susceptible to thermal deactivation to an inactive Co­(II) species, which places an upper limit on operating temperatures and limits the rates that can be achieved.^49,50^ At higher temperature, L_2_Co­(III)­Na­(I) produces polycarbonate with higher activity, achieving a TOF_PPC_ of 275 ± 17 h^–1^, with a polymer selectivity of 85% (Table S4), while L_3_Co­(III)­Na­(I) produces only cyclic carbonate. However, L_1_Co­(III)­Na­(I) shows excellent performances as it achieves both high activity, with TOF_PPC_ = 1428 ± 94 h^–1^, and high polymer selectivity of 98% (Table S4 and Figure 4).

## Discussion

Testing under three different sets of conditions reveals L_1_Co­(III)­Na­(I) as the best catalyst of the series (Table 1 and Tables S3–S5). It is clear that L_1_Co­(III)­Na­(I) exhibits the most optimal coordination chemistry and ligand structure for the catalysis, and that relatively small modifications to the ancillary ligands significantly influence the rate and selectivity-determining steps (Figure 5).

Comparison of the solid-state structures of the catalysts, obtained by single-crystal X-ray diffraction experiments, reveals no obvious correlation of catalytic performance with any single bond distance, including intermetallic separation or distance at which Na­(I) sits above the plane defined by the ether “O” donors (Figure 6). However, the two catalysts with the ethylene (C2) di-imine linker perform better than the two catalysts with the propylene (C3) linker. The effect of the diamine linker is clear when examining the solid-state structures, as the Co–N distances are significantly longer in the propylene-linker catalysts than the (higher-performance) ethylene-linker catalysts (1.955(2) Å (L_3_Co­(III)­Na­(I)) and 1.927(3) Å (L_4_Co­(III)­Na­(I)) versus 1.893(3) Å (L_1_Co­(III)­Na­(I)) and 1.884(2) Å (L_3_Co­(III)­Na­(I)), Figure 6). The Co–O distances are also significantly longer in the propylene-linker catalysts versus those in the ethylene-linker catalysts (1.916(1) Å (L_3_Co­(III)­Na­(I)) and 1.912(2) Å (L_4_Co­(III)­Na­(I)) versus 1.895(2) Å (L_1_Co­(III)­Na­(I)) and 1.890(2) Å (L_2_Co­(III)­Na­(I)) Figure 6). The variation in these bond distances indicates that steric effects, in the vicinity of Co­(III), seem to be important in mediating catalyst performance.

**Figure 6:**
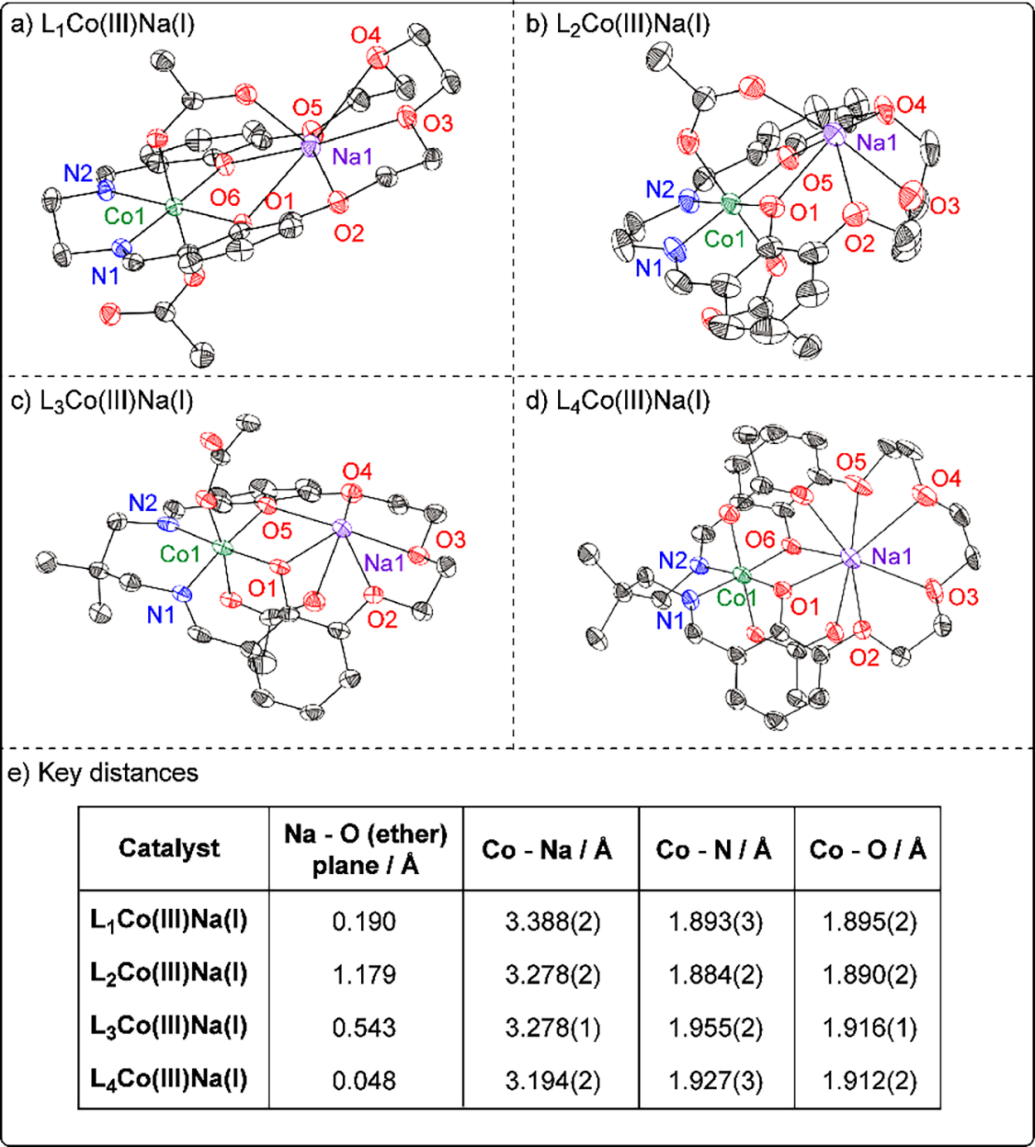
(a–d) Molecular structures of L_*n*_Co­(III)­Na­(I) (*n* = 1–4) obtained from single-crystal X-ray diffraction experiments. Hydrogen atoms and outer-sphere solvent molecules have been omitted for clarity. Thermal ellipsoids are represented at 40% probability. (e) Table of key distances. Crystal structures for L_1_Co­(III)­Na­(I) and L_4_Co­(III)­Na­(I) are previously reported.^43,46^

This observation prompted investigation into the buried volume (*V*_bur_) surrounding the Co­(III) center, using the SambVca 2.1 web tool, from the available XRD data.^51–55^ The value for *V*_bur_ was calculated using a sphere centered at Co­(III) of radius 5 Å and topographical maps for each catalyst calculated, as viewed along the likely direction of approach of the epoxide toward Co­(III) (Tables S12 and S13; Figures S41 and S42). Total *V*_bur_ was found to loosely correlate with observed catalytic activity, with the highest performing catalyst showing the lowest Co­(III) *V*_bur_ and highest free volume, *V*_free_ (Figure S40).

The rate-determining step in the catalytic mechanism is proposed to involve ring opening of a Co­(III)-activated epoxide by a Na­(I)-bound carbonate nucleophile, where the distinct roles of each metal are supported by prior computational and experimental investigations (Figure 5).^23,38,42,43^ It is tentatively proposed that increased free space (decreased *V*_bur_) around Co­(III) facilitates epoxide coordination and activation toward ring opening, implying that rate differences across the series are affected by variations in the sterics of the ligand macrocycle. However, the proposal is early stage since the absolute differences in *V*_bur_ across the series are small, ranging from 51 to 57%, implying that the sterics of the diamine linker is one of several factors giving rise to the observed performance differences.

The ligand macrocycle size, and its effect on the positioning of metals within the ligand as indicated by the solid-state structures, could also affect catalytic activity by influencing the degree of ligand-to-metal electron donation and labilizing the Na–O bond toward ring opening of PO (Figure 5).

Selectivity for polymer (PPC) over cyclic carbonate (PC) was found to vary across the catalyst series (97 and 96 ± 1% for L_1_Co­(III)­Na­(I) and L_2_Co­(III)­Na­(I), 81 ± 1% for L_3_Co­(III)­Na­(I), and 0% for L_4_Co­(III)­Na­(I), Table 1, entries 1–4). DFT and experimental investigations on a related catalyst indicate that back-biting occurs from the alkoxide intermediate, and the activation barriers to epoxide ring opening vs back-biting processes are similar.^23,44^ Therefore, the relative concentrations of the alkoxide and carbonate intermediates (controlled by the CO_2_ insertion equilibrium) are important in determining the selectivity (Figure 5). The finding here that polymer selectivity is catalyst dependent implies the nature of the ligand macrocycle affects the alkoxide-carbonate equilibrium, where the most selective catalysts are proposed to bias the equilibrium position toward the carbonate.

Increasing the temperature to 70 °C results in a drop in polymer selectivity for L_2_Co­(III)­Na­(I) and L_3_Co­(III)­Na­(I) (85 ± 1% for L_2_Co­(III)­Na­(I), 0% for L_3_Co­(III)­Na­(I), Table S4), while high polymer selectivity is retained for L_1_Co­(III)­Na­(I) (98 ± 1%, Table S4). It is tentatively proposed that the reduced selectivity can be accounted for by a change in equilibrium constant with rising temperature, biasing the position of equilibrium toward the alkoxide intermediate and favoring cyclic carbonate formation (the thermodynamic product). The most selective catalyst (L_1_Co­(III)­Na­(I)) strongly favors the carbonate intermediate and is, therefore, least sensitive to the change in equilibrium position with temperature, retaining high polymer selectivity at 70 °C.

The performance of the best catalyst L_1_Co­(III)­Na­(I) under the lead conditions, 20 bar of CO_2_, 70 °C, and 0.025 mol % catalyst loading, can be compared against field-leading literature Co­(III) catalysts for PO/CO_2_ ROCOP, under analogous or closely related reaction conditions (Figure 7 and Table S14). The most active catalyst in the field, a tethered Co­(III)-salen catalyst (**D**), which was first reported by Lee and co-workers in 2008, is significantly more active than all the other catalysts with a TOF = 19,000 h^–1^ and selectivity >99%.^26^ The lead catalyst in this work (L_1_Co­(III)­Na­(I), **A**) can also be compared against a catalyst system comprising a mixture of a Co­(III)­(salen)­X complex and K-18-crown-6 (**E**) (Figure 7).^40^ In this case, the catalysis was studied at 50 °C (rather than 70 °C), but a much higher catalyst loading was used (0.1 mol % catalyst) relative to **A**. Catalyst **A** performs significantly better, showing a TOF = 1428 h^–1^ and selectivity of 97%, relative to the mixture of species which shows a TOF = 231 h^–1^ and selectivity of 82%. The difference in performance highlights the benefits of the heterodinuclear complex, where the metals appear to show catalytic synergy. The better performance of **A** over the catalyst mixture (**E**) is further emphasized when comparing catalytic activity per gram of catalyst, where **A** shows a 10-fold enhancement in activity per gram relative to **E.**

**Figure 7:**
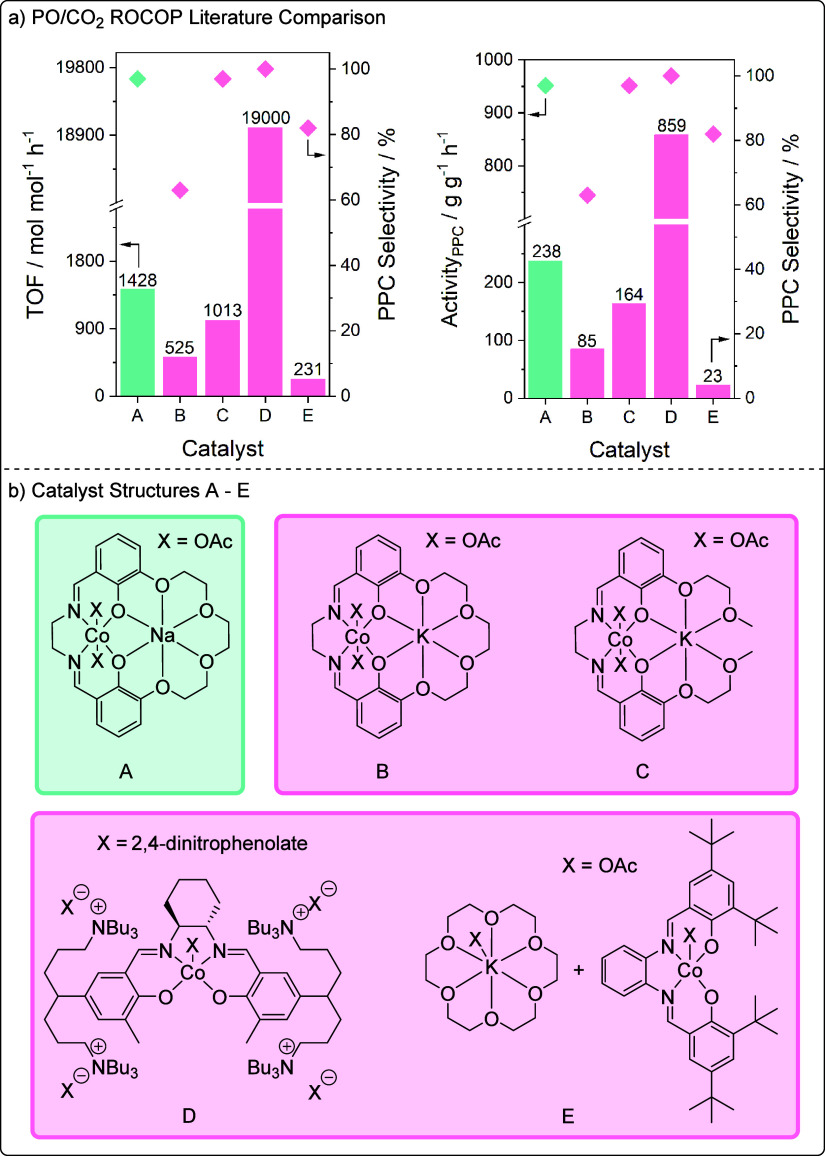
(a, b) Comparison of L_1_Co­(III)­Na­(I) (A) against high-performance literature Co­(III)-based PO/CO_2_ ROCOP catalysts (B–E). Conditions: 70 °C, 20 bar CO_2_, 0.025 mol % cat (A–C); 70–77 °C, 20 bar CO_2_, 0.001 mol % cat (D), 50 °C, 20 bar CO_2_ 0.1 mol % cat (E).^26,38,43,44^

It is also important to compare the performance of the lead catalyst in this work (**A**, Figure 7), against the previously reported Co­(III)­K­(I) catalysts, coordinated by the same or related ligands (**B** and **C**, Figure 7).^43,44^ Comparison to Co­(III)­K­(I) catalyst **B** reveals that, at 70 °C, **A** is more active. This improvement is clear when considering either the mol/mol of TOF or the gram/gram activity. At the higher temperature, **A** has a TOF_mol/mol_ = 1428 h^–1^ and TOF_g/g_= 238 h^–1^ compared to **B**, which shows TOF_mol/mol_ = 525 h^–1^ and TOF_g/g_= 85 h^–1^. The relative activities are different to those observed at the lower temperature of 50 °C, where the Co­(III)­K­(I) (**B**) catalyst is more active.^38,43^ Another clear benefit of using **A** is that it shows a much higher selectivity of 97% relative to **B**, which has a polymer selectivity of just 63% at 70 °C.^43^ The lower selectivity for **B** is responsible for its lower polymer activity value at higher temperature. Overall, the experiments clearly demonstrate the importance of testing catalysts under different conditions since Co­(III)­Na­(I) is the right metal combination for use at elevated temperatures, where the rates of polymer formation are higher than for related Co­(III)­K­(I) catalysts.

Further comparison to the “open” ligated Co­(III)­K­(I) catalyst **C**, the current highest performance heterodinuclear catalyst for PO/CO_2_ ROCOP, also highlights **A** as an excellent catalyst, able to compete with K­(I) complexes. Under the same high-temperature (70 °C) conditions, **A** outperforms the Co­(III)­K­(I) catalyst (**C**) in terms of TOF_mol/mol_ = 1428 h^–1^ (vs catalyst **C**, TOF_mol/mol_ = 1013 h^–1^) and TOF_g/g_ = 238 h^–1^ (vs catalyst **C**, TOF_g/g_ = 164 h^–1^). Overall, the lead macrocyclic complex in this work, **A**, shows 1.5× better performance than catalyst **C**. In the future, investigating the Co­(III)­Na­(I) complex at higher temperature using the open ancillary ligand is warranted.

## Conclusions

The synthesis and catalytic testing of a series of four Co­(III)­Na­(I) complexes were reported, with catalysts differing by the ligand structure and overall macrocycle size through variation of the di-imine linkers and number of oxygen donors. The ligand macrocycle modifications were found to exert a significant influence on propene oxide/carbon dioxide ring-opening copolymerization activity and selectivity, with the best catalyst being L_1_Co­(III)­Na­(I). When compared to other leading Co­(III) catalysts, L_1_Co­(III)­Na­(I) was unusual and useful since it showed high activity and selectivity at higher temperatures; under such conditions, it outperformed related Co­(III)­K­(I) catalysts. These findings motivate the continued exploration of the Co­(III)­Na­(I) catalyst for other epoxides and monomer combinations. They also suggest that other transition metal/sodium complexes should be developed so as to target highly active, tolerant, and selective catalysts for carbon dioxide utilizations.

## Materials and Methods

### General Procedures

All manipulations were carried out by using a dual manifold nitrogen-vacuum Schlenk line or a nitrogen-filled glovebox. Reagents were sourced commercially and used as received unless otherwise stated. The dialdehyde proligand for synthesis of L_1_Co­(III)­Na­(I) and L_4_Co­(III)­Na­(I) (3-(2-(2-[2-(3-formyl-2-hydroxyphenoxy)­ethoxy]­ethoxy)­ethoxy)-2-hydroxybenzaldehyde) was sourced from Enamine Ltd. and Manchester Organics Ltd. and used as received. The dialdehyde proligand for synthesis of L_2_Co­(III)­Na­(I) and L_3_Co­(III)­Na­(I) (2-[2-(3-formyl-2-hydroxy-phenoxy)­ethoxy]­ethoxy]-2-hydroxybenzaldehyde) was sourced from Fluorochem Ltd. and used as received. Propene oxide was purchased from Sigma Aldrich and dried over calcium hydride for 48 h before purifying by distillation and storing under nitrogen. Unpurified PO was purchased from Sigma Aldrich and used as received. Research-grade CO_2_ (BOC, 99.99%) was dried through a Drierlite column and two additional drying columns (Micro Torr, model number: MCI-804FV) in series prior to use.

#### NMR Spectroscopy

All NMR spectra were collected using a 500 MHz Bruker Avance III HD Nonobay spectrometer with a 11.75T magnet at 298 K. DOSY analysis was carried out on a 500 MHz Bruker Avance III spectrometer with a 11.75T magnet, with convection compensation. Diffusion coefficients were calculated from the Stejskal–Tanner expression using nonlinear regression analysis.

#### Attenuated Total Reflectance (ATR-IR) Spectroscopy

All PO/CO_2_ copolymerizations were monitored using a ReactIR 701 instrument, including a liquid nitrogen MCT detector and a DiComp ATR-IR probe, which was bottom-mounted onto a 100 mL Parr Reactor. IR spectra were generated once per minute, from 256 scans, and recorded within a wavenumber range of 3000–650 cm^–1^.

#### Gel Permeation Chromatography

GPC traces were obtained using a Shimadzu LC-20AD instrument at 40 °C equipped with a refractive index (RI) detector and two mixed bed PSS SDV linear S columns in series. The eluent used was tetrahydrofuran (THF) at a flow rate of 1 mL/min. Polymer molar mass values were calibrated with narrow molar mass polystyrene standards.

#### X-ray Crystallography

Crystals were isolated and immersed in perfluoropolyether oil (Fomblin). Single crystals were mounted on MiTiGen MicroMounts before being cooled to 150 K using an Oxford Cryosystems Cryostream. Data were collected on an Oxford Diffraction Supernova Diffractometer using Cu Kα (λ = 1.5417 Å) radiation. Data processing was carried out on CrysAlisPro software, and structures were solved using the SHELXT program, within the Olex2 system suite.

#### MALDI-TOF Mass Spectrometry

MALDI-TOF analysis was carried out on a Bruker Autoflex Speed MALDI-TOF spectrometer. 1:1:1 solutions of the catalyst (10 mg mL^–1^ in methanol) to *trans*-1-[3-(4-*tert*-butylphenyl)-2-methyl-2-propenylidene]-malonitrile (10 mg mL^–1^ in tetrahydrofuran) to sodium iodide (10 mg mL^–1^ in methanol) were prepared. These solutions were spotted three times each on the MALDI plate and allowed to dry overnight before performing analysis.

#### Elemental Analysis

Elemental analysis was carried out by the London Metropolitan University (166-220 Holloway Road, London, N7 8DB) and the micro-analysis service at the University of York (Heslington, York, YO10 5DD).

### Catalyst Synthesis

#### L_1_Co­(III)­Na­(I) Catalyst Synthesis

An acetonitrile (15 mL) solution of the relevant dialdehyde proligand (400 mg, 1.02 mmol), Na­(OAc) (84 mg, 1.02 mmol), and Co­(OAc)_2_ (181 mg, 1.02 mmol) was prepared in a nitrogen glovebox. The solution was stirred for 1 h followed by addition of ethylene diamine (68 μL, 1.02 mmol), in one portion, under a flow of nitrogen. The resulting solution was left to stir for a further 16 h before exposing to air and adding two equivalents of acetic acid (117 μL, 2.04 mmol). After stirring for 72 h, the solvent was removed *in vacuo* to obtain a dark brown, glassy solid. Purification of the crude product was achieved via azeotropic distillation with toluene (3 × 50 mL) and hexane (3 × 50 mL), followed by precipitation of the complex from a dichloromethane solution with pentane. The resulting solid was dried *in vacuo* overnight and stored in a nitrogen glovebox (73% yield).

#### L_2_Co­(III)­Na­(I) Catalyst Synthesis

A methanol solution (125 mL) of ethylene diamine (77 μL, 1.16 mmol) was added dropwise, over the course of 3 h, to a refluxing methanol solution (500 mL) of the relevant dialdehyde proligand (400 mg, 1.16 mmol) and Na­(OAc) (95 mg, 1.16 mmol) under nitrogen. The solution was left to cool to room temperature before adding Co­(OAc)_2_ (205 mg, 1.16 mmol) and then left to stir for a further 16 h under nitrogen. Subsequently, the solution was exposed to air and one equivalent of acetic acid was added (66 μL, 1.16 mmol). The solution was stirred for a further 72 h. The solvent was then removed *in vacuo* to obtain a dark brown glassy solid. Purification of the crude product was achieved via azeotropic distillation with toluene (3 × 50 mL) and hexane (3 × 50 mL), followed by precipitation of the complex from a dichloromethane solution with pentane. The resulting solid was dried *in vacuo* overnight and stored in a nitrogen glovebox.

#### L_3_Co­(III)­Na­(I) Catalyst Synthesis

A methanol solution (125 mL) of 2,2-dimethylpropane-1,3-diamine (139 μL, 1.16 mmol) was added dropwise, over the course of 3 h, to a refluxing methanol solution (500 mL) of the relevant dialdehyde proligand (400 mg, 1.16 mmol) and Na­(OAc) (95 mg, 1.16 mmol) under nitrogen. The solution was left to cool to room temperature before adding Co­(OAc)_2_ (205 mg, 1.16 mmol) and then left to stir for a further 16 h under nitrogen. Subsequently, the solution was exposed to air, and one equivalent of acetic acid was added (66 μL, 1.16 mmol). The solution was stirred for an additional 72 h. The solvent was then removed *in vacuo* to obtain a dark brown glassy solid. Purification of the crude product was achieved via azeotropic distillation with toluene (3 × 50 mL) and hexane (3 × 50 mL), followed by precipitation of the complex from a dichloromethane solution with pentane. The resulting solid was dried *in vacuo* overnight and stored in a nitrogen glovebox (63% yield).

#### L_4_Co­(III)­Na­(I) Catalyst Synthesis

A methanol solution (125 mL) of 2,2-dimethylpropane-1,3-diamine (123 μL, 1.02 mmol) was added dropwise, over the course of 3 h, to a refluxing methanol solution (500 mL) of the relevant dialdehyde proligand (400 mg, 1.02 mmol) and Na­(OAc) (84 mg, 1.02 mmol) under nitrogen. The solution was left to cool to room temperature before adding Co­(OAc)_2_ (181 mg, 1.02 mmol) and then left to stir for a further 16 h under nitrogen. Subsequently, the solution was exposed to air, and one equivalent of acetic acid was added (59 μL, 1.02 mmol). The solution was stirred for a further 72 h. The solvent was then removed *in vacuo* to obtain a dark brown glassy solid. Purification of the crude product was achieved via azeotropic distillation with toluene (3 × 50 mL) and hexane (3 × 50 mL), followed by precipitation of the complex from a dichloromethane solution with pentane. The resulting solid was dried *in vacuo* overnight and stored in a nitrogen glovebox (13% yield).

### Polymerization Procedure

A solution of catalyst (0.018 mmol) and *trans*-1,2-cyclohexanediol (42 mg, 0.36 mmol) in PO (5 mL, 72 mmol) was prepared in a nitrogen glovebox. Mesitylene (50 μL, 0.357 mmol) was added as an internal standard. The reaction solution was injected into a 100 mL Parr Reactor, fitted with a bottom-mounted DiComp IR probe, under a flow of CO_2_. The vessel was pressurized to 20 bar CO_2_ before heating to the desired temperature. Conversion to polycarbonate and cyclic carbonate was followed using *operando* ATR-IR spectroscopy by monitoring the increase in intensity of a peak at 1750 and 1810 cm^–1^, respectively. Subsequently, the reaction was allowed to cool and quenched by exposure to air and the addition of benzoic acid (2 mg, 0.018 mmol).

## Supplementary Material





## Notes

Catalyst characterization data (NMR spectroscopy, X-ray crystallography, MALDI-TOF mass spectrometry, and elemental analysis); polymerization data (GPC chromatograms, NMR spectrum of polymerization aliquot, and tables and equations for calculating turnover frequencies) (PDF)
Crystal data (CIF)

## #

David H Koch Institute for Integrative Cancer Research, Massachusetts Institute of Technology, Cambridge, Massachusetts 02139, USA

## References

[1] Vidal F., van der Marel E. R., Kerr R. W. F., McElroy C., Schroeder N., Mitchell C., Rosetto G., Chen T. T. D., Bailey R. M., Hepburn C. (2024). Designing a circular carbon and plastics economy for
a sustainable future. Nature.

[2] LeClerc H. O., Erythropel H. C., Backhaus A., Lee D. S., Judd D. R., Paulsen M. M., Ishii M., Long A., Ratjen L., Gonsalves
Bertho G. (2025). The CO_2_ Tree: The Potential
for Carbon Dioxide Utilization Pathways. ACS
Sustainable Chem. Eng..

[3] Leitner W. (2024). Carbon dioxide
and hydrogen as building blocks for a sustainable interface of energy
and chemistry. Philos. Trans. R. Soc., A.

[4] Vogt E. T. C., Weckhuysen B. M. (2024). The refinery
of the future. Nature.

[5] Bachmann M., Zibunas C., Hartmann J., Tulus V., Suh S., Guillén-Gosálbez G., Bardow A. (2023). Towards circular plastics
within planetary boundaries. Nat. Sustain..

[6] Meys R., Kätelhön A., Bachmann M., Winter B., Zibunas C., Suh S., Bardow A. (2021). Achieving net-zero
greenhouse gas emission plastics by a circular carbon economy. Science.

[7] Klos N., Osterthun O., Mengers H. G., Lanzerath P., Graf von Westarp W., Lim G., Gausmann M., Küsters-Spöring J.-D., Wiesenthal J., Guntermann N. (2024). Concatenating Microbial,
Enzymatic, and Organometallic Catalysis for Integrated Conversion
of Renewable Carbon Sources. JACS Au.

[8] Tang S., Lin B.-L., Tonks I., Eagan J. M., Ni X., Nozaki K. (2024). Sustainable
Copolymer Synthesis from Carbon Dioxide
and Butadiene. Chem. Rev..

[9] Bhat G. A., Darensbourg D. J. (2023). Coordination
complexes as catalysts for the coupling
reactions of oxiranes and carbon dioxide. Coord.
Chem. Rev..

[10] Lidston C. A. L., Severson S. M., Abel B. A., Coates G. W. (2022). Multifunctional
Catalysts for Ring-Opening Copolymerizations. ACS Catal..

[11] Diment W. T., Lindeboom W., Fiorentini F., Deacy A. C., Williams C. K. (2022). Synergic
Heterodinuclear Catalysts for the Ring-Opening Copolymerization (ROCOP)
of Epoxides, Carbon Dioxide, and Anhydrides. Acc. Chem. Res..

[12] Cao H., Liu S., Wang X. (2022). Environmentally
benign metal catalyst for the ring-opening
copolymerization of epoxide and CO_2_: state-of-the-art,
opportunities, and challenges. Green Chem. Eng..

[13] Yang G.-W., Xie R., Zhang Y.-Y., Xu C.-K., Wu G.-P. (2024). Evolution of Copolymers
of Epoxides and CO_2_: Catalysts, Monomers, Architectures,
and Applications. Chem. Rev..

[14] Sasanuma Y., Takahashi Y. (2017). Structure-Property
Relationships of Poly­(ethylene carbonate)
and Poly­(propylene carbonate). ACS Omega.

[15] Langanke J., Wolf A., Hofmann J., Böhm K., Subhani M. A., Müller T. E., Leitner W., Gürtler C. (2014). Carbon dioxide
(CO_2_) as sustainable feedstock for polyurethane production. Green Chem..

[16] Lee S. H., Cyriac A., Jeon J. Y., Lee B. Y. (2012). Preparation of thermoplastic
polyurethanes using in situ generated poly­(propylene carbonate)-diols. Polym. Chem..

[17] Alagi P., Ghorpade R., Choi Y. J., Patil U., Kim I., Baik J. H., Hong S. C. (2017). Carbon
Dioxide-Based Polyols as Sustainable
Feedstock of Thermoplastic Polyurethane for Corrosion-Resistant Metal
Coating. ACS Sustainable Chem. Eng..

[18] Schwarz D. B., Cavicchi K. A., Eagan J. M. (2024). Carbon
Dioxide-Derived Poly­(Propylene
Carbonate) Bottlebrush Polymers: Synthesis, Viscoelastic Properties,
and Degradation. Macromolecules.

[19] Dong J., Zhou J., Zhang X., Liu B., Liu S., Wang X. (2024). Construction of Ultralow-Molecular-Weight CO_2_–Polyols
with Self-Catalytic Performance in Polyurethane Preparation. Macromolecules.

[20] Propylene Oxide Market Analysis https://www.chemanalyst.com/industry-report/propylene-oxide-po-market-755 (accessed 30/10/2024)

[21] Fan P., Liu S., Zhang R., Zhuo C., Gao F., Pang X., Chen X., Wang X. (2024). Rigid-Flexible Binuclear Catalysts:
Boosting Activity for Copolymerization of CO2 and Propylene Oxide. Macromolecules.

[22] Yang L., Liu S., Zhou Z., Zhang R., Zhou H., Zhuo C., Wang X. (2024). Aggregate
Catalysts: Regulating Multimetal Cooperativity for CO2/Epoxide
Copolymerization. Macromolecules.

[23] Deacy A. C., Phanopoulos A., Lindeboom W., Buchard A., Williams C. K. (2022). Insights
into the Mechanism of Carbon Dioxide and Propylene Oxide Ring-Opening
Copolymerization Using a Co­(III)/K­(I) Heterodinuclear Catalyst. J. Am. Chem. Soc..

[24] Cohen C. T., Chu T., Coates G. W. (2005). Cobalt
Catalysts for the Alternating Copolymerization
of Propylene Oxide and Carbon Dioxide: Combining High Activity and
Selectivity. J. Am. Chem. Soc..

[25] Nakano K., Kamada T., Nozaki K. (2006). Selective
Formation of Polycarbonate
over Cyclic Carbonate: Copolymerization of Epoxides with Carbon Dioxide
Catalyzed by a Cobalt­(III) Complex with a Piperidinium End-Capping
Arm. Angew. Chem., Int. Ed..

[26] Sujith S, Min J. K., Seong J. E., Na S. J., Lee B. Y. (2008). A Highly
Active and Recyclable Catalytic System for CO_2_/Propylene
Oxide Copolymerization. Angew. Chem. Int. Ed..

[27] Seo Y. H., Hyun Y. B., Lee H. J., Lee H. C., Lee J. H., Jeong S. M., Lee B. Y. (2021). CO_2_/Propylene Oxide Copolymerization
with a Bifunctional Catalytic System Composed of Multiple Ammonium
Salts and a Salen Cobalt Complex Containing Sulfonate Anions. Macromol. Res..

[28] Duan R., Hu C., Sun Z., Zhang H., Pang X., Chen X. (2019). Conjugated
tri-nuclear salen-Co complexes for the copolymerization of epoxides/CO_2_: cocatalyst-free catalysis. Green Chem..

[29] Hatazawa M., Nakabayashi K., Ohkoshi S.-i., Nozaki K. (2016). In Situ Generation
of CoIII–Salen Complexes for Copolymerization of Propylene
Oxide and CO_2_. Chem. Eur. J..

[30] Liu Y., Ren W.-M., Liu C., Fu S., Wang M., He K.-K., Li R.-R., Zhang R., Lu X.-B. (2014). Mechanistic
Understanding of Dinuclear Cobalt­(III) Complex Mediated Highly Enantioselective
Copolymerization of meso-Epoxides with CO_2_. Macromolecules.

[31] Liu J., Ren W.-M., Liu Y., Lu X.-B. (2013). Kinetic Study on
the Coupling of CO_2_ and Epoxides Catalyzed by Co­(III) Complex
with an Inter- or Intramolecular Nucleophilic Cocatalyst. Macromolecules.

[32] Seong J. E., Na S. J., Cyriac A., Kim B.-W., Lee B. Y. (2010). Terpolymerizations
of CO_2_, Propylene Oxide, and Various Epoxides Using a Cobalt­(III)
Complex of Salen-Type Ligand Tethered by Four Quaternary Ammonium
Salts. Macromolecules.

[33] Ren W.-M., Liu Z.-W., Wen Y.-Q., Zhang R., Lu X.-B. (2009). Mechanistic
Aspects of the Copolymerization of CO_2_ with Epoxides Using
a Thermally Stable Single-Site Cobalt­(III) Catalyst. J. Am. Chem. Soc..

[34] Cohen C. T., Coates G. W. (2006). Alternating copolymerization
of propylene oxide and
carbon dioxide with highly efficient and selective (salen)­Co­(III)
catalysts: Effect of ligand and cocatalyst variation. J. Polym. Sci., Part A: Polym. Chem..

[35] Nakano K., Hashimoto S., Nozaki K. (2010). Bimetallic mechanism operating in
the copolymerization of propylene oxide with carbon dioxide catalyzed
by cobalt–salen complexes. Chem. Sci..

[36] Qin Z., Thomas C. M., Lee S., Coates G. W. (2003). Cobalt-Based Complexes
for the Copolymerization of Propylene Oxide and CO2: Active and Selective
Catalysts for Polycarbonate Synthesis. Angew.
Chem., Int. Ed..

[37] Nagae H., Matsushiro S., Okuda J., Mashima K. (2023). Cationic tetranuclear
macrocyclic CaCo3 complexes as highly active catalysts for alternating
copolymerization of propylene oxide and carbon dioxide. Chem. Sci..

[38] Fiorentini F., Diment W. T., Deacy A. C., Kerr R. W. F., Faulkner S., Williams C. K. (2023). Understanding catalytic
synergy in dinuclear polymerization
catalysts for sustainable polymers. Nat. Commun..

[39] Lindeboom W., Deacy A. C., Phanopoulos A., Buchard A., Williams C. K. (2023). Correlating
Metal Redox Potentials to Co­(III)­K­(I) Catalyst Performances in Carbon
Dioxide and Propene Oxide Ring Opening Copolymerization. Angew. Chem., Int. Ed..

[40] Fiorentini F., Eisenhardt K. H. S., Deacy A. C., Williams C. K. (2024). Synergic Catalysis:
the Importance of Intermetallic Separation in Co­(III)­K­(I) Catalysts
for Ring Opening Copolymerizations. J. Am. Chem.
Soc..

[41] Eisenhardt K. H. S., Fiorentini F., Williams C. K. (2024). Understanding the Effect of M­(III)
Choice in Heterodinuclear Polymerization Catalysts. Inorg. Chem..

[42] Butler F., Fiorentini F., Eisenhardt K. H. S., Williams C. K. (2025). Structure-Activity
Relationships for s-Block Metal/Co­(III) Heterodinuclear Catalysts
in Cyclohexene Oxide Ring-Opening Copolymerizations. Angew. Chem., Int. Ed..

[43] Deacy A. C., Moreby E., Phanopoulos A., Williams C. K. (2020). Co­(III)/Alkali-Metal­(I)
Heterodinuclear Catalysts for the Ring-Opening Copolymerization of
CO_2_ and Propylene Oxide. J. Am. Chem.
Soc..

[44] Eisenhardt K. H. S., Fiorentini F., Lindeboom W., Williams C. K. (2024). Quantifying CO_2_ Insertion Equilibria for Low-Pressure Propene Oxide and Carbon
Dioxide Ring Opening Copolymerization Catalysts. J. Am. Chem. Soc..

[45] Haynes, W. M. CRC Handbook of Chemistry and Physics; CRC Press, 2014.

[46] Lindeboom W., Fraser D. A. X., Durr C. B., Williams C. K. (2021). Heterodinuclear
Zn­(II), Mg­(II) or Co­(III) with Na­(I) Catalysts for Carbon Dioxide
and Cyclohexene Oxide Ring Opening Copolymerizations. Chem. Eur. J..

[47] von
der Assen N., Bardow A. (2014). Life cycle assessment of polyols
for polyurethane production using CO_2_ as feedstock: insights
from an industrial case study. Green Chem..

[48] Sternberg A., Jens C. M., Bardow A. (2017). Life cycle assessment of CO_2_-based C1-chemicals. Green Chem..

[49] Anderson C. E., Vagin S. I., Hammann M., Zimmermann L., Rieger B. (2013). Copolymerisation of Propylene Oxide
and Carbon Dioxide
by Dinuclear Cobalt Porphyrins. ChemCatChem.

[50] Xia W., Salmeia K. A., Vagin S. I., Rieger B. (2015). Concerning the Deactivation
of Cobalt­(III)-Based Porphyrin and Salen Catalysts in Epoxide/CO2
Copolymerization. Chem. Eur. J..

[51] Falivene L., Cao Z., Petta A., Serra L., Poater A., Oliva R., Scarano V., Cavallo L. (2019). Towards the online computer-aided
design of catalytic pockets. Nat. Chem..

[52] Falivene L., Credendino R., Poater A., Petta A., Serra L., Oliva R., Scarano V., Cavallo L. (2016). SambVca 2. A Web Tool
for Analyzing Catalytic Pockets with Topographic Steric Maps. Organomet..

[53] Poater A., Ragone F., Giudice S., Costabile C., Dorta R., Nolan S. P., Cavallo L. (2008). Thermodynamics
of N-Heterocyclic
Carbene Dimerization: The Balance of Sterics and Electronics. Organomet..

[54] Poater A., Ragone F., Mariz R., Dorta R., Cavallo L. (2010). Comparing
the Enantioselective Power of Steric and Electrostatic Effects in
Transition-Metal-Catalyzed Asymmetric Synthesis. Chem. Eur. J..

[55] Karnes J. P., Kumar A., Hopkins
Leseberg J. A., Day V. W., Blakemore J. D. (2024). Trivalent
Cations Slow Electron Transfer to Macrocyclic Heterobimetallic Complexes. Inorg. Chem..

