# Supplementary material for: Heterodinuclear Co(III)Na(I) Catalysts for the Ring-Opening Copolymerization of Propene Oxide and Carbon Dioxide
Source: Macromolecules. 2025 Jul 4;58(14):7150–60. doi: 10.1021/acs.macromol.5c01529 (PMC12288079; doi:10.1021/acs.macromol.5c01529)
Supplement: Supplementary file 1 [file ma5c01529_si_001.pdf]

Supporting Information

Heterodinuclear Co(III)Na(I) Catalysts for the Ring-Opening Copolymerization of Propene Oxide and Carbon Dioxide

Frederica Butler[+], Francesca Fiorentini[+], Katharina H. S. Eisenhardt, Charlotte K. Williams*

*charlotte.williams@chem.ox.ac.uk

[+] F. Butler and F. Fiorentini contributed equally to the work and are listed as co-first authors

Department of Chemistry, University of Oxford, 12 Mansfield Road, OX1 3TA, United Kingdom

Figure S1. ^1H NMR spectrum of $L_1Co(III)Na(I)$ 7

Figure S2. $^{13}C\{^1H\}$ NMR spectrum of $L_1Co(III)Na(I)$ 7

Figure S3. ^1H-^1H COSY NMR spectrum of $L_1Co(III)Na(I)$ 8

Figure S4. ^1H-^{13}C HSQC NMR spectrum of $L_1Co(III)Na(I)$ 8

Figure S5. ^1H-^{13}C HMBC NMR spectrum of $L_1Co(III)Na(I)$ 9

Figure S6. ^1H NMR spectrum of $L_2Co(III)Na(I)$, assigned according to the presence of two species ... 9

Figure S7. $^{13}C\{^1H\}$ NMR spectrum of $L_2Co(III)Na(I)$, assigned according to the presence of two species 10

Figure S8. ^1H -^1H COSY NMR spectrum of $L_2Co(III)Na(I)$ 10

Figure S9. ^1H -^{13}C HSQC NMR spectrum of $L_2Co(III)Na(I)$ 11

Figure S10. ^1H -^{13}C HMBC NMR spectrum of $L_2Co(III)Na(I)$ 11

Figure S11. ^1H DOSY NMR spectrum of $L_2Co(III)Na(I)$ in MeCN-d_3, showing the presence of two species 12

Figure S12. Mass spectrum (MALDI-TOF) of $L_2Co(III)Na(I)$, showing the presence of two species ($[L_2Co(II)Na(I)]^+$ and $[(L_2Co(II))_2Na(I)]^+$) 12

Figure S13. Variable Temperature NMR spectra of $L_2Co(III)Na(I)$ in MeCN-d_3 13

Figure S14. ^1H NMR spectrum of $L_3Co(III)Na(I)$ 13

Figure S15. $^{13}C\{^1H\}$ NMR spectrum of $L_3Co(III)Na(I)$ 14

Figure S16. ^1H-^1H COSY NMR spectrum of $L_3Co(III)Na(I)$ 14

Figure S17. ^1H -^{13}C HSQC NMR spectrum of $L_3Co(III)Na(I)$ 15

Figure S18. ^1H-^{13}C HMBC NMR spectrum of $L_3Co(III)Na(I)$ 15

Figure S19. ^1H DOSY NMR spectrum of $L_3Co(III)Na(I)$ in MeCN-d_3, showing the presence of one species 16

Figure S20. Mass spectrum (MALDI-TOF) of $L_3Co(III)Na(I)$, showing the presence of $[L_3Co(II)Na]^+$ 16

Figure S21. ^1H NMR spectrum of the proligand (bottom) and $L_3Co(III)Na(I)$ (top) 17

Figure S22. ^1H NMR spectrum of $L_4Co(III)Na(I)$ 17

Figure S23. $^{13}C\{^1H\}$ NMR spectrum of $L_4Co(III)Na(I)$ 18

Figure S24. ^1H-^1H COSY NMR spectrum of $L_4Co(III)Na(I)$ 18

Figure S25. ^1H -^{13}C HSQC NMR spectrum of $L_4Co(III)Na(I)$ 19

Figure S26. ^1H-^{13}C HMBC NMR spectrum of $L_4Co(III)Na(I)$ 19

Figure S27. Representative ^1H NMR spectrum of an aliquot of a PO/CO_2 ROCOP polymerization, showing peaks corresponding to polycarbonate (PPC) and cyclic carbonate (PC) formation 20

Figure S28. GPC trace of polycarbonate produced from the ROCOP of PO and CO_2 using $L_1Co(III)Na(I)$ (50 °C, 20 bar CO_2) 20

Figure S29. GPC trace of polycarbonate produced from the ROCOP of PO and CO_2 using $L_2Co(III)Na(I)$ (50 °C, 20 bar CO_2) 21

Figure S30. GPC trace of polycarbonate produced from the ROCOP of PO and CO_2 using $L_3Co(III)Na(I)$ (50 °C, 20 bar CO_2) 21

Figure S31. GPC trace of polycarbonate produced from the ROCOP of PO and CO_2 using L_1Co(III)Na(I) (70 °C, 20 bar CO_2) 22

Figure S32. GPC trace of polycarbonate produced from the ROCOP of PO and CO_2 using L_2Co(III)Na(I) (70 °C, 20 bar CO_2) 22

Figure S33. GPC trace of polycarbonate produced from the ROCOP of PO and CO_2 using L_1Co(III)Na(I) (50 °C, 20 bar CO_2, unpurified PO) 23

Figure S34. GPC trace of polycarbonate produced from the ROCOP of PO and CO_2 using L_2Co(III)Na(I) (50 °C, 20 bar CO_2, unpurified PO) 23

Figure S35. GPC trace of polycarbonate produced from the ROCOP of PO and CO_2 using L_3Co(III)Na(I) (50 °C, 20 bar CO_2, unpurified PO) 24

Figure S36. Crystal structure of L_2Co(III)Na(I). Selected hydrogen atoms are omitted for clarity. Thermal ellipsoids are represented at 40% probability. 29

Figure S37. Polymeric crystal structure of L_2Co(III)Na(I), showing an extra repeat unit. Selected hydrogen atoms are omitted for clarity. Thermal ellipsoids are represented at 40 % probability. 29

Figure S38. Crystal structure of L_3Co(III)Na(I). Selected hydrogen atoms are omitted for clarity. Thermal ellipsoids are represented at 40% probability. 30

Figure S39. Dimeric structure of L_3Co(III)Na(I). Selected hydrogen atoms are omitted for clarity. Thermal ellipsoids are represented at 40% probability. 30

Figure S40. ^{1}H NMR spectrum of L_2Co(III)Na(I) dissolved in MeCN-d_3 (top) and spectrum of L_2Co(III)Na(I) dissolved in PO (bottom) 35

Figure S41. Plot of k_{obs} for L_nCo(III)Na(I) (n = 1 – 4) versus buried volume (V_{Bur}) around Co(III) 38

Figure S42. Topographical steric maps of L_nCo(III)Na(I) (n = 1 – 4) viewed along the positive z-direction 38

Catalyst Characterization:

$L_1Co(III)Na(I)$ and $L_4Co(III)Na(I)$ are previously reported compounds, synthesised according to literature procedures.[1, 2] $L_2Co(III)Na(I)$ and $L_3Co(III)Na(I)$ are novel compounds, and are fully characterized in this work. See main manuscript for general procedures and experimental methods.

$L_1Co(III)Na(I)$ catalyst characterization:

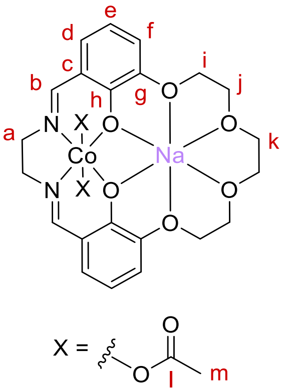

73% yield; ^{1}H NMR (500 MHz, $CDCl_3$, 298 K) δ (ppm): 7.76 (s, 2H, N=C**H** (**b**)), 6.89 (d, 2H, $^3J_{H-H}$ = 7.96 Hz, *meta*-Ar**H** (**d**)), 6.79 (d, 2H, $^3J_{H-H}$ = 7.99 Hz, meta-Ar**H** (**f**)), 6.45 (t, 2H, $^3J_{H-H}$ = 7.88 Hz, para-Ar**H** (**e**)), 4.35 (s, 4H, NC**H₂** (**a**)), 4.20 (m, 4H, C**H₂** (**i** or **j**)), 3.95 (m, 4H, C**H₂** (**i** or **j**)), 3.86 (s, 4H, C**H₂** (**k**)), 1.43 (s, 6H, O=C-C**H₃** (**m**)); ^{13}C {^{1}H} NMR (151 MHz, 298 K, $CDCl_3$) δ (ppm): 179.6 (s, O=**C** (**l**)), 164.8 (s, N=**C**H (**b**)), 156.8 (s, *ipso*-**C** (**h**)), 152.0 (s, *ortho*-**C** (**g**)), 126.4 (s, *meta*-**C** (**d**)), 119.3 (s, *ortho*-C (**c**)), 114.9 (s, *meta*-C (**f**)), 112.8 (s, *para*-**C** (**e**)), 69.1 (s, O-**C**H₂ (**i** or **j** or **k**)), 69.7 (s, O-**C**H₂ (**i** or **j** or **k**)), 67.2 (s, O-**C**H₂ (**i** or **j** or **k**)), 58.9 (s, N-**C**H₂ (**a**)), 24.8 (s, O=C-**C**H₃ (**m**)). Anal. Calc. for $C_{26}H_{30}CoN_2NaO_{10}$: C 50.99, H 4.94, N 4.57; Found: C 51.20, H 5.10, N 4.15 %.

$L_2Co(III)Na(I)$ catalyst characterization:

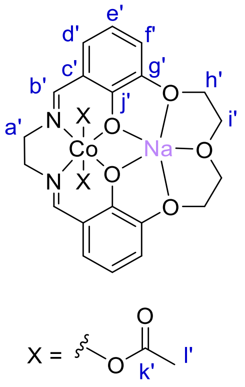

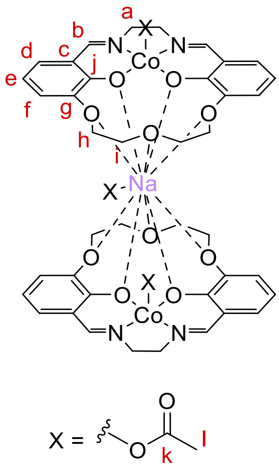

Unusual product speciation precludes straightforward yield calculation; ^{1}H NMR (500 MHz, MeCN-d_3, 298 K) δ (ppm): 7.94 (s, 2H, N=C**H** (**b**)), 7.92 (s, 2H, N=C**H** (**b'**)), 7.13-7.07 (m, 4H, *meta*-CH (**d'** and **f'**)), 7.05-7.00 (m, 4H, *meta*-CH (**d** and **f**), 6.53-6.47 (m, 4H, *para*-C**H** (**e** and **e'**)), 4.40 (m, 4H, C**H₂** (**i** or **h**)), 4.31 (s, 4H, N-C**H₂** (**a'**)), 4.27 (m, 4H, C**H₂** (**i'** or **h'**)), 4.16 (s, 4H, N-C**H₂** (**a**)), 4.11 (m, 4H, C**H₂** (**i** or **h**)), 3.64 (m, 4H, C**H₂** (**i** or **h**)), 1.29 (s, O=C-C**H₃** (**l** or **l'**)), 1.26 (s, O=C-C**H₃** (**l** or **l'**)); ^{13}C{^{1}H} NMR (151 MHz, 298 K, MeCN-d_3) δ (ppm): 178.60 (s, O=C (**k** and **k'**)), 166.43 (s, N=CH (**b** or **b'**)), 165.95 (s, N=**C**H (**b** or **b'**)), 161.04 (s, *ispo*-**C** (**j** or **j'**)), 158.96 (s, *ipso*-**C** (**j** or **j'**)), 153.75 (s, *ortho*-**C** (**g** or **g'**)), 152.56 (s, *ortho*-**C** (**g** or **g'**)), 130.29 (s, *meta*-CH (**d'** or **f'**)), 128.48 (s, *meta*-**C**H (**d**

or **f**), 125.86 (s, *meta*-**C**H (**d'** or **f'**)), 122.18 (s, *ortho*-**C** (**c** or **c'**)), 121.61 (s, *ortho*-**C** (**c** or **c'**)), 119.78 (s, *meta*-**C**H (**d** or **f**)), 114.21 (s, *para*-**C**H (**e** or **e'**)), 114.09 (s, *para*-**C**H (**e** or **e'**)), 77.31 (s, O-**C**H$_2$ (**h'** or **i'**), 70.56 (s, O-**C**H$_2$ (**h'** or **i'**)), 70.39 (s, O-**C**H$_2$ (**h** or **i**)), 69.92 (s, O-**C**H$_2$ (**h** or **i**)), 59.61 (s, N-**C**H$_2$ (**a** or **a'**)), 59.50 (s, N-**C**H$_2$ (**a** or **a'**)), 24.93 (s, O=C-**C**H$_3$ (**l** and **l'**)); Molecular cation (MALDI-TOF): 450.1 amu, $[L_2Co(II)Na]^+$; 877.0 amu, $[(L_2)_2Co(II)_2Na]^+$. Anal. Calc. for formula unit $C_{24}H_{26}CoN_2NaO_9$; C 50.71, H 4.61, N 4.93; Found: C 50.16, H 4.46, N 5.29 %.

L_3Co(III)Na(I) catalyst characterization:

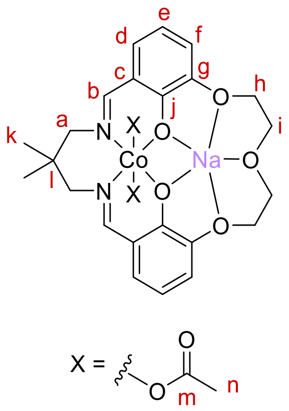

63% yield; ^{1}H NMR (500 MHz, CDCl$_3$, 298 K) δ (ppm): 7.16 (s, 2H, N=C**H** (**b**)), 6.69 (d, 2H, $^3J_{H\text{-}H}$ = 8.3 Hz, *meta*-CH (**d**)), 6.63 (d, 2H, $^3J_{H\text{-}H}$ = 7.5 Hz, *meta*-C**H** (**f**)), 6.39 (t, 2H, $^3J_{H\text{-}H}$ = 7.8 Hz, *para*-C**H** (**e**)), 4.21 (m, 4H, C**H$_2$** (**h** or **i**)), 3.89 (m, 4H, C**H$_2$** (**h** or **i**)), 3.58 (s, 4H, N-C**H$_2$** (**a**)), 1.45 (s, 6H, O=C-C**H$_3$** (**n**)), 1.18 (s, 6H, C-C**H$_3$** (**k**)); ^{13}C {^{1}H} NMR (151 MHz, 298 K, CDCl$_3$) δ (ppm): 178.8 (s, O=C (**m**)), 164.3 (s, N=**C**H (**b**)), 157.3 (s, *ipso*-**C** (**j**)), 151.7 (s, *ortho*-**C** (**g**)), 123.5 (s, *meta*-CH (**d**)), 121.6 (s, *ortho*-**C**-CH=N (**c**)), 114.3 (s, *para*-CH (**e**)), 112.5 (s, *meta*-CH (**f**)), 71.4 (s, N-**C**H$_2$ (**a**)), 67.4 (s, O-CH$_2$ (**h** or **i**)), 33.8 (s, C-**C**(CH$_3$)$_2$ (**l**)), 23.8 (s, C-C(**C**H$_3$)$_2$ (**k**)), 23.5 (s, O=C-CH$_3$ (**n**)); Molecular cation (MALDI-TOF): 492.1 amu, $[L_3Co(II)Na]^+$. Anal. Calc. for $C_{27}H_{32}CoN_2NaO_9$: C 53.12, H 4.96, N 4.59; Found: C 52.58, H 4.96, N 4.65 %.

L_4Co(III)Na(I) catalyst characterization:

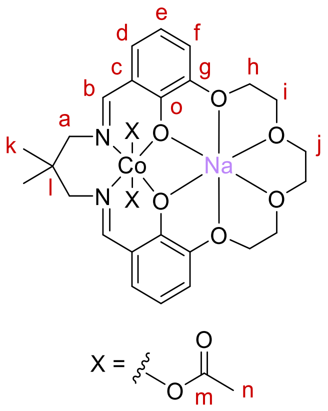

13% yield; ^{1}H NMR (500 MHz, MeCN-d$_3$, 298 K) δ (ppm): 7.29 (s, 2H, N=C**H** (**b**)), 6.79 – 6.69 (m, 4H, *meta*-C**H** (**d** and **f**)), 6.35 (t, 2H, $^3J_{H\text{-}H}$ = 7.9 Hz, *para*-C**H** (**e**)), 4.05 (m, 4H, C**H$_2$** (**h** or **i**)), 3.82 (m, 4H, C**H$_2$** (**h** or **i**)), 3.75 (s, 4H, O-C**H$_2$** (**j** or **a**)), 3.42 (s, 4H, O-C**H$_2$** (**j** or **a**)), 1.24-1.20 (m, 6H, O=C-C**H$_3$** (**n**)), 1.09 (s, 6H, C-C**H$_3$** (**k**)). ^{13}C {^{1}H} NMR (151 MHz, 298 K, CDCl$_3$) δ (ppm): 167.2 (s, N=**C**H (**b**)), 158.5 (s, *ipso*-**C** (**o**)), 142.4 (s, *ortho*-**C** (**g**)), 126.7 (s, *meta*-**C**H (**d** or **f**)), 123.3 (s, *ortho*-**C** (**c**)),

117.6 (s, *meta*-**C**H (**d** or **f**)), 114.1 (s, *para*-**C**H (**e**)), 71.1 – 69.2 (O-**C**H_2, N-**C**H_2 (**a**, **h**, **i** and **j**)), 35.6 (s, C-**C**$(CH_3)_2$ (**l**)), 25.1 (C-C(**C**H_3)$_2$, O=C-**C**H_3 (**n** or **k**)), 24.8 (C-C(**C**H_3)$_2$, O=C-**C**H_3 (**n** or **k**)). Anal. Calc. for $C_{29}H_{36}CoN_2NaO_{10}$: C 53.22, H 5.54, N 4.28; Found: C 52.87, H 5.68, N 3.71 %.

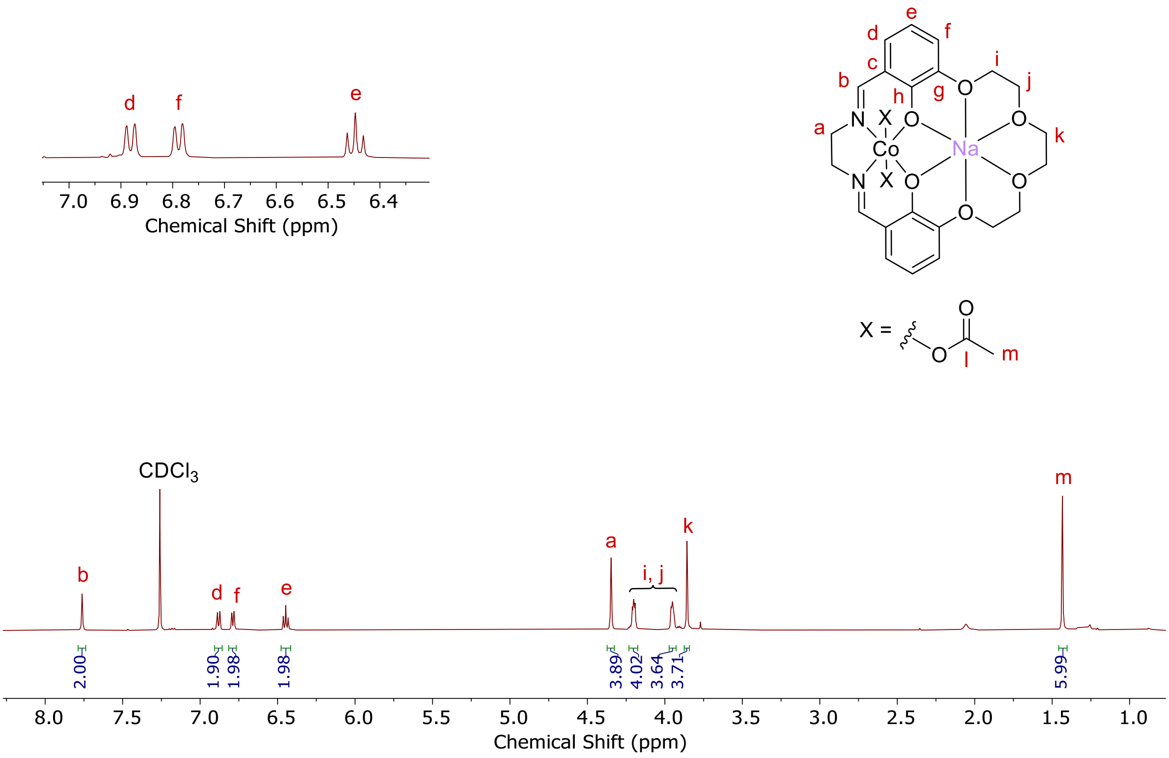

Figure S1. ^{1}H NMR spectrum of $L_1Co(III)Na(I)$

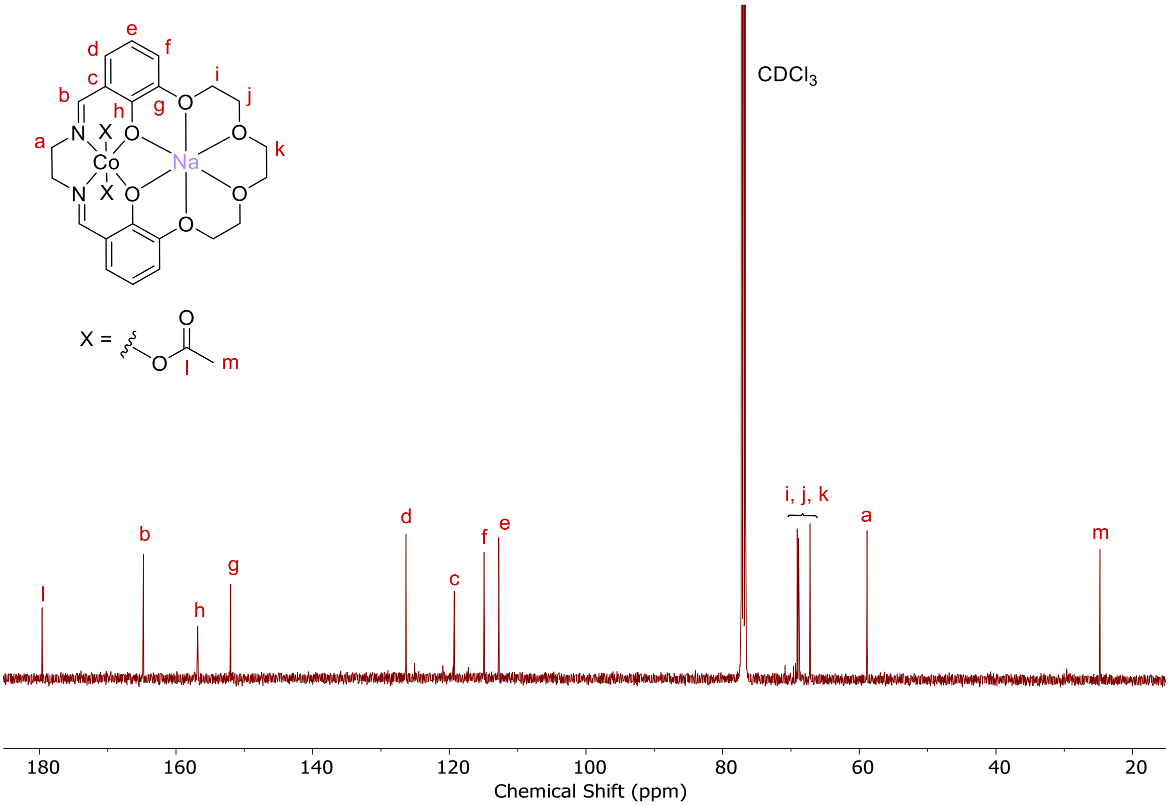

Figure S2. $^{13}C\{^{1}H\}$ NMR spectrum of $L_1Co(III)Na(I)$

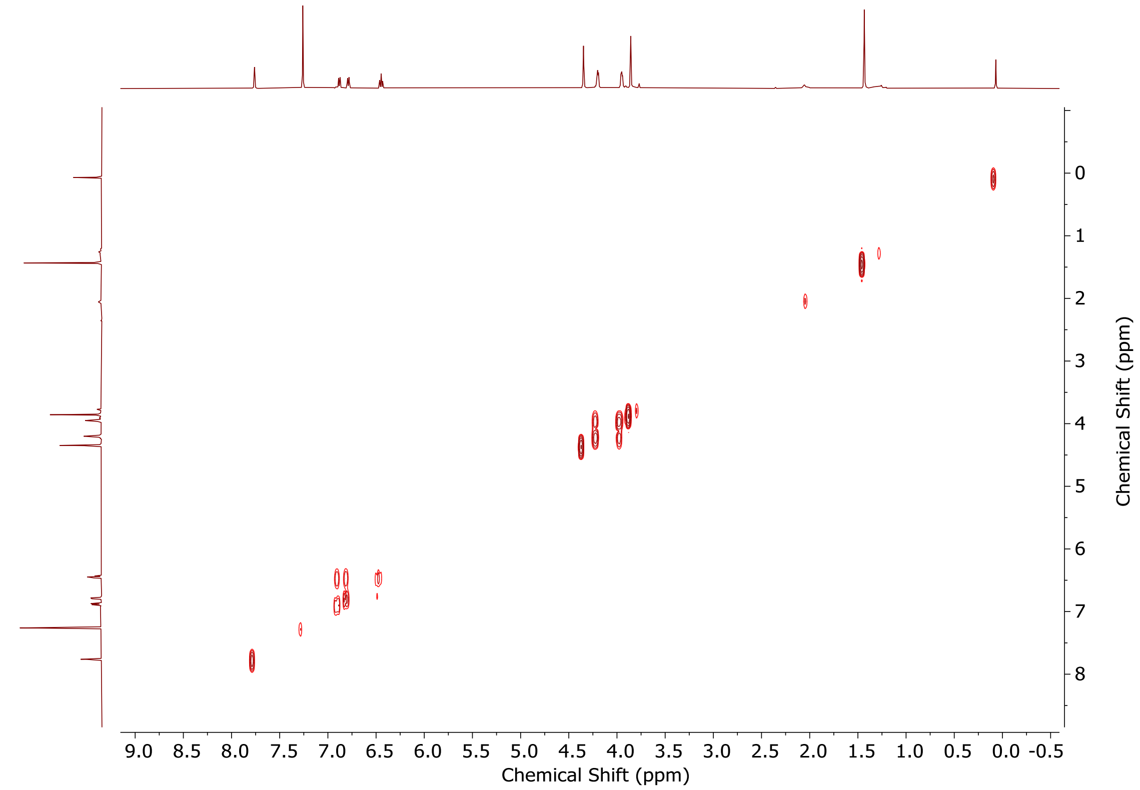

Figure S3. ^{1}H-^{1}H COSY NMR spectrum of $L_1Co(III)Na(I)$

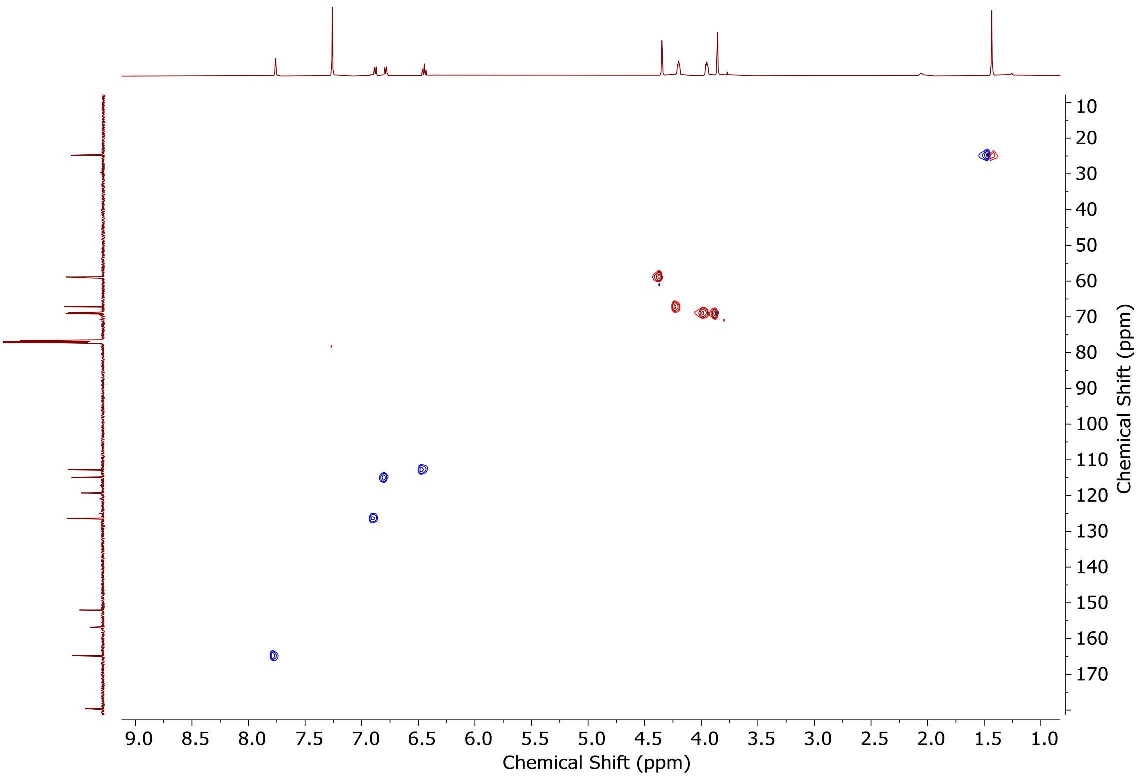

Figure S4. ^{1}H-^{13}C HSQC NMR spectrum of $L_1Co(III)Na(I)$

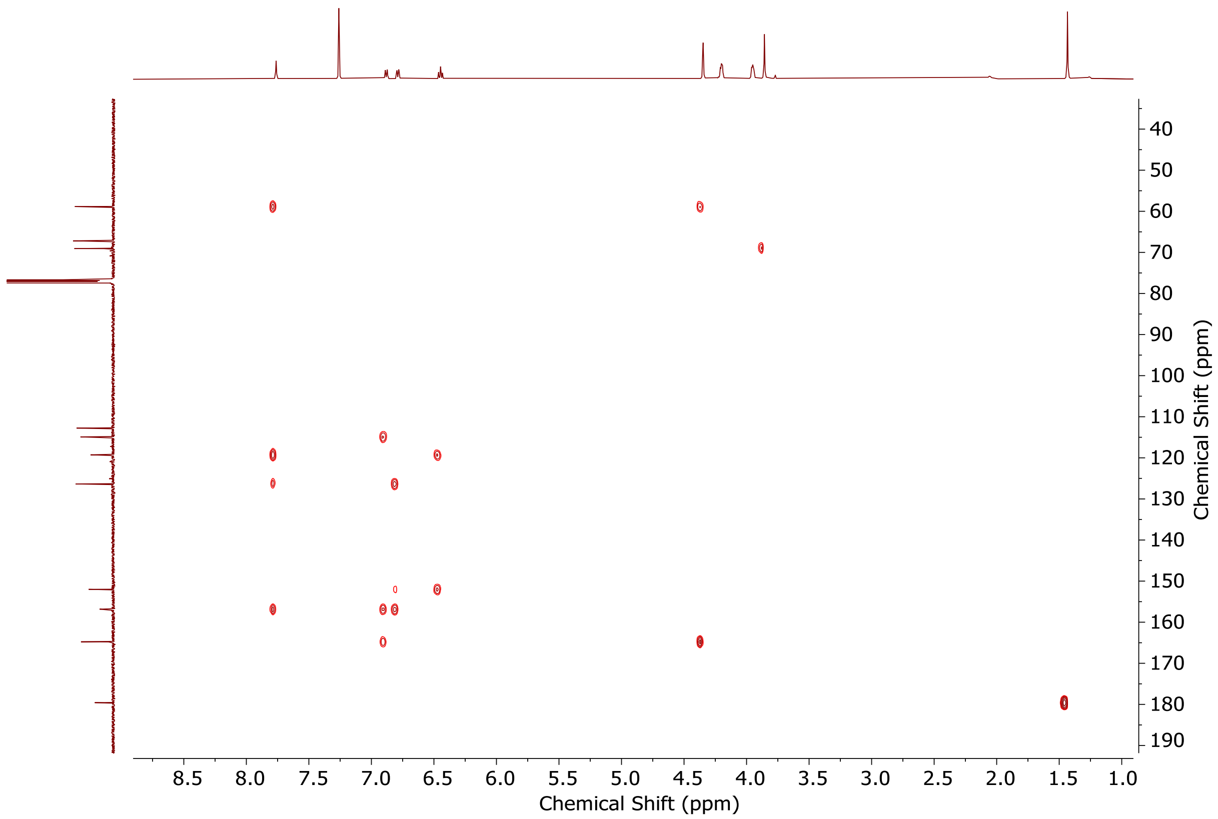

Figure S5. ^{1}H-^{13}C HMBC NMR spectrum of $L_1Co(III)Na(I)$

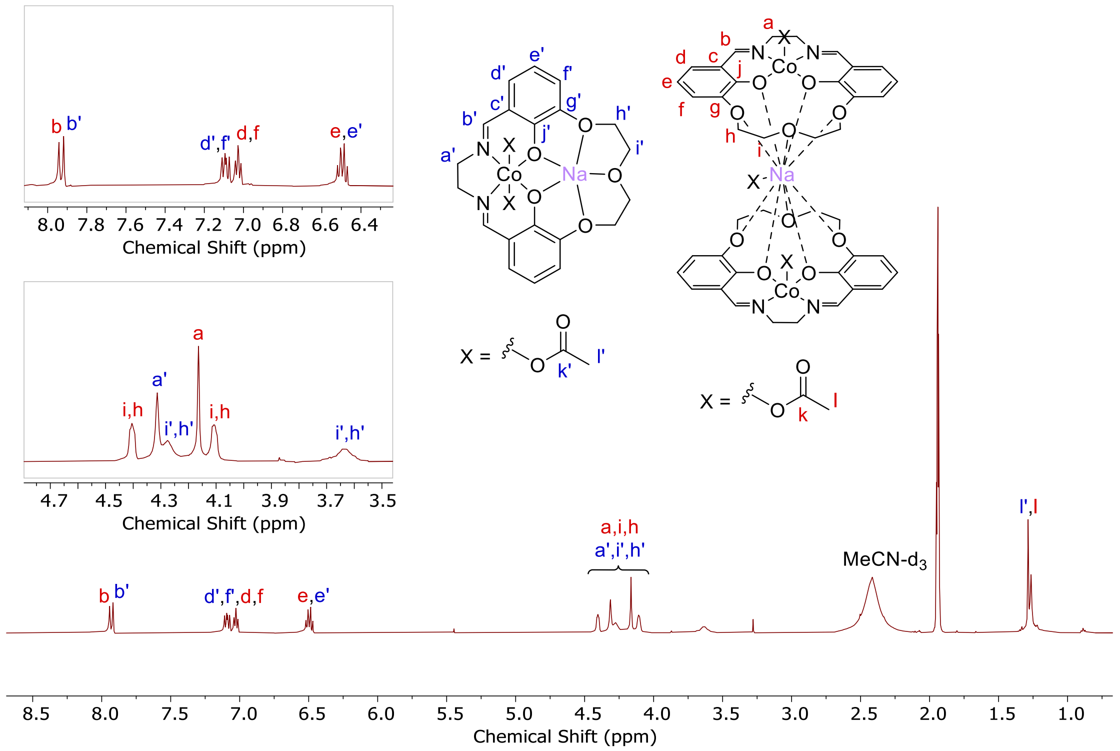

Figure S6. ^{1}H NMR spectrum of $L_2Co(III)Na(I)$, assigned according to the presence of two species

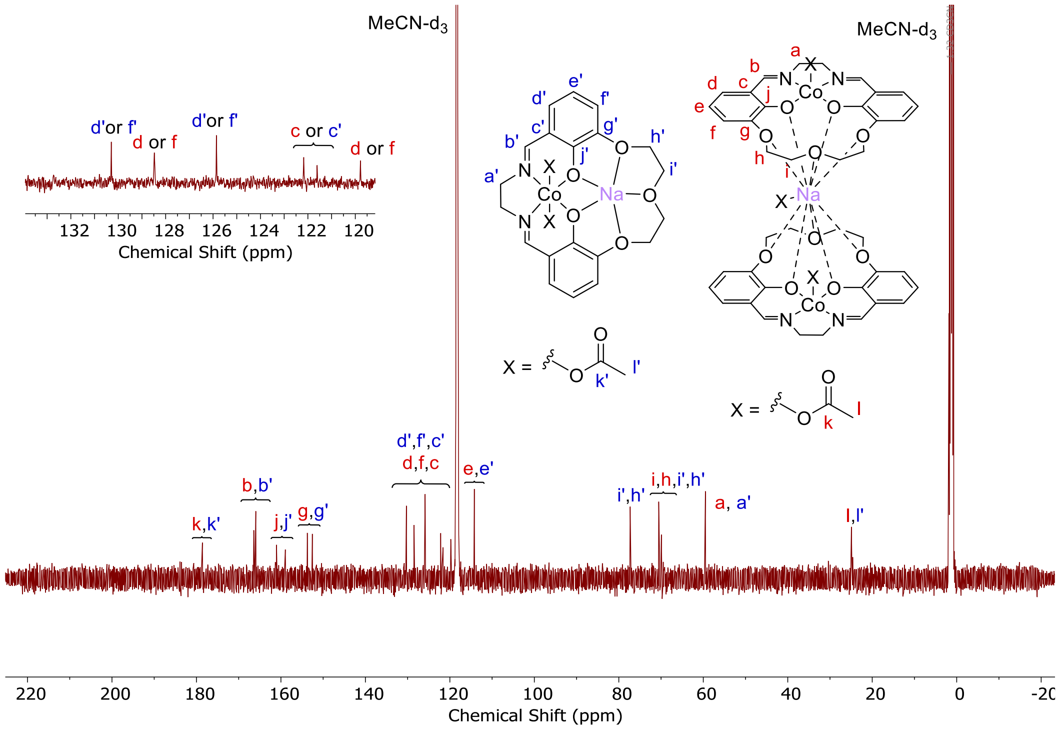

Figure S7. ${}^{13}C\{{}^{1}H\}$ NMR spectrum of $L_2Co(III)Na(I)$, assigned according to the presence of two species

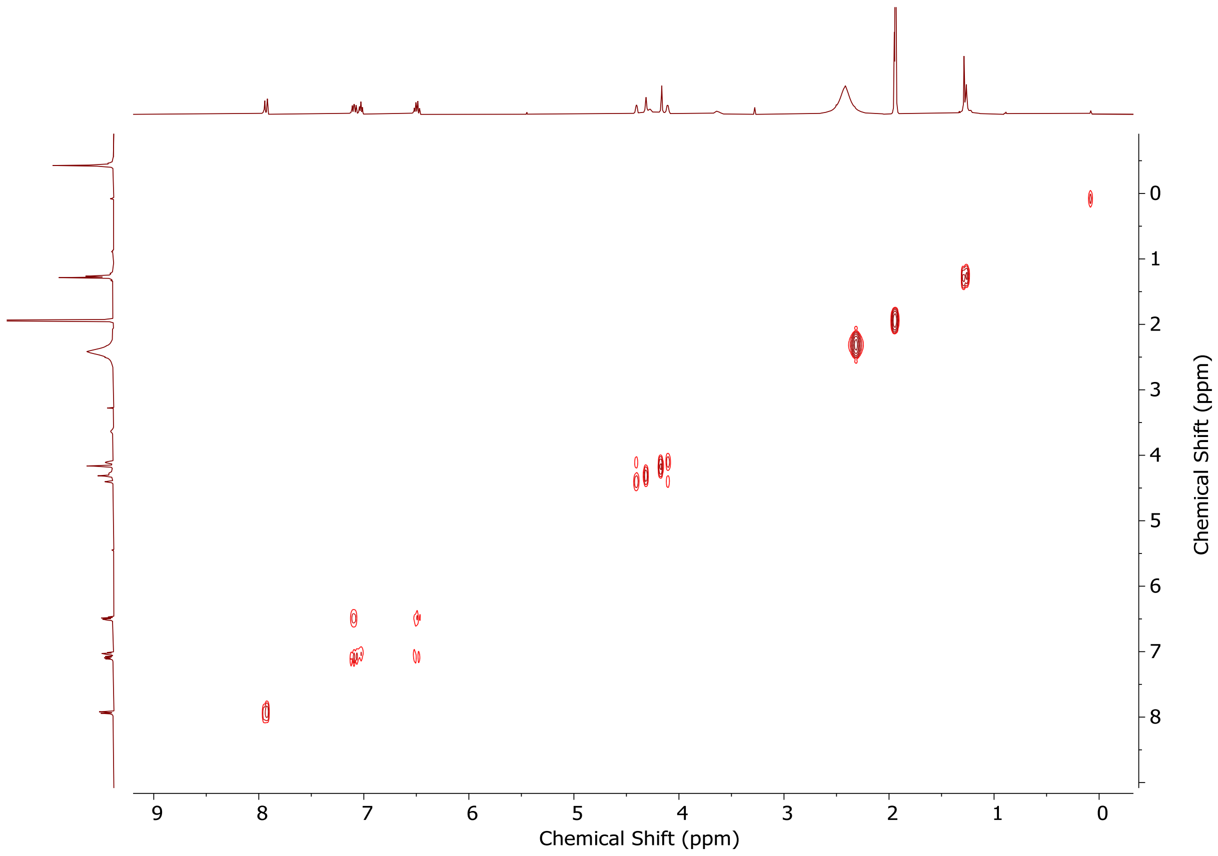

Figure S8. ${}^{1}H$ -${}^{1}H$ COSY NMR spectrum of $L_2Co(III)Na(I)$

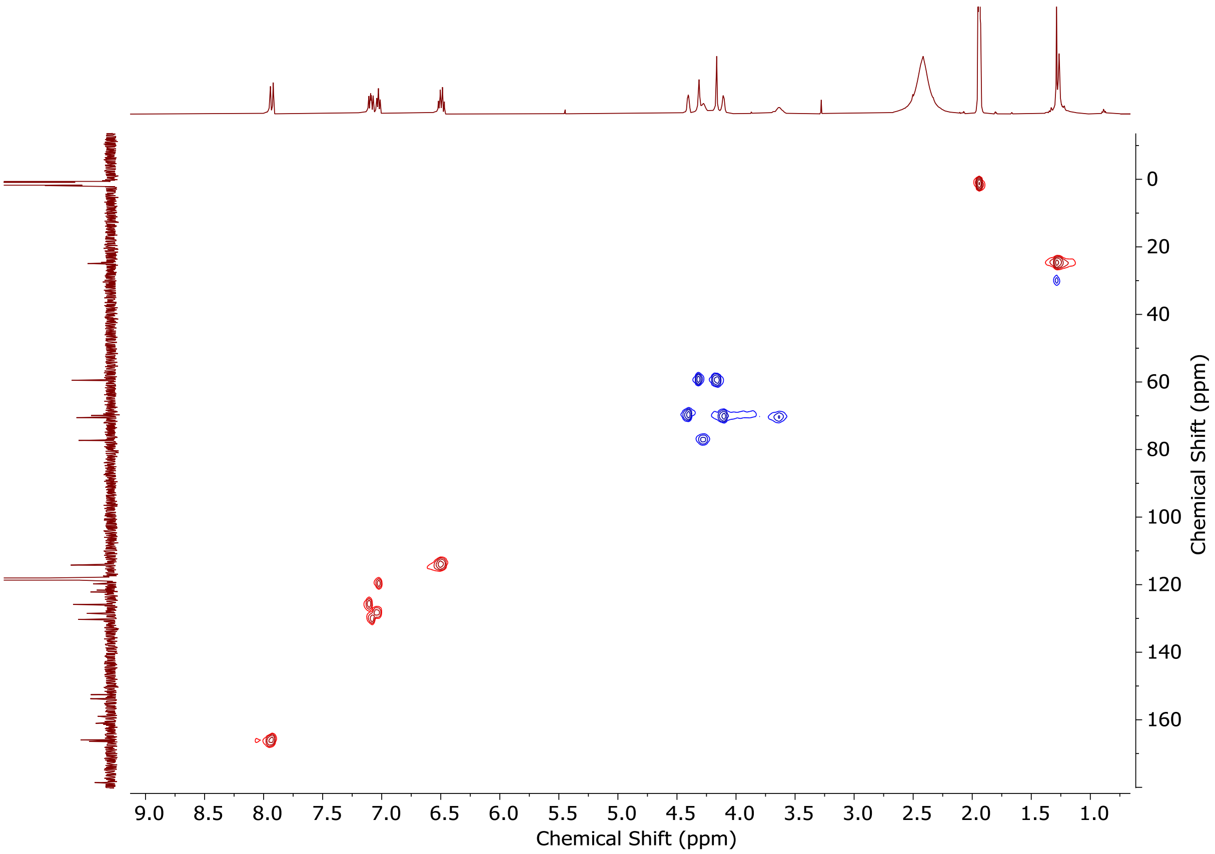

Figure S9. ^{1}H -^{13}C HSQC NMR spectrum of L_2Co(III)Na(I)

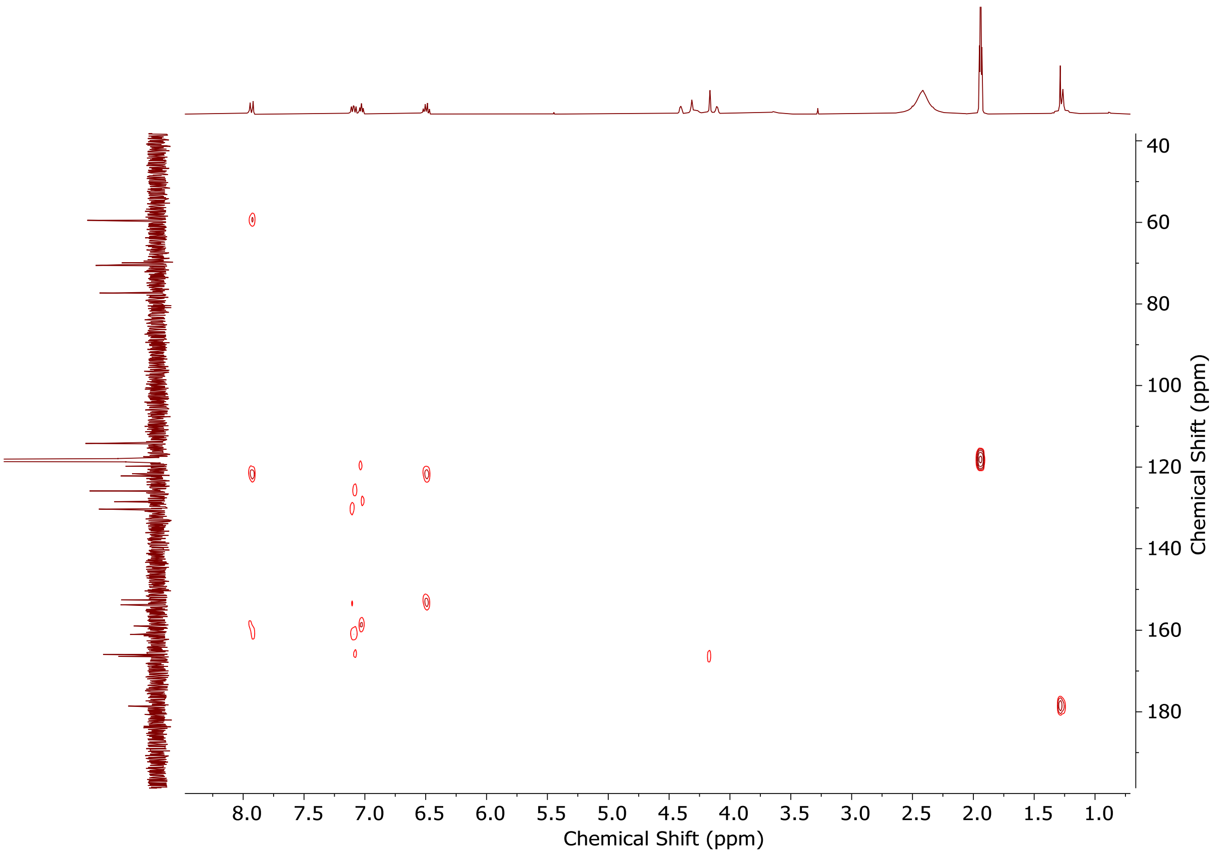

Figure S10. ^{1}H -^{13}C HMBC NMR spectrum of L_2Co(III)Na(I)

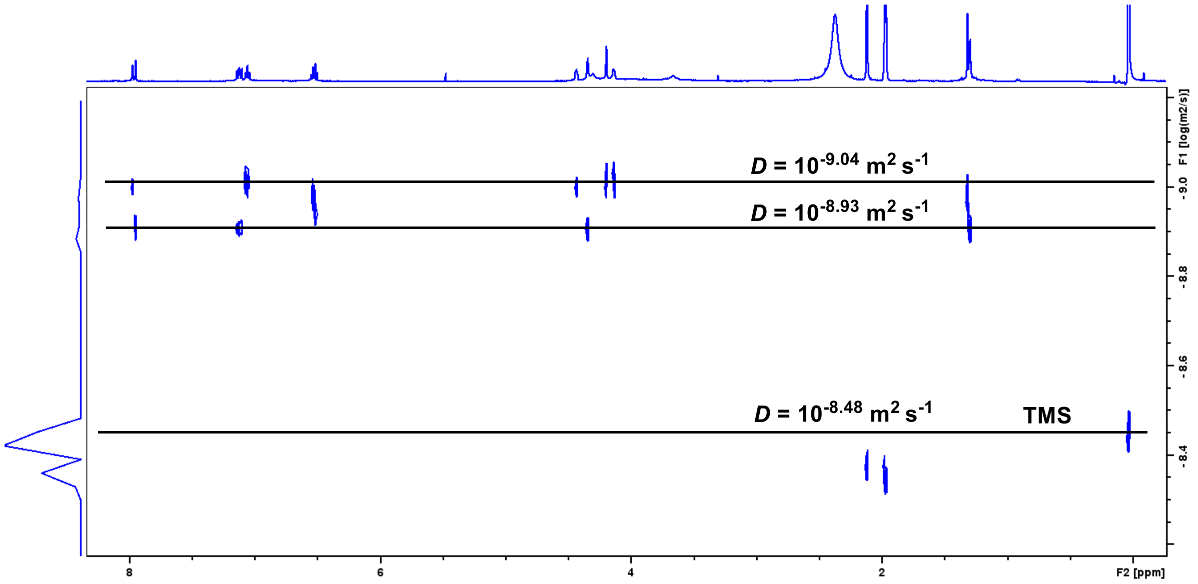

Figure S11. ^{1}H DOSY NMR spectrum of $L_2Co(III)Na(I)$ in MeCN-d_3, showing the presence of two species

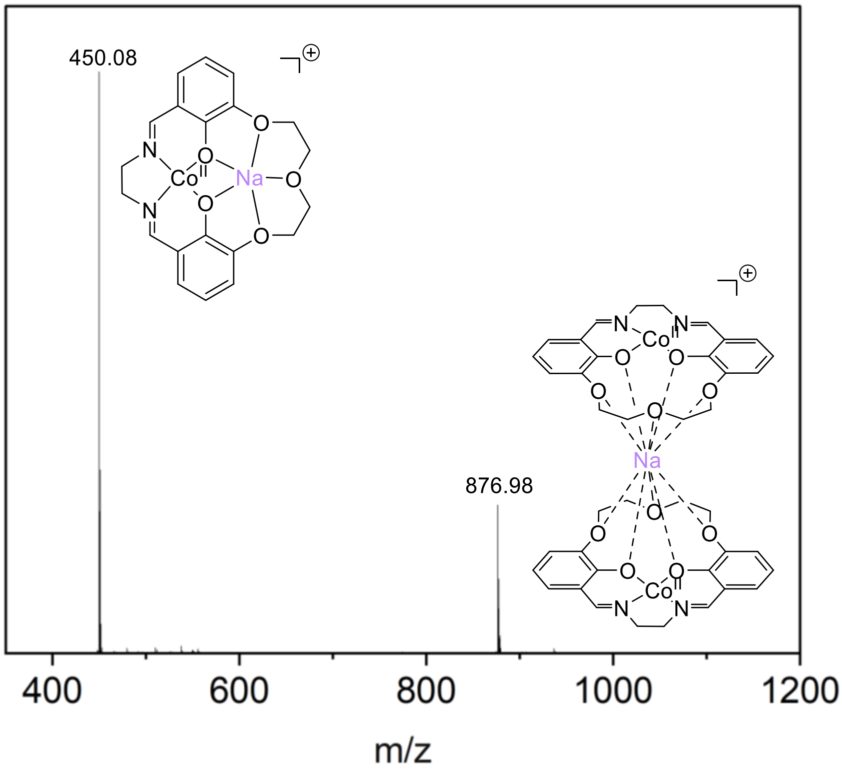

Figure S12. Mass spectrum (MALDI-TOF) of $L_2Co(III)Na(I)$, showing the presence of two species ($[L_2Co(II)Na(I)]^+$ and $[(L_2Co(II))_2Na(I)]^+$)

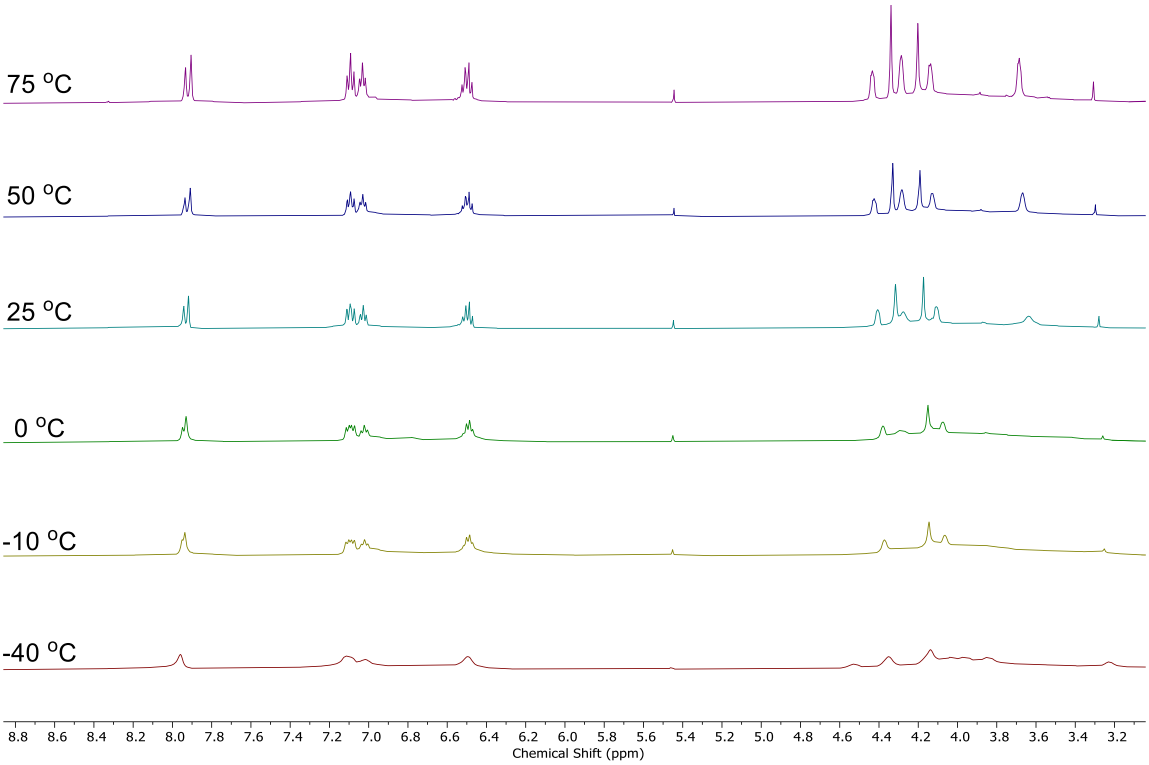

Figure S13. Variable Temperature NMR spectra of $L_2Co(III)Na(I)$ in MeCN-d_3

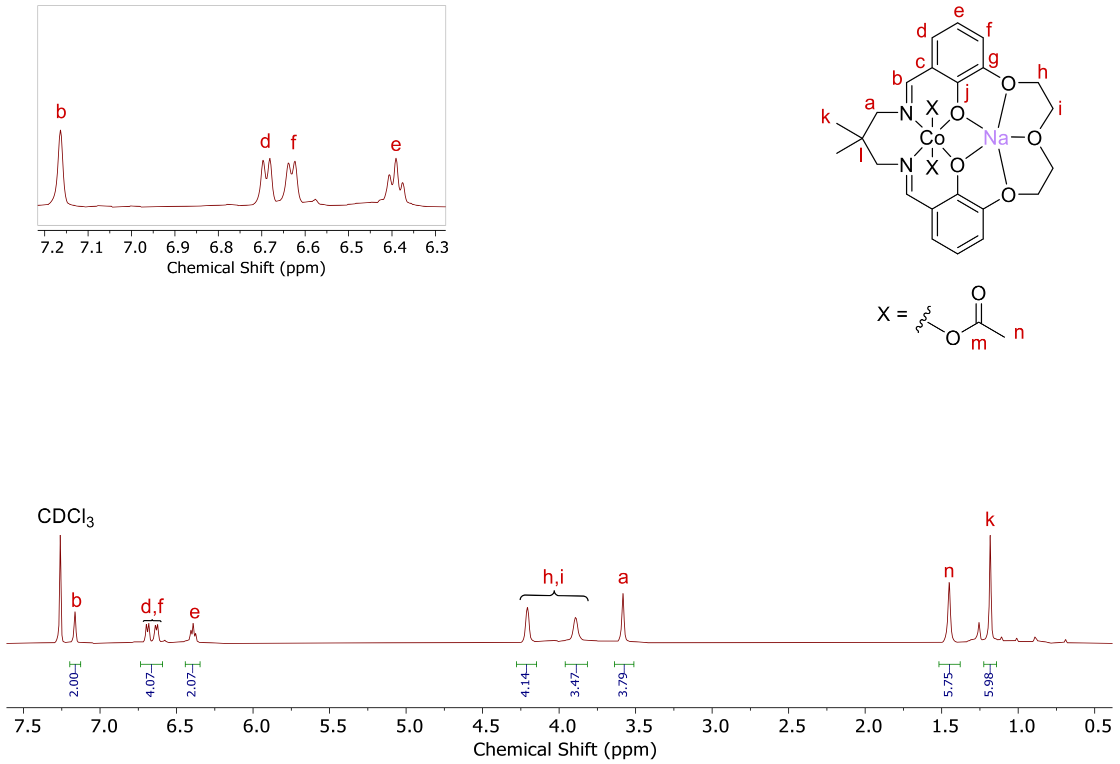

Figure S14. ${}^{1}H$ NMR spectrum of $L_3Co(III)Na(I)$

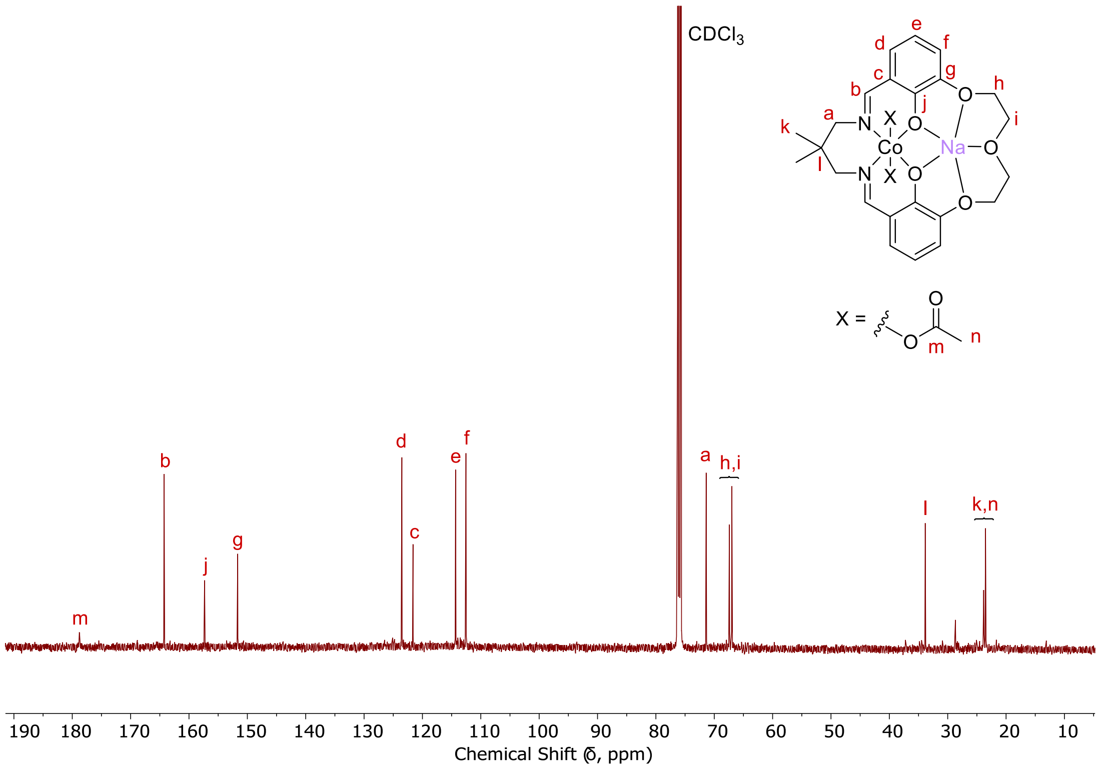

Figure S15. $^{13}C\{^{1}H\}$ NMR spectrum of $L_3Co(III)Na(I)$

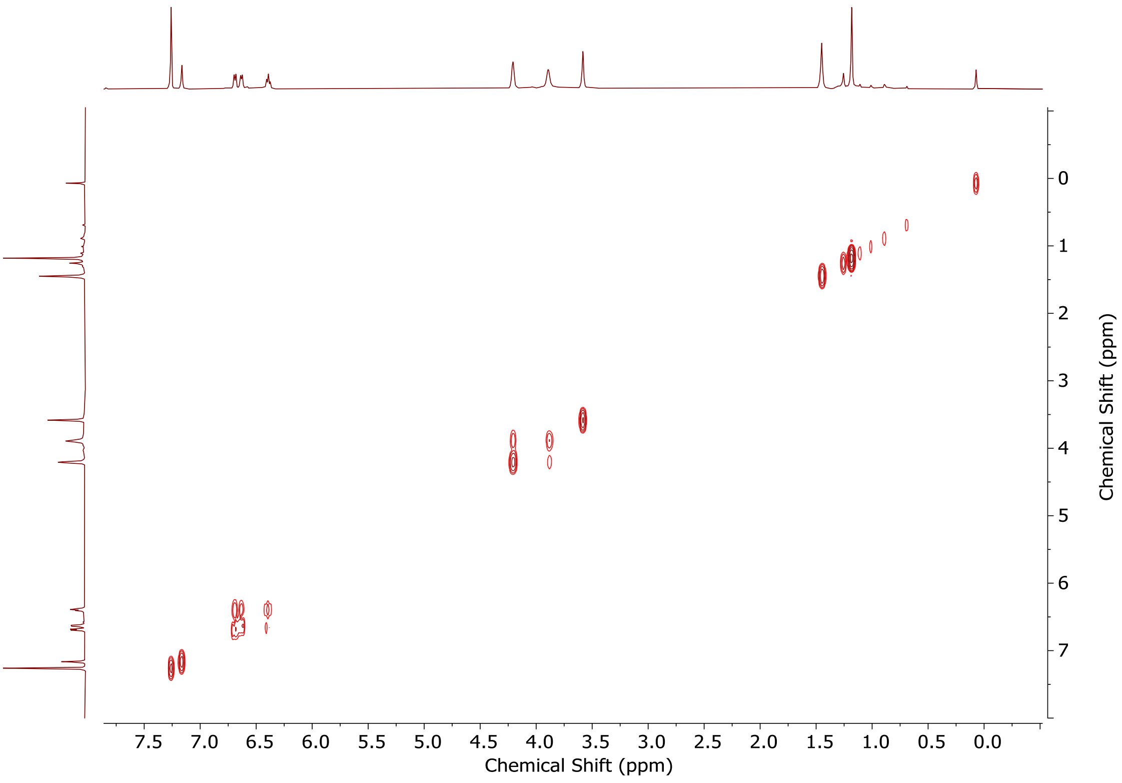

Figure S16. ^{1}H-^{1}H COSY NMR spectrum of $L_3Co(III)Na(I)$

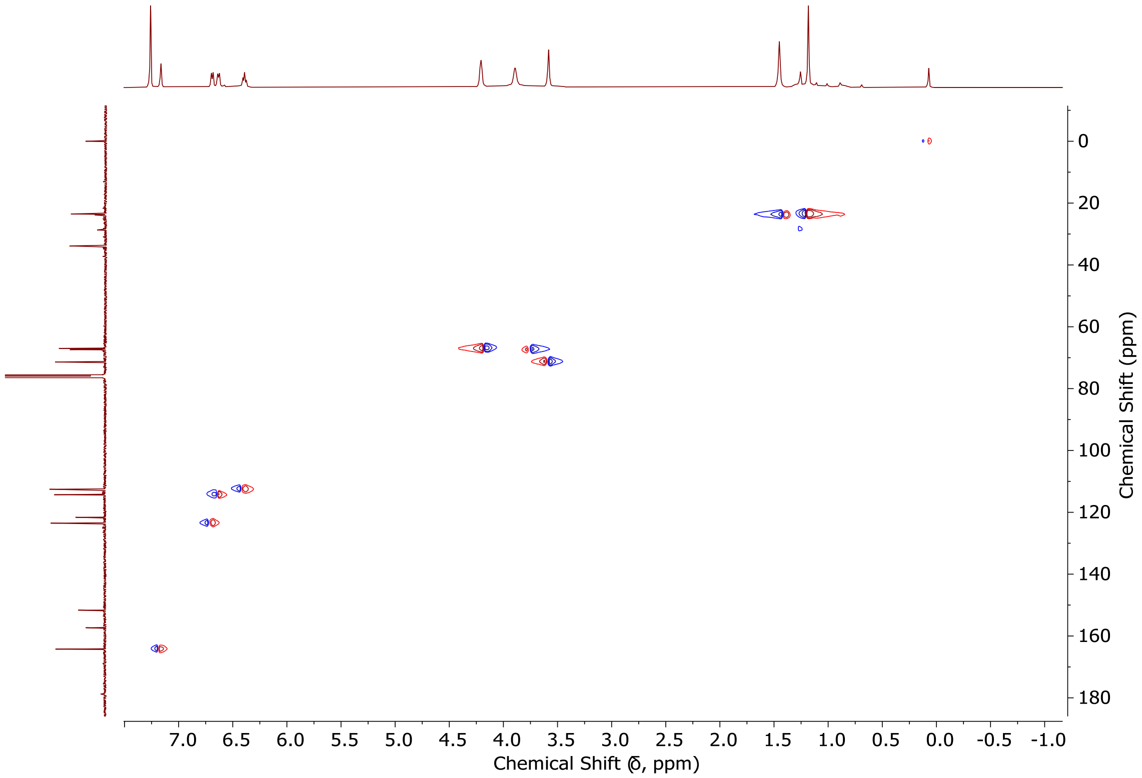

Figure S17. ^{1}H -^{13}C HSQC NMR spectrum of $L_3Co(III)Na(I)$

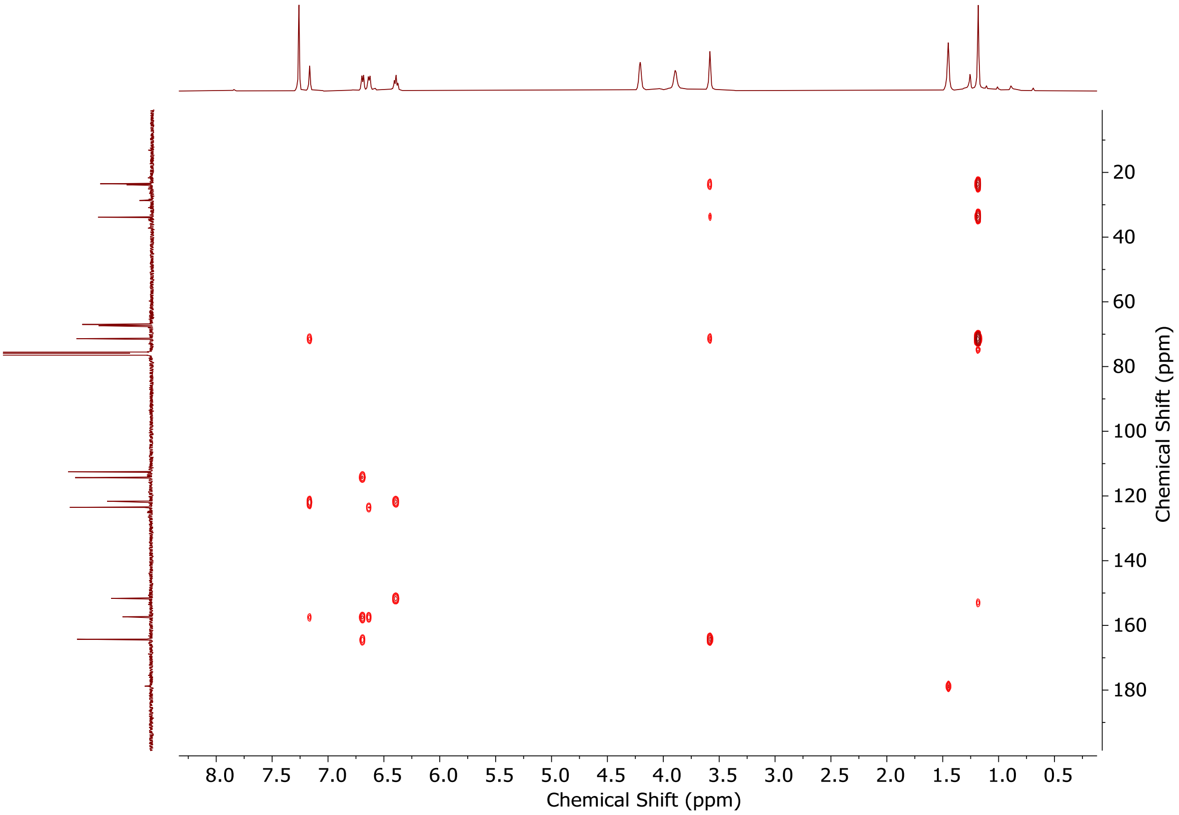

Figure S18. ^{1}H-^{13}C HMBC NMR spectrum of $L_3Co(III)Na(I)$

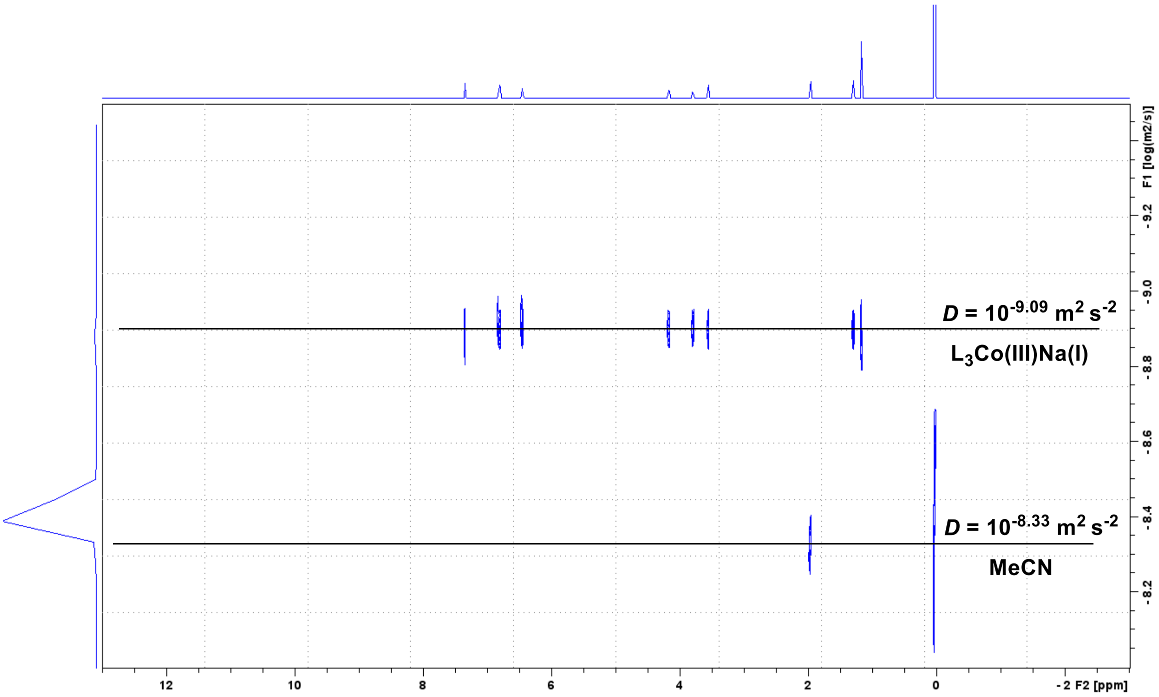

Figure S19. ^{1}H DOSY NMR spectrum of $L_3Co(III)Na(I)$ in MeCN-d_3, showing the presence of one species

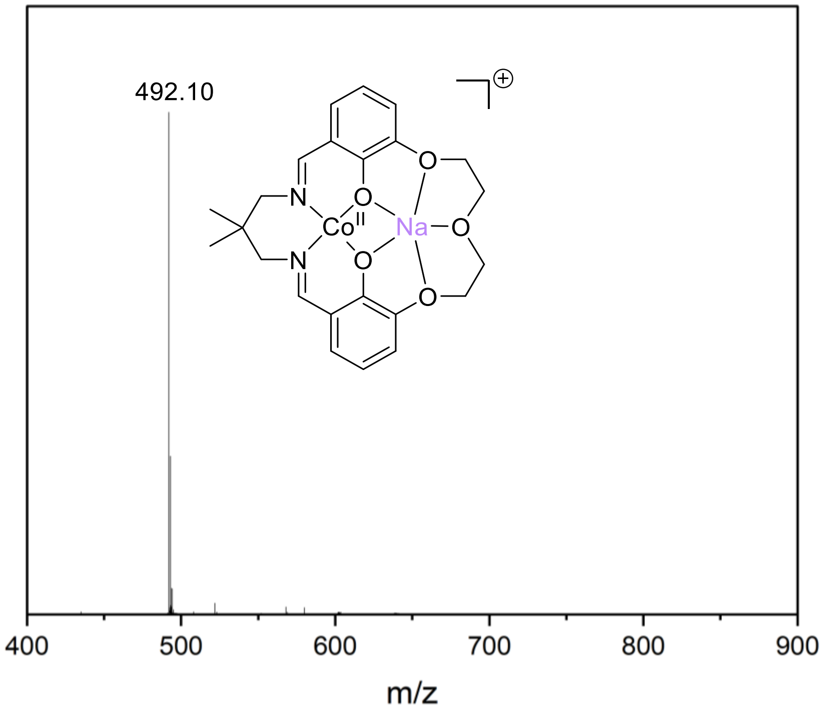

Figure S20. Mass spectrum (MALDI-TOF) of $L_3Co(III)Na(I)$, showing the presence of $[L_3Co(II)Na]^+$

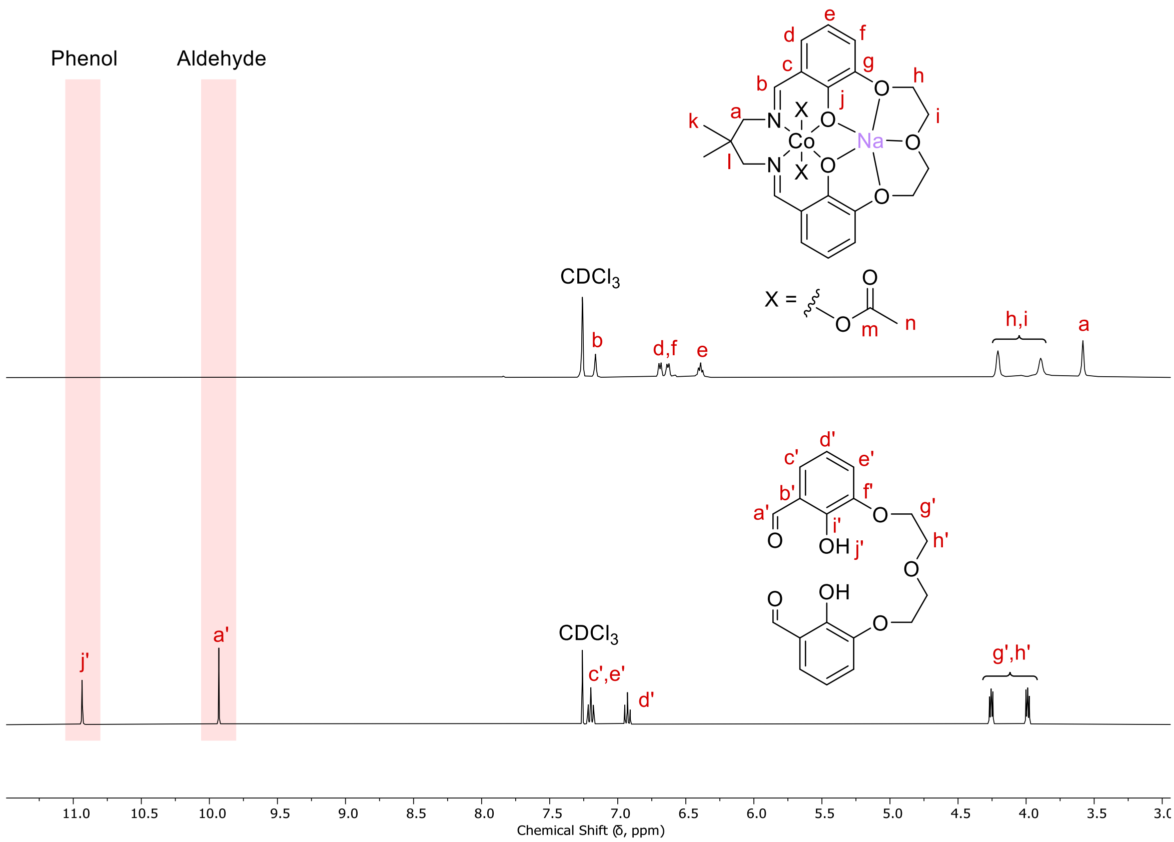

Figure S21. ^{1}H NMR spectrum of the proligand (bottom) and L_3Co(III)Na(I) (top)

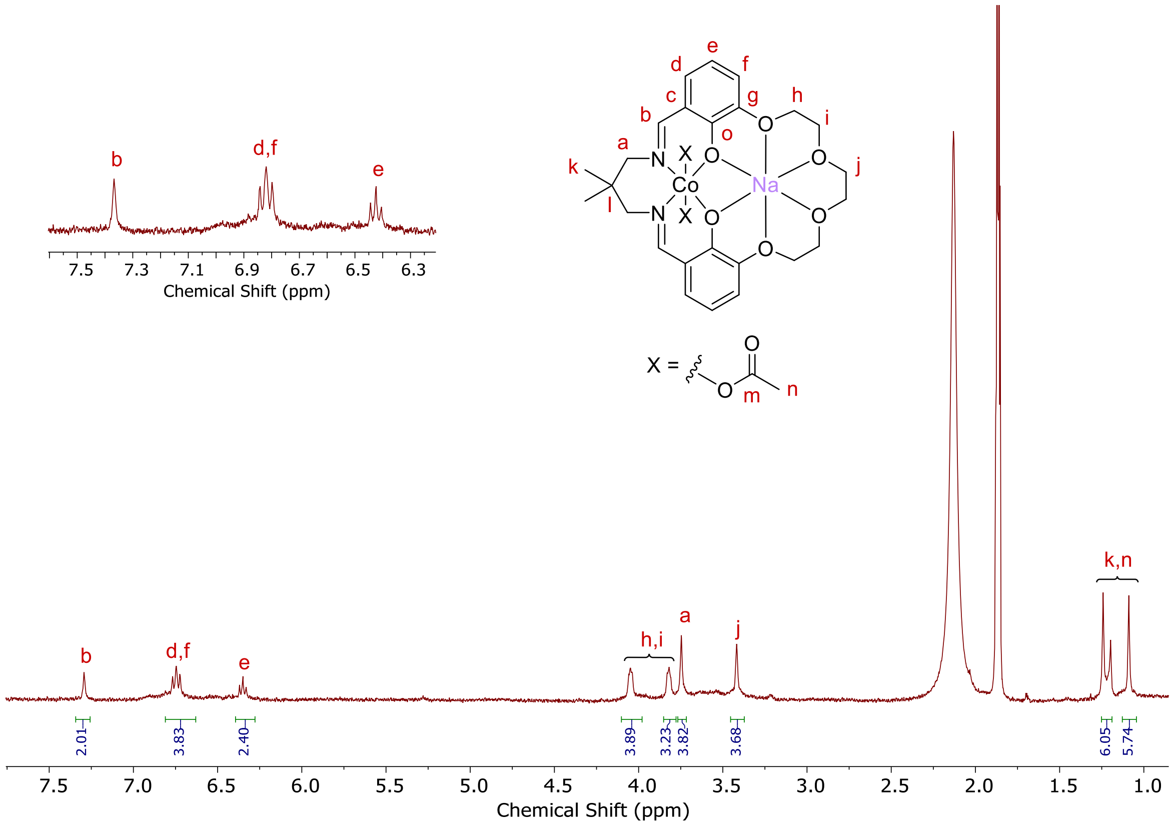

Figure S22. ^{1}H NMR spectrum of L_4Co(III)Na(I)

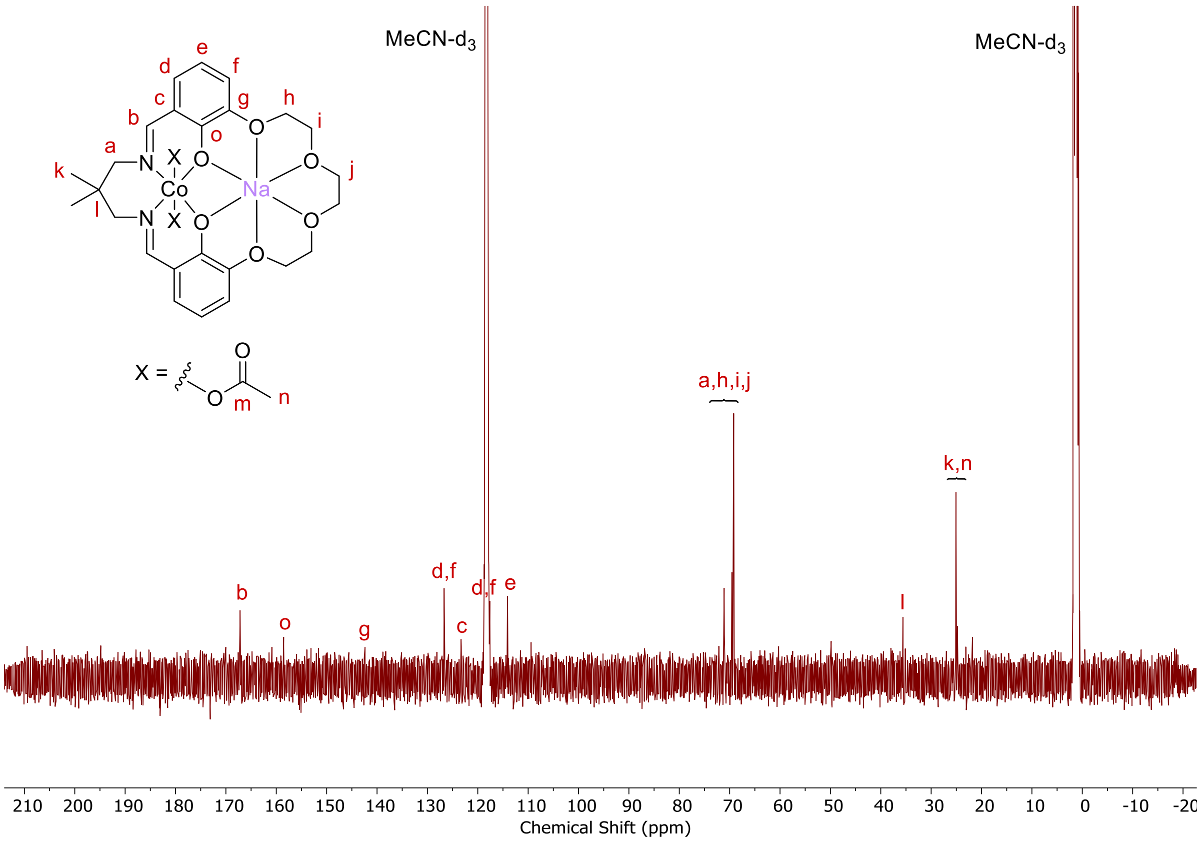

Figure S23. $^{13}C\{^{1}H\}$ NMR spectrum of $L_4Co(III)Na(I)$

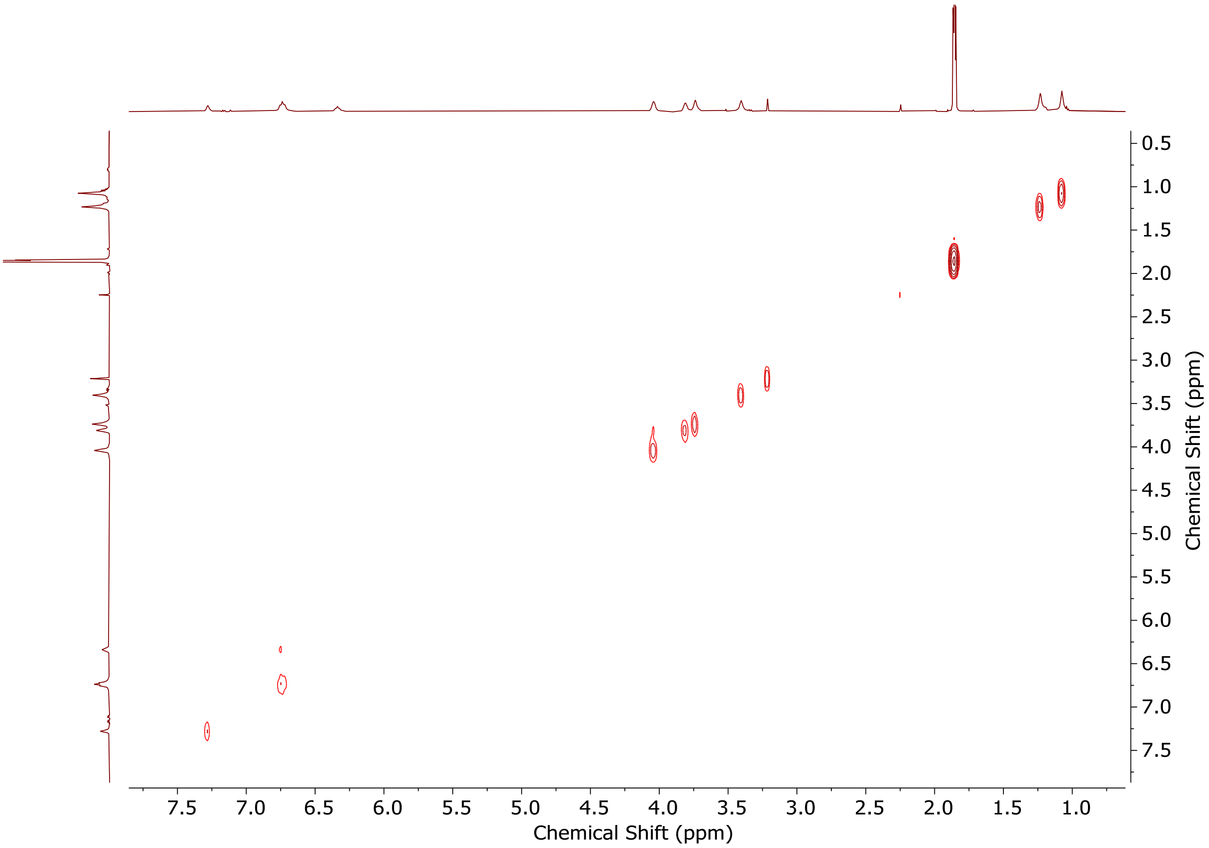

Figure S24. ^{1}H-^{1}H COSY NMR spectrum of $L_4Co(III)Na(I)$

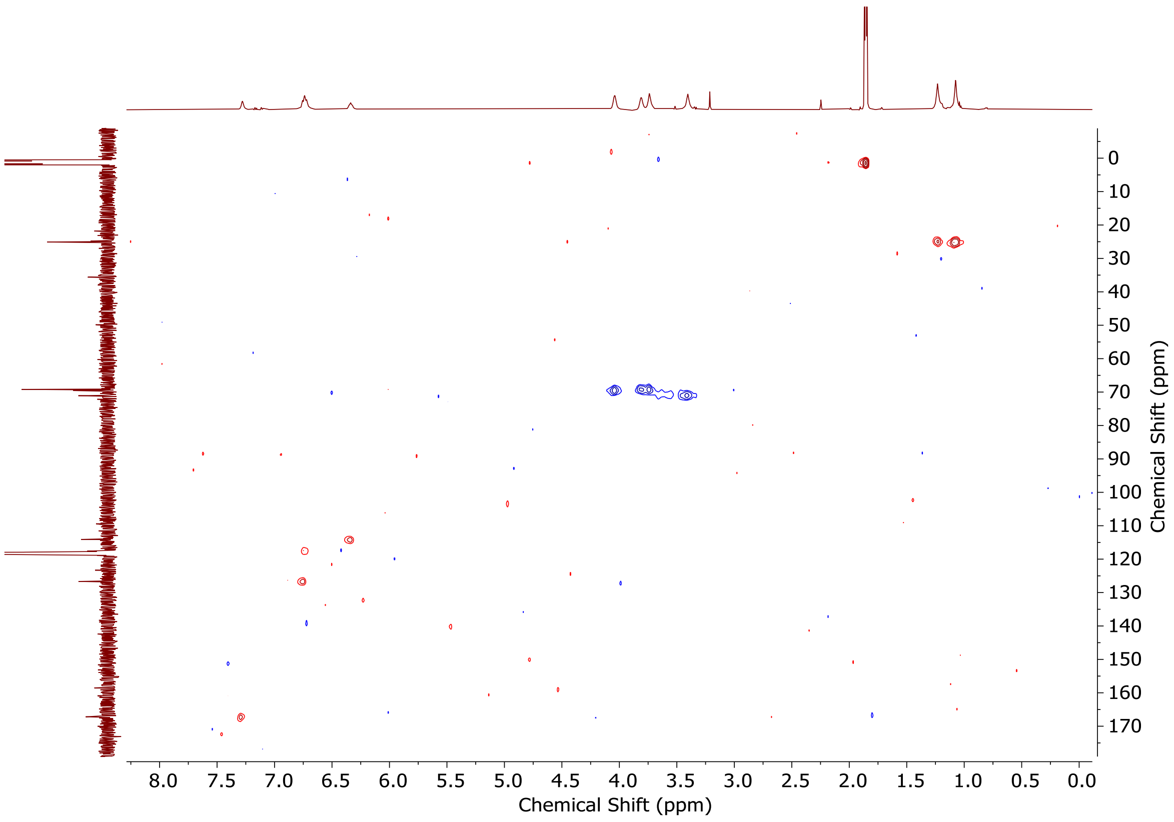

Figure S25. ^{1}H -^{13}C HSQC NMR spectrum of $L_4Co(III)Na(I)$

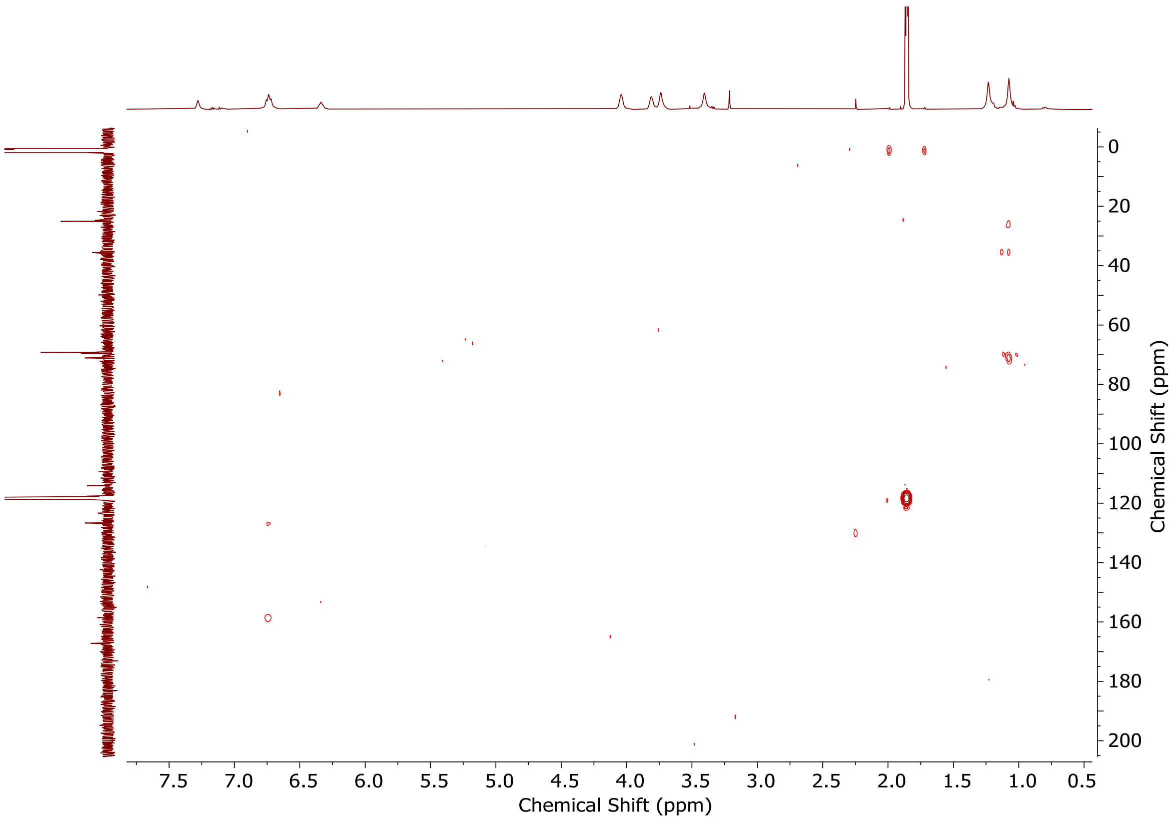

Figure S26. ^{1}H-^{13}C HMBC NMR spectrum of $L_4Co(III)Na(I)$

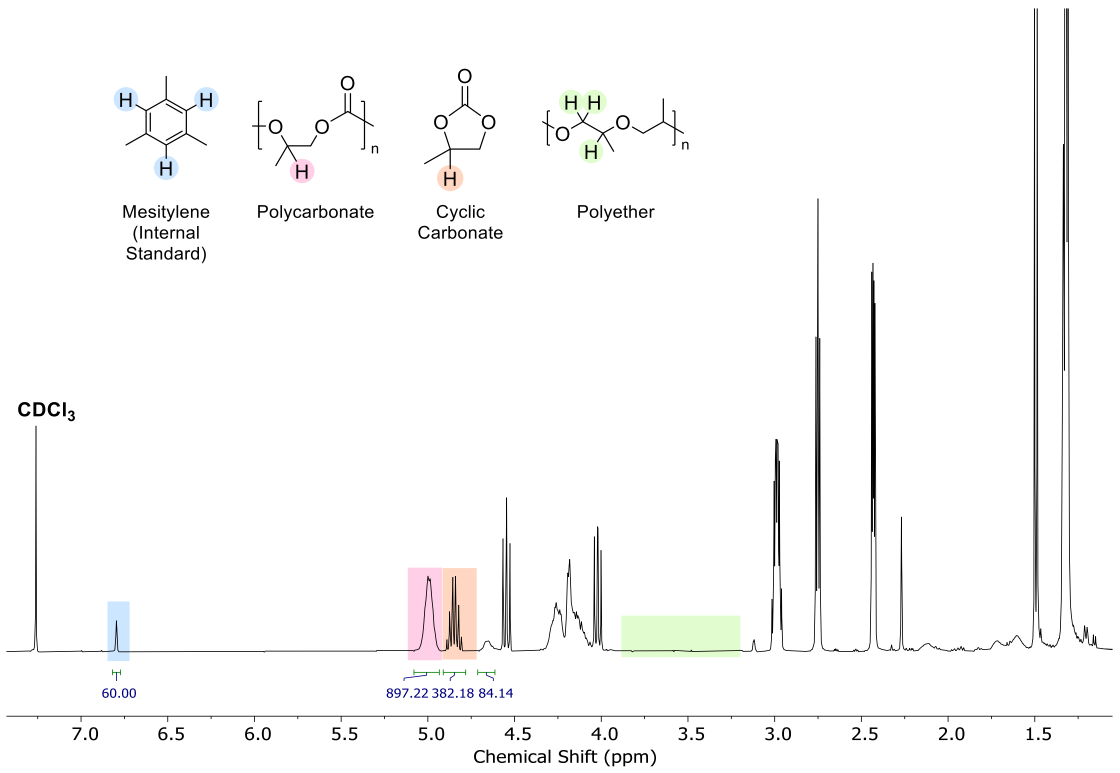

Figure S27. Representative ^{1}H NMR spectrum of an aliquot of a PO/CO_2 ROCOP polymerization, showing peaks corresponding to polycarbonate (PPC) and cyclic carbonate (PC) formation

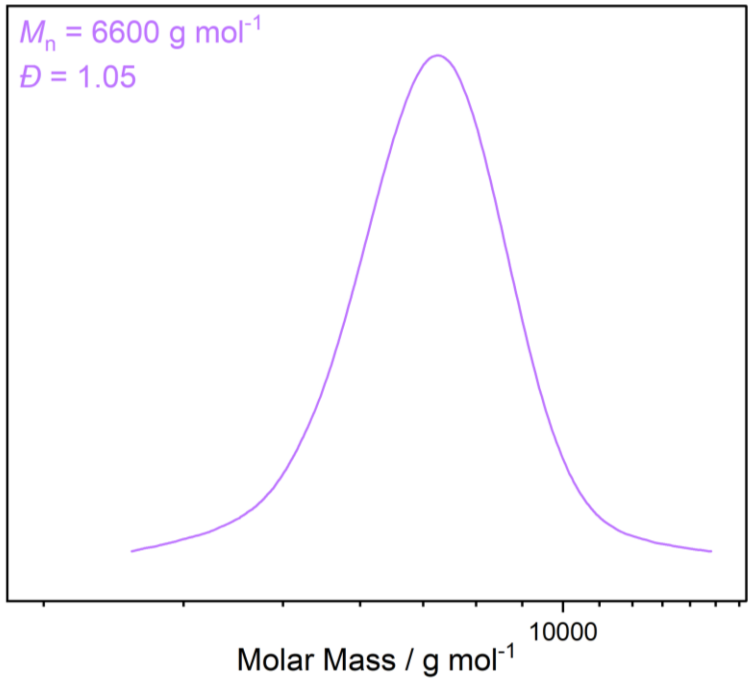

Figure S28. GPC trace of polycarbonate produced from the ROCOP of PO and CO_2 using L_1Co(III)Na(I) (50 °C, 20 bar CO_2)

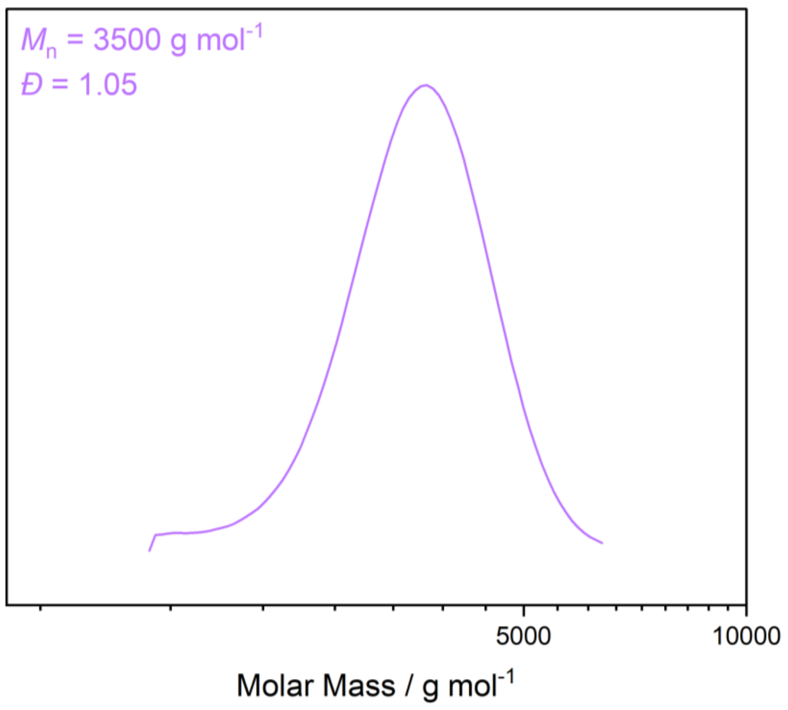

Figure S29. GPC trace of polycarbonate produced from the ROCOP of PO and CO_2 using $L_2Co(III)Na(I)$ (50 °C, 20 bar CO_2)

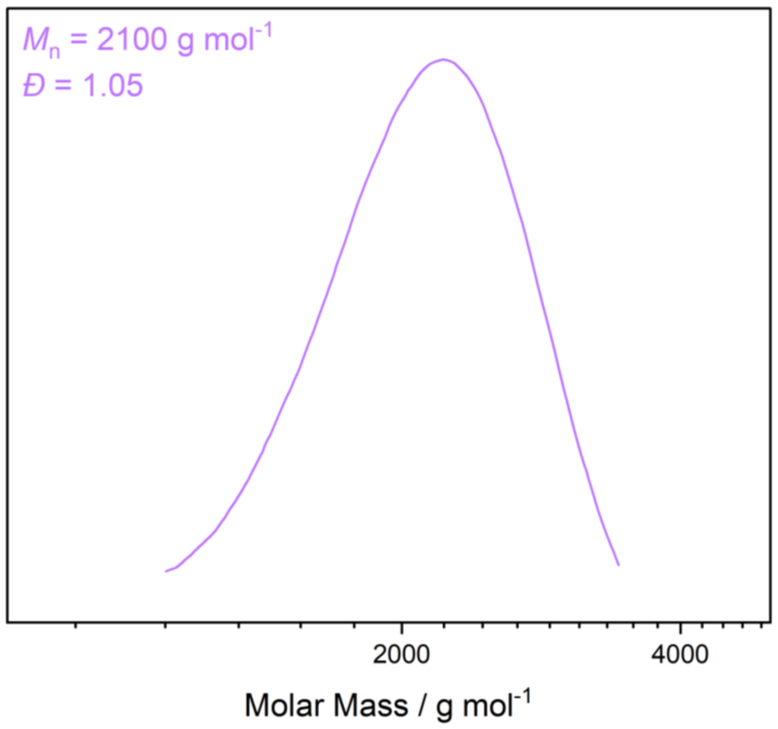

Figure S30. GPC trace of polycarbonate produced from the ROCOP of PO and CO_2 using $L_3Co(III)Na(I)$ (50 °C, 20 bar CO_2)

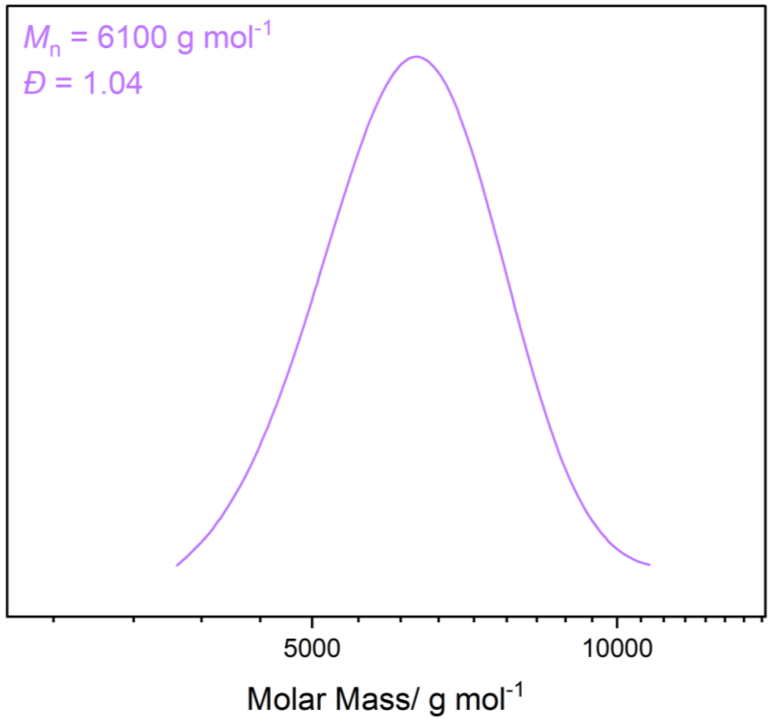

Figure S31. GPC trace of polycarbonate produced from the ROCOP of PO and CO_2 using L_1Co(III)Na(I) (70 °C, 20 bar CO_2)

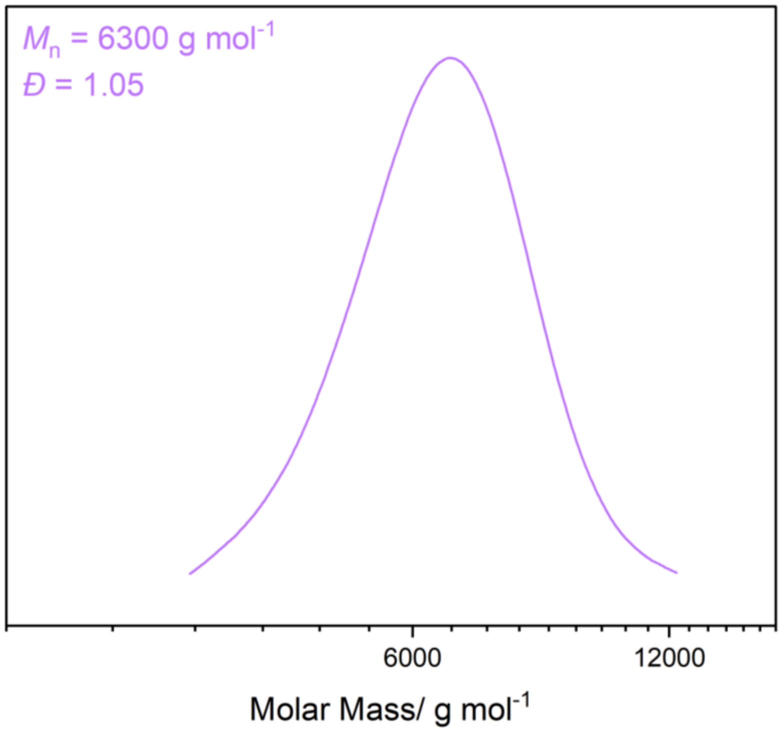

Figure S32. GPC trace of polycarbonate produced from the ROCOP of PO and CO_2 using L_2Co(III)Na(I) (70 °C, 20 bar CO_2)

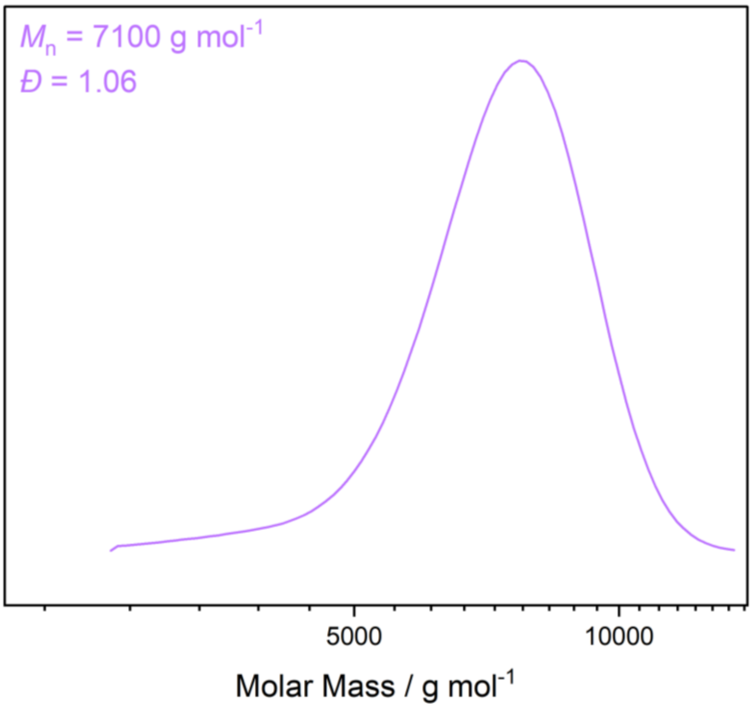

Figure S33. GPC trace of polycarbonate produced from the ROCOP of PO and CO_2 using $L_1Co(III)Na(I)$ (50 °C, 20 bar CO_2, unpurified PO)

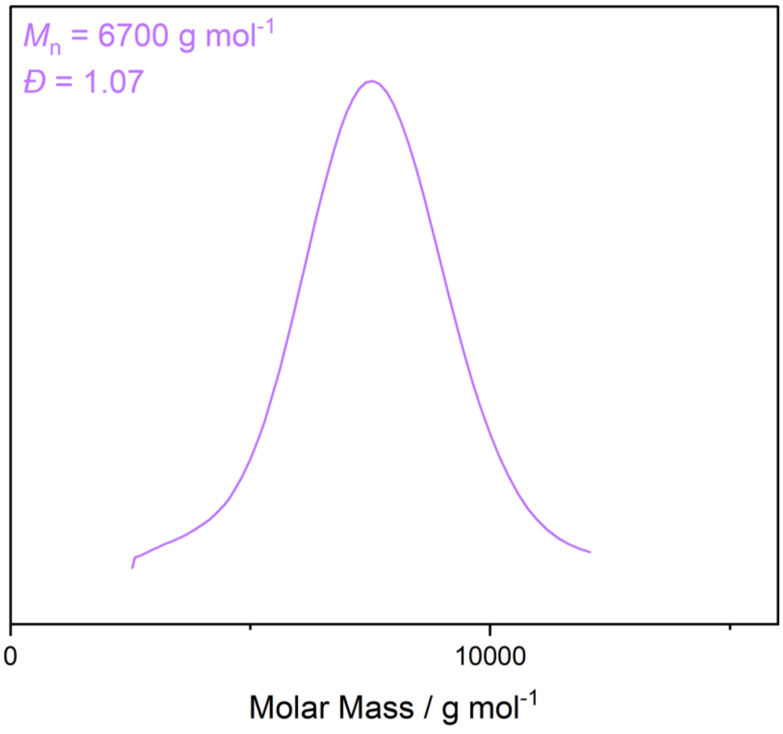

Figure S34. GPC trace of polycarbonate produced from the ROCOP of PO and CO_2 using $L_2Co(III)Na(I)$ (50 °C, 20 bar CO_2, unpurified PO)

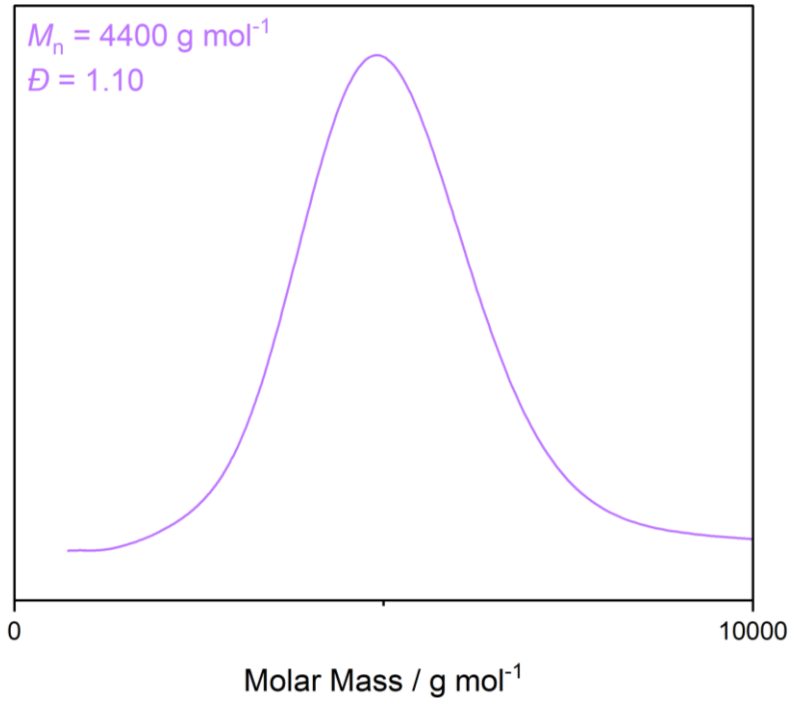

Figure S35. GPC trace of polycarbonate produced from the ROCOP of PO and CO_2 using $L_3Co(III)Na(I)$ (50 °C, 20 bar CO_2, unpurified PO)

Calculation of TOF_{PPC} from k_{obs}:

Table S1. Values for derivation of Equation S1

Conversion / %	TON	$[PO]_t$ / M	$\ln([PO]_t/[PO]_0)$
5	200	13.59	-0.0513
20	800	11.44	-0.2231

Table S2. Description of parameters used for the derivation of Equation S1

Parameters	Description
$[PO]_t$	Concentration of PO at time t
$[PO]_0$	Initial concentration of PO (i.e. at time = 0)
k_{obs}	Observed rate constant
T	Time
t_i	Initiation time
$TOF_{5\text{-}20\%}$	Turnover frequency between 5 and 20% conversion
$TON_{20\%}$	Turnover number at 20% conversion
$TON_{5\%}$	Turnover number at 5% conversion
$t_{20\%}$	Time at 20% conversion
$t_{5\%}$	Time at 5% conversion

Derivation of Equation S1:

$$\ln\frac{[\mathrm{PO}]_\mathrm{t}}{[\mathrm{PO}]_0} = -k_{\mathrm{obs}}t + t_i$$

$$\mathrm{TOF}_{5-20\%} = \frac{\mathrm{TON}_{20\%} - \mathrm{TON}_{5\%}}{t_{20\%} - t_{5\%}} = \frac{800 - 200}{t_{20\%} - t_{5\%}} = \frac{600}{t_{20\%} - t_{5\%}}$$

$$t = \frac{t_i - \ln\frac{[\mathrm{PO}]_\mathrm{t}}{[\mathrm{PO}]_0}}{k_{\mathrm{obs}}}$$

$$t_{5\%} = \frac{t_i + 0.0513}{k_{\mathrm{obs}}} \qquad t_{20\%} = \frac{t_i + 0.2231}{k_{\mathrm{obs}}}$$

$$t_{20\%} - t_{5\%} = \frac{t_i + 0.2231}{k_{\mathrm{obs}}} - \frac{(t_i + 0.0513)}{k_{\mathrm{obs}}} = \frac{0.17}{k_{\mathrm{obs}}}$$

$$\mathrm{TOF}_{5-20\%} = \frac{600 \times k_{\mathrm{obs}} \times 3600}{0.17} = k_{\mathrm{obs}} \times 1.271 \times 10^7\ \mathrm{h}^{-1} \qquad \text{(S1)}$$

Table S3. Data for PO/CO_2 ROCOP using L_nCo(III)Na(I) catalysts (n = 1 – 4)[a]

Catalyst	Overall TON [b]	CO_2 Sel. / % [c]	PPC Sel. / % [d]	TOF_{PPC} / h^{-1} [e]	k_{obs}/ 10^{-6} s^{-1} [f]	M_n [$Đ$] / g mol^{-1} [g]	M_{theo} / g mol^{-1} [h]
L_1Co(III)Na(I)	1577 ± 32	>99	97 ± 1	263 ± 20	20.65 ± 1.59	6600 [1.05]	7000
L_2Co(III)Na(I)	588 ± 21	>99	96 ± 1	76 ± 1	5.99 ± 0.03	3500 [1.05]	2700
L_3Co(III)Na(I)	568 ± 32	96	81 ± 1	49 ± 4	3.82 ± 0.33	2100 [1.05]	2300
L_4Co(III)Na(I)	28 ± 2	>99	0	0	0	0	0

[a]Conditions: catalyst (0.025 mol%, 3.6 mM), PO (5 mL, 14.3 M, neat), *trans*-1,2-cyclohexanediol (0.5 mol%, 71 mM), 20 bar CO_2, 50 °C. All reactions were conducted in duplicate and errors calculated using SE = $\sigma/\sqrt{n}$ (where σ = standard deviation and n = number of samples). [b]Overall turnover number (TON) = moles of PO consumed/ moles of catalyst, calculated from the relative integrals in the ^{1}H NMR spectrum of PPC (4.99 ppm, 1 H), PC (4.84 ppm, 1 H), and PPO (3.46-3.75 ppm, 3 H) using mesitylene as an internal standard (6.80 ppm). [c]CO_2 selectivity determined by the relative integrals in the ^{1}H NMR spectrum of PPC (4.99 ppm, 1 H) and PC (4.84 ppm, 1 H) compared with PPO (3.46-3.75 ppm, 3 H). [d] PPC selectivity determined by the relative integrals in the ^{1}H NMR spectrum of PPC (4.99 ppm, 1 H) against PC (4.84 ppm, 1 H). Reported at a common PO conversion (15 %) in all cases. [e]Turnover frequency to PPC (TOF_{PPC}) calculated from k_{obs} (Tables S1 and S2, Eqn S1). [f]k_{obs} determined from the linear region of the plot of ln($[PO]_t/[PO]_0$) vs. time. [g]Determined by GPC analysis, in THF, calibrated with narrow-M_n polystyrene standards; dispersity values in parentheses. [h]Theoretical molecular weights calculated by TON_{PPC} x M_{RU} / $n_{initiators}$ where TON_{PPC} is the turnover number to poly(propene carbonate), M_{RU} is the molar mass of the repeat unit, and $n_{initiators}$ is the number of initiators (includes acetate co-ligands on the catalyst and those added to the polymerization as cyclohexane diol).

Table S4. Data for PO/CO_2 ROCOP at 70 °C using L_nCo(III)Na(I) catalysts (n = 1 – 3)[a]

Catalyst	Overall TON [b]	CO_2 Sel. / % [c]	PPC Sel. / % [d]	TOF_{PPC} / h^{-1} [e]	k_{obs}/ 10^{-6} s^{-1} [f]	M_n [$Đ$] / g mol^{-1} [g]	M_{theo} / g mol^{-1} [h]
L_1Co(III)Na(I)	1503 ± 75	99	98 ± 1	1428 ± 94	112.34 ± 7.39	6100 [1.04]	6900
L_2Co(III)Na(I)	1534 ± 31	99	85 ± 1	275 ± 17	21.60 ± 1.34	6300 [1.05]	6000
L_3Co(III)Na(I)	283 ± 14	86	0	0	0	0	0

[a]Conditions: catalyst (0.025 mol%, 3.6 mM), PO (5 mL, 14.3 M, neat), *trans*-1,2-cyclohexanediol (0.5 mol%, 71 mM), 20 bar CO_2, 70 °C. All reactions were conducted in duplicate and errors calculated using SE = $\sigma/\sqrt{n}$ (where σ = standard deviation and n = number of samples). [b]Overall turnover number (TON) = moles of PO consumed/ moles of catalyst, calculated from the relative integrals in the ^{1}H NMR spectrum of PPC (4.99 ppm, 1 H), PC (4.84 ppm, 1 H), and PPO (3.46-3.75 ppm, 3 H) using mesitylene as an internal standard (6.80 ppm). [c]CO_2 selectivity determined by the relative integrals in the ^{1}H NMR spectrum of PPC (4.99 ppm, 1 H) and PC (4.84 ppm, 1 H) compared with PPO (3.46-3.75 ppm, 3 H). [d] PPC selectivity determined by the relative integrals in the ^{1}H NMR spectrum of PPC (4.99 ppm, 1 H) against PC (4.84 ppm, 1 H). Reported at a common PO conversion (15 %) in all cases. [e]Turnover frequency to PPC (TOF_{PPC}) calculated from k_{obs} (Tables S1 and S2, Eqn S1). [f]k_{obs} determined from the linear region of the plot of $\ln([PO]_t/[PO]_0)$ vs. time. [g]Determined by GPC analysis, in THF, calibrated with narrow-M_n polystyrene standards; dispersity values in parentheses. [h]Theoretical molecular weights calculated by TON_{PPC} x M_{RU} / $n_{initiators}$ where TON_{PPC} is the turnover number to poly(propene carbonate), M_{RU} is the molar mass of the repeat unit, and $n_{initiators}$ is the number of initiators (includes acetate co-ligands on the catalyst and those added to the polymerization as cyclohexane diol).

Table S5. Data for PO/CO_2 ROCOP with unpurified (non-distilled) PO using L_nCo(III)Na(I) catalysts (n = 1 – 3)[a]

Catalyst	Overall TON [b]	CO_2 Sel. / % [c]	PPC Sel. / % [d]	TOF_{PPC} / h^{-1} [e]	k_{obs}/ 10^{-6} s^{-1} [f]	M_n [$Đ$] / g mol^{-1} [g]	M_{theo} / g mol^{-1} [h]
L_1Co(III)Na(I)	1427 ± 71	>99	99 ± 1	253 ± 21	19.93 ± 1.67	7100 [1.06]	6600
L_2Co(III)Na(I)	1647 ± 132	>99	97 ± 1	102 ± 3	8.05 ± 0.23	6700 [1.07]	7400
L_3Co(III)Na(I)	1747 ± 140	99	89 ± 2	67 ± 1	5.24 ± 0.08	4400 [1.10]	7300

[a]Conditions: catalyst (0.025 mol%, 3.6 mM), unpurified PO (5 mL, 14.3 M, neat), *trans*-1,2-cyclohexanediol (0.5 mol%, 71 mM), 20 bar CO_2, 50 °C. All reactions were conducted in duplicate and errors calculated using SE = $\sigma/\sqrt{n}$ (where σ = standard deviation and n = number of samples). [b]Overall turnover number (TON) = moles of PO consumed/ moles of catalyst, calculated from the relative integrals in the ^{1}H NMR spectrum of PPC (4.99 ppm, 1 H), PC (4.84 ppm, 1 H), and PPO (3.46-3.75 ppm, 3 H) using mesitylene as an internal standard (6.80 ppm). [c]CO_2 selectivity determined by the relative integrals in the ^{1}H NMR spectrum of PPC (4.99 ppm, 1 H) and PC (4.84 ppm, 1 H) compared with PPO (3.46-3.75 ppm, 3 H). [d] PPC selectivity determined by the relative integrals in the ^{1}H NMR spectrum of PPC (4.99 ppm, 1 H) against PC (4.84 ppm, 1 H). Reported at a common PO conversion (15 %) in all cases. [e]Turnover frequency to PPC (TOF_{PPC}) calculated from k_{obs} (Tables S1 and S2, Eqn S1). [f]k_{obs} determined from the linear region of the plot of $\ln([PO]_t/[PO]_0)$ vs. time. [g]Determined by GPC analysis, in THF, calibrated with narrow-M_n polystyrene standards; dispersity values in parentheses. [h]Theoretical molecular weights calculated by TON_{PPC} x M_{RU} / $n_{initiators}$ where TON_{PPC} is the turnover number to poly(propene carbonate), M_{RU} is the molar mass of the repeat unit, and $n_{initiators}$ is the number of initiators (includes acetate co-ligands on the catalyst and those added to the polymerization as cyclohexane diol).

Single Crystal X-ray Diffractometry:

$L_1Co(III)Na(I)$ and $L_4Co(III)Na(I)$ are previously reported crystal structures.[1, 2] The CIF file for $L_4Co(III)Na(I)$ can be found at *Chem. Eur. J.* **2021**, *27* (47), 12224-12231 (CCDC: 2073149). The CIF file for $L_1Co(III)Na(I)$ can be found at *J. Am. Chem. Soc.* **2020**, *142* (45), 19150-19160 (CCDC: 2018623).

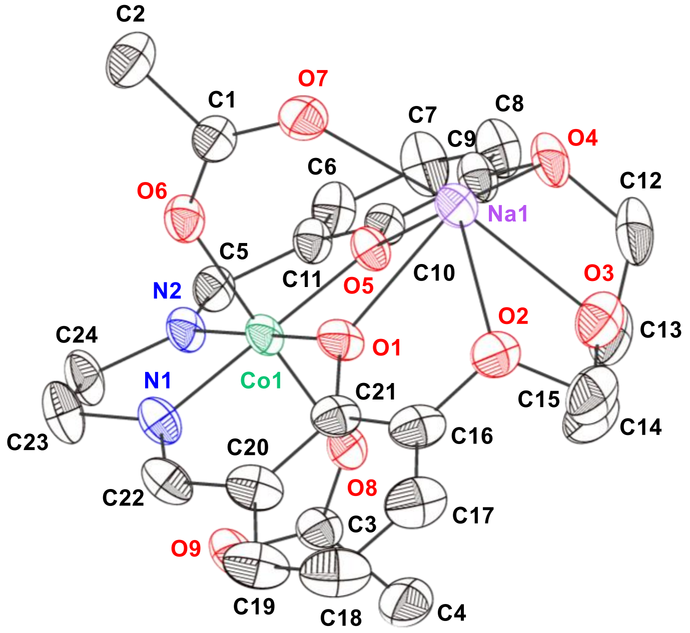

Figure S36. Crystal structure of $L_2Co(III)Na(I)$. Selected hydrogen atoms are omitted for clarity. Thermal ellipsoids are represented at 40% probability.

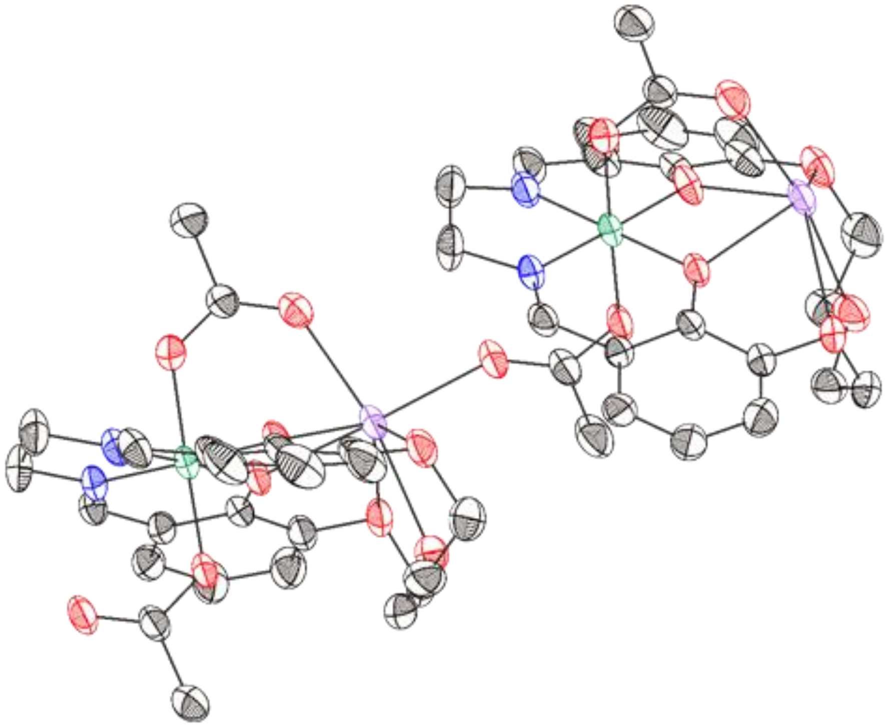

Figure S37. Polymeric crystal structure of $L_2Co(III)Na(I)$, showing an extra repeat unit. Selected hydrogen atoms are omitted for clarity. Thermal ellipsoids are represented at 40 % probability.

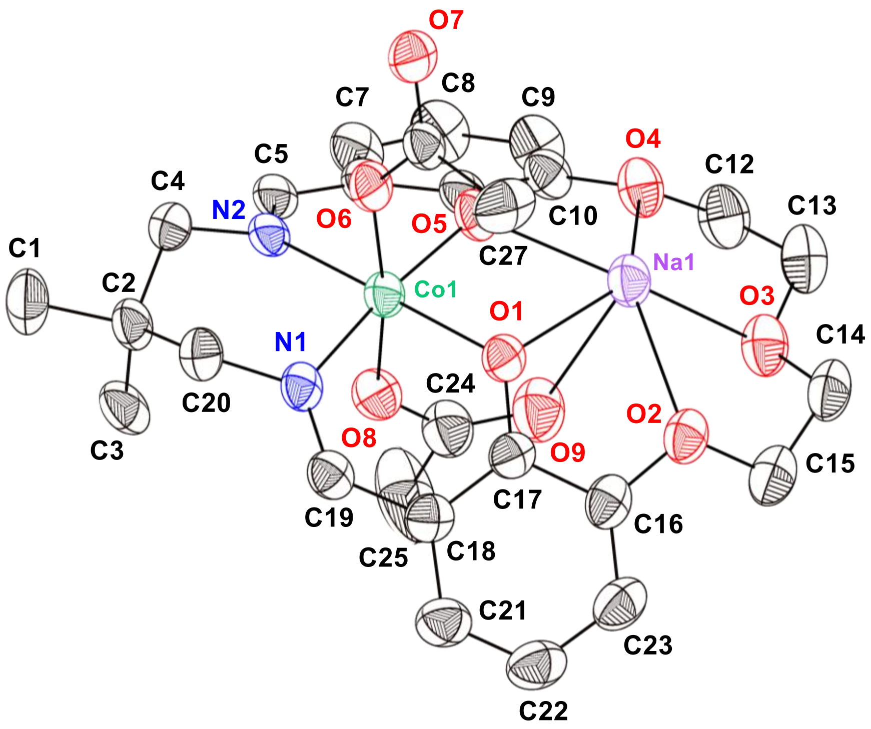

Figure S38. Crystal structure of $L_3Co(III)Na(I)$. Selected hydrogen atoms are omitted for clarity. Thermal ellipsoids are represented at 40% probability.

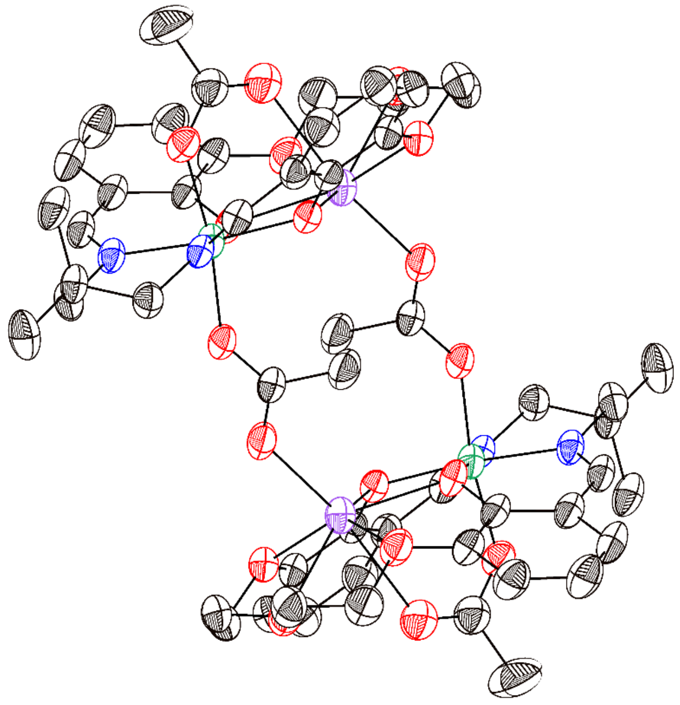

Figure S39. Dimeric structure of $L_3Co(III)Na(I)$. Selected hydrogen atoms are omitted for clarity. Thermal ellipsoids are represented at 40% probability.

Table S6. Crystallographic collection and refinement data for $L_2Co(III)Na(I)$

Empirical formula	$C_{33}H_{47}CoN_2NaO_9$
Formula weight	697.64
Temperature/K	150.15
Crystal system	monoclinic
Space group	$P2_1/c$
a/Å	10.9632(3)
b/Å	17.9328(5)
c/Å	16.3288(4)
α/°	90
β/°	108.259(3)
γ/°	90
Volume/Å^3	3048.62(15)
Z	4
ρ_{calc}g/cm^3	1.520
μ/mm^{-1}	5.067
F(000)	1476.0
Crystal size/mm^3	0.2 × 0.04 × 0.02
Radiation	CuKα (λ = 1.54184)
2Θ range for data collection/°	7.536 to 152.95
Index ranges	-13 ≤ h ≤ 10, -22 ≤ k ≤ 22, -20 ≤ l ≤ 20
Reflections collected	34977
Independent reflections	6352 [R_{int} = 0.0691, R_{sigma} = 0.0482]
Data/restraints/parameters	6352/0/336
Goodness-of-fit on F^2	1.042
Final R indexes [I>=2σ (I)]	R_1 = 0.0628, wR_2 = 0.1805
Final R indexes [all data]	R_1 = 0.0795, wR_2 = 0.1950
Largest diff. peak/hole / e Å^{-3}	0.45/-0.43

Table S7. Selected bond lengths for $L_2Co(III)Na(I)$

Co1	Na1^1	3.2788(14)	O3	C14	1.438(7)
Co1	O5	1.891(3)	N2	C5	1.279(5)
Co1	O6	1.930(3)	N2	C24	1.469(5)
Co1	O8	1.906(3)	N1	C22	1.275(6)
Co1	O1	1.888(3)	N1	C23	1.463(5)
Co1	N2	1.885(3)	C11	C6	1.426(5)
Co1	N1	1.882(3)	C11	C10	1.412(5)
Na1	O5^2	2.317(3)	C1	C2	1.522(6)
Na1	O1^2	2.327(3)	C21	C20	1.412(6)
Na1	O2^2	2.841(4)	C21	C16	1.424(6)
Na1	O9	2.292(3)	C6	C7	1.399(6)
Na1	O7^2	2.323(3)	C6	C5	1.444(5)
Na1	O4^2	2.903(4)	C7	C8	1.369(7)
Na1	O3^2	2.450(4)	C20	C22	1.445(7)

O5 C11	1.298(5)	C20 C19	1.417(7)
O6 C1	1.276(5)	C16 C17	1.376(7)
O8 C3	1.273(5)	C10 C9	1.378(7)
O1 C21	1.310(5)	C3 C4	1.509(7)
O2 C16	1.384(6)	C19 C18	1.364(8)
O2 C15	1.425(6)	C17 C18	1.396(9)
O9 C3	1.227(5)	C24 C23	1.511(7)
O7 C1	1.240(5)	C9 C8	1.401(7)
O4 C10	1.394(5)	C13 C12	1.485(9)
O4 C12	1.442(6)	C15 C14	1.503(8)
O3 C13	1.419(6)		

Table S8. Selected bond angles for L$_2$Co(III)Na(I)

O5 Co1 Na1^1	43.76(8)	C21 O1 Na1^1	133.1(2)
O5 Co1 O6	92.41(12)	C16 O2 Na1^1	111.7(2)
O5 Co1 O8	86.32(12)	C16 O2 C15	114.3(4)
O6 Co1 Na1^1	83.71(8)	C15 O2 Na1^1	101.0(3)
O8 Co1 Na1^1	94.87(8)	C3 O9 Na1	145.3(3)
O8 Co1 O6	178.54(11)	C1 O7 Na1^1	130.7(3)
O1 Co1 Na1^1	44.05(9)	C10 O4 Na1^1	108.5(2)
O1 Co1 O5	86.06(12)	C10 O4 C12	113.3(3)
O1 Co1 O6	92.60(12)	C12 O4 Na1^1	101.9(3)
O1 Co1 O8	86.59(12)	C13 O3 Na1^1	113.0(3)
N2 Co1 Na1^1	136.72(11)	C13 O3 C14	112.0(4)
N2 Co1 O5	94.92(13)	C14 O3 Na1^1	110.5(3)
N2 Co1 O6	86.65(13)	C5 N2 Co1	125.8(3)
N2 Co1 O8	94.18(13)	C5 N2 C24	118.6(3)
N2 Co1 O1	178.79(14)	C24 N2 Co1	115.6(3)
N1 Co1 Na1^1	135.95(10)	C22 N1 Co1	125.7(3)
N1 Co1 O5	179.68(14)	C22 N1 C23	120.6(4)
N1 Co1 O6	87.67(14)	C23 N1 Co1	113.7(3)
N1 Co1 O8	93.60(15)	O5 C11 C6	125.4(3)
N1 Co1 O1	93.63(14)	O5 C11 C10	118.5(3)
N1 Co1 N2	85.39(14)	C10 C11 C6	116.1(4)
O5^2 Na1 Co1^2	34.36(7)	O6 C1 C2	114.5(4)
O5^2 Na1 O1^2	67.45(10)	O7 C1 O6	127.8(4)
O5^2 Na1 O2^2	121.35(11)	O7 C1 C2	117.7(4)
O5^2 Na1 O7^2	79.31(11)	O1 C21 C20	124.8(4)
O5^2 Na1 O4^2	60.60(9)	O1 C21 C16	117.7(4)
O5^2 Na1 O3^2	91.12(11)	N2 C5 C6	126.0(3)
O1^2 Na1 Co1^2	34.34(7)	O1^2 Na1 O3^2	94.11(13)
O1^2 Na1 O2^2	60.80(10)	O2^2 Na1 Co1^2	94.00(7)
O1^2 Na1 O4^2	122.87(10)	O2^2 Na1 O4^2	133.79(11)

Table S9. Crystallographic collection and refinement data for $L_3Co(III)Na(I)$

Empirical formula	$C_{27}H_{32}CoN_2NaO_9$
Formula weight	610.46
Temperature/K	150.15
Crystal system	triclinic
Space group	P-1
a/Å	9.8713(9)
b/Å	12.3903(15)
c/Å	13.4423(14)
α/°	80.489(9)
β/°	86.341(8)
γ/°	81.355(9)
Volume/Å^3	1601.8(3)
Z	2
ρ_{calc}g/cm^3	1.266
μ/mm^{-1}	4.753
F(000)	636.0
Crystal size/mm^3	0.14 × 0.08 × 0.05
Radiation	CuKα (λ = 1.54184)
2Θ range for data collection/°	6.672 to 154.018
Index ranges	$-11 \le h \le 12, -15 \le k \le 15, -16 \le l \le 16$
Reflections collected	24216
Independent reflections	6597 [R_{int} = 0.0565, R_{sigma} = 0.0500]
Data/restraints/parameters	6597/0/365
Goodness-of-fit on F^2	1.017
Final R indexes [I>=2σ (I)]	R_1 = 0.0541, wR_2 = 0.1460
Final R indexes [all data]	R_1 = 0.0727, wR_2 = 0.1599
Largest diff. peak/hole / e Å^{-3}	0.55/-0.40

Table S10. Selected bond lengths for $L_3Co(III)Na(I)$

Co1	Na1	3.2780(13)	O9	C24	1.230(5)
Co1	O1	1.9252(19)	C5	C6	1.439(5)
Co1	O5	1.907(2)	C17	C16	1.421(4)
Co1	O6	1.946(2)	C17	C18	1.409(5)
Co1	N2	1.950(2)	C16	C23	1.371(5)
Co1	N1	1.960(3)	C11	C6	1.420(4)
Co1	O8	1.937(2)	C11	C10	1.422(5)
Na1	O1	2.292(3)	C26	C27	1.506(4)
Na1	O5	2.268(2)	C18	C19	1.444(5)
Na1	O2	2.382(3)	C18	C21	1.409(5)
Na1	O4	2.374(3)	C6	C7	1.404(5)
Na1	O3	2.386(3)	O6	C26	1.269(4)
Na1	O9	2.652(3)	O7	C26	1.236(4)
O6	C26	1.269(4)			

Table S11. Selected bond angles for $L_3Co(III)Na(I)$

O1	Co1	Na1	43.22(7)	O1	Na1	O4	136.79(9)
O1	Co1	O6	98.69(9)	O1	Na1	$O7^{1}$	118.30(10)
O1	Co1	N2	173.39(11)	O1	Na1	O3	132.40(10)
O1	Co1	N1	89.07(10)	O1	Na1	O9	73.15(9)
O1	Co1	O8	90.61(9)	O5	Na1	Co1	34.52(6)
O5	Co1	Na1	42.37(7)	O5	Na1	O1	69.57(8)
O5	Co1	O1	85.50(9)	O5	Na1	O2	134.87(10)
O5	Co1	O6	91.01(10)	O5	Na1	O4	69.98(9)
O5	Co1	N2	90.92(10)	O5	Na1	$O7^{1}$	114.67(9)
O5	Co1	N1	173.98(10)	O5	Na1	O3	132.76(10)
O5	Co1	O8	90.78(10)	O5	Na1	O9	72.44(9)
O6	Co1	Na1	98.74(7)	O2	Na1	Co1	101.84(7)
O6	Co1	N2	86.91(10)	O2	Na1	O3	70.39(10)
O6	Co1	N1	92.39(11)	O2	Na1	O9	79.72(10)
N2	Co1	Na1	132.67(8)	O4	Na1	Co1	102.89(7)
N2	Co1	N1	94.22(10)	O4	Na1	O2	139.40(10)
N1	Co1	Na1	132.02(7)	O4	Na1	O3	70.59(10)
O8	Co1	Na1	88.75(8)	O4	Na1	O9	80.84(10)
O8	Co1	O6	170.64(9)	$O7^{1}$	Na1	Co1	124.50(8)
O8	Co1	N2	83.87(10)	$O7^{1}$	Na1	O2	99.78(9)
O8	Co1	N1	86.66(11)	$O7^{1}$	Na1	O4	92.08(10)
O1	Na1	Co1	35.11(5)	$O7^{1}$	Na1	O3	91.62(10)
O1	Na1	O2	68.72(8)	$O7^{1}$	Na1	O9	167.71(11)
O1	Na1	O4	136.79(9)	O3	Na1	Co1	143.82(8)

$L_2Co(III)Na(I)$ Catalyst Loading Calculation:

The mass of the $L_2Co(III)Na(I)$ catalyst to add to the reaction was calculated using the molar mass 568.40 g mol^{-1}, with corresponding formula unit $C_{24}H_{26}CoN_2NaO_9$. This is based on the assumption that the ratio of metals (i.e Co and Na 'active sites' in the catalyst) is 1:1 in the solid phase, as shown by the single crystal XRD structure, and supported by elemental analysis. This is also consistent with the 1:1 stoichiometric ratio of $Co(OAc)_2$ and Na(OAc) starting materials added during synthesis.

Regardless of the specific speciation, the ratio of Co(III) to Na(I) metal active sites remains at 1:1 in solution. While the exact environment of these metal sites under the specific conditions of catalysis (in neat epoxide) is unclear (e.g., whether Na(I) is bound by one ligand or bridging between two ligand molecules), this ambiguity does not affect the catalyst loading calculation.

$L_2Co(III)Na(I)$ Speciation in Epoxide:

The speciation of $L_2Co(III)Na(I)$ in epoxide (PO) was investigated using NMR spectroscopy. To perform this analysis using non-deuterated PO, deuterated solvent (MeCN-d_3) was sealed in a capillary tube and placed inside a J Young NMR tube containing a saturated solution of $L_2Co(III)Na(I)$ in PO.

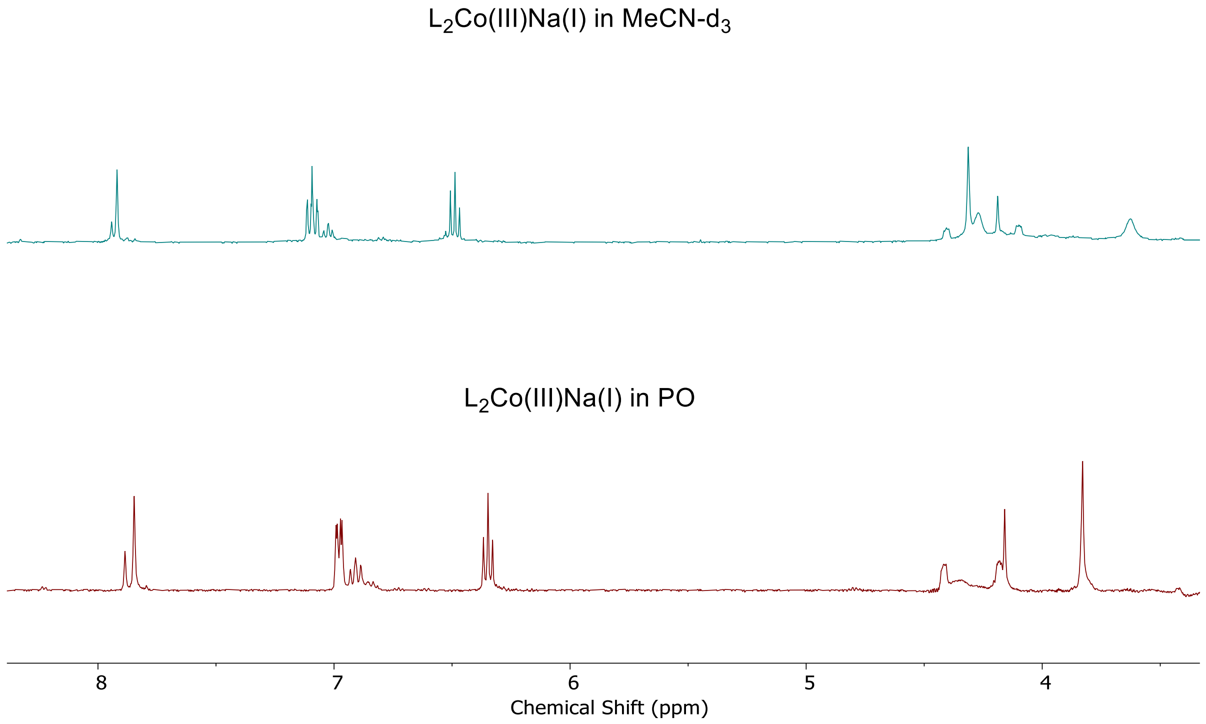

Figure S40. ^{1}H NMR spectrum of $L_2Co(III)Na(I)$ dissolved in MeCN-d_3 (top) and spectrum of $L_2Co(III)Na(I)$ dissolved in PO (bottom)

Comparing the top and bottom spectra, the overall number of peaks remains the same (Fig. S40). This indicates that there are two species present in PO, as in MeCN-d_3 (although their integration ratio appears to change slightly in the different solvent medium).

However, the solution of the catalyst in PO is saturated in these experiments, and this saturation is necessary such that clear signals from the complex are observed given the intense PO C-H signals. This means the catalyst is present at much higher concentration in this experiment than during catalysis. Furthermore, the experiment was run at room temperature, and without any chain transfer added. Therefore, the conclusions that can be drawn from this experiment about catalysis speciation under the reaction conditions are limited.

Buried Volume Analysis and Steric maps:

Buried volume around Co(III) and topographical steric maps were generated using the SambVca 2.1 online web tool, from XRD data (using an 'xyz' file with Co(III) positioned at the origin as an input file).[3-6]

Table S12. Parameters for buried volume calculation and generation of steric maps

Select the atoms coordinated to the center of the sphere	Co
Select the atoms for z axis definition (negative)	O atom on the acetate co-ligand
Select the atoms for xz-plane definition	N atom on the salen ligand
Select the atoms to be deleted	All solvent atoms and atoms belonging to the acetate co-ligands
Select the atomic radii	Bond radii scaled by 1.17
Sphere radius	5 Å
Mesh spacing for numerical integration	0.10 Å
Include H atoms in the calculations?	No

In most cases, default parameters were selected and identical parameters used for each catalyst. A larger sphere radius (5 Å instead of the default 3.5 Å) was chosen in order to incorporate all of the 2,2-dimethyl diamine.

The buried volume is independent of the orientation of the axes chosen (i.e, which acetate O is selected to be the z-direction). Steric maps are generated looking along the positive z-direction, selected as the face of the molecule of least steric hinderance based on the positioning of ligand atoms in the crystal structure.

Table S13. Buried volume around Co(III) for L_nCo(III)Na(I) catalysts (n = 1 – 4)

Catalyst	Total Buried Volume (V_{bur}) / %
L_1Co(III)Na(I)	51
L_2Co(III)Na(I)	55
L_3Co(III)Na(I)	56
L_4Co(III)Na(I)	57

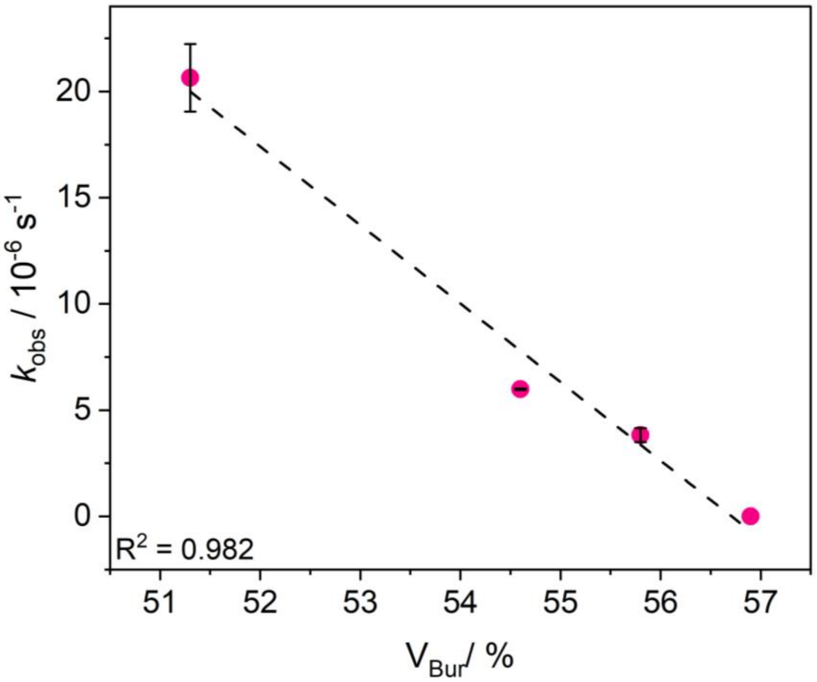

Figure S41. Plot of k_{obs} for $L_nCo(III)Na(I)$ (n = 1 – 4) versus buried volume (V_{Bur}) around Co(III)

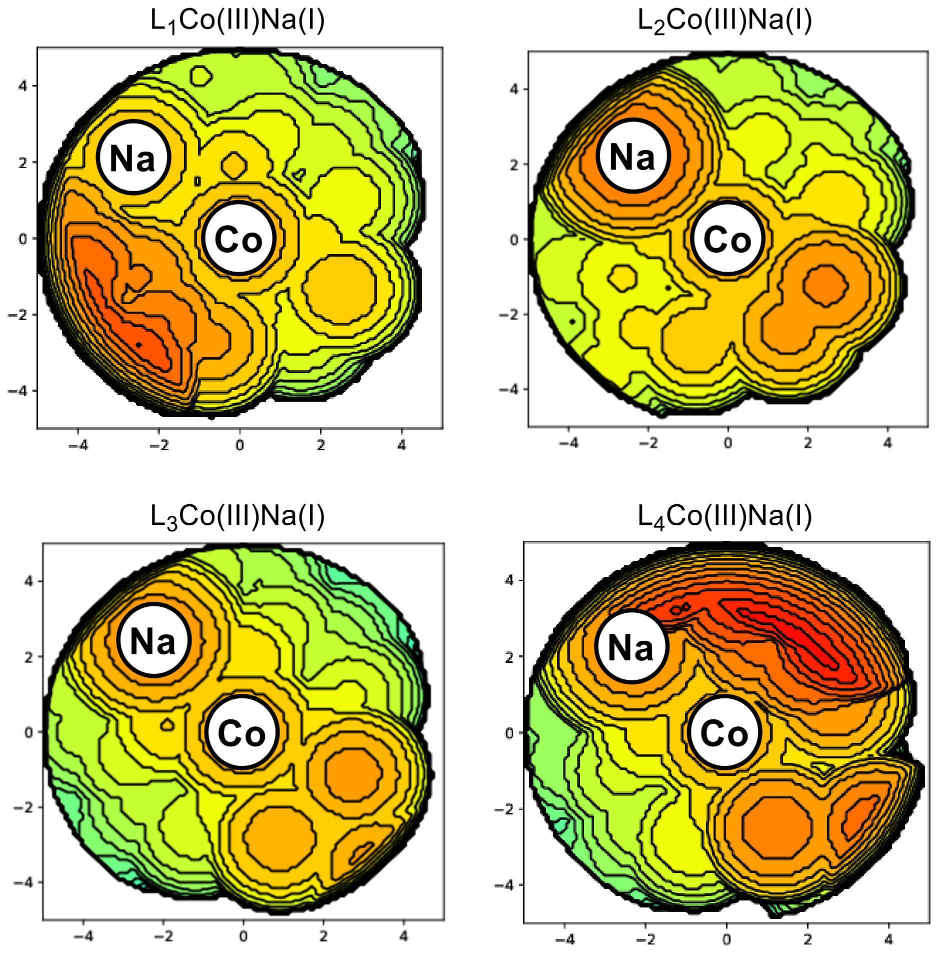

Figure S42. Topographical steric maps of $L_nCo(III)Na(I)$ (n = 1 – 4) viewed along the positive z-direction

Table S14. Data for comparison of L_1Co(III)Na(I) (**A**) against high-performance literature Co(III) PO/CO_2 ROCOP catalysts[a]

Catalyst	PPC Sel. / %	TOF_{PPC}/ h^{-1} [b]	Activity/ h^{-1} [c]
**A**	97	1428	238
**B**[1]	63	525	85
**C**[7]	97	1013	164
**D**[8]	>99	19000	859
**E**[9]	82	231	23

[a]Conditions: 70 °C, 20 bar CO_2, 0.025 mol% catalyst (A, B and C); 70-77 °C, 20 bar CO_2, 0.001 mol% catalyst (D); 50 °C, 20 bar CO_2 0.1 mol% catalyst (E).[b]TOF_{PPC} refers to moles of PPC produced per mole of catalyst per hour, calculated from overall TOF x PPC selectivity. [c]Activity refers to gram of PPC produced per gram of catalyst per hour, calculated from TOF_{PPC} x (M_{RU}/$M_{catalyst}$) where M_{RU} is the molar mass of the polycarbonate repeat unit (= 102 g mol^{-1} for PPC) and $M_{catalyst}$ is the molar mass of the catalyst.

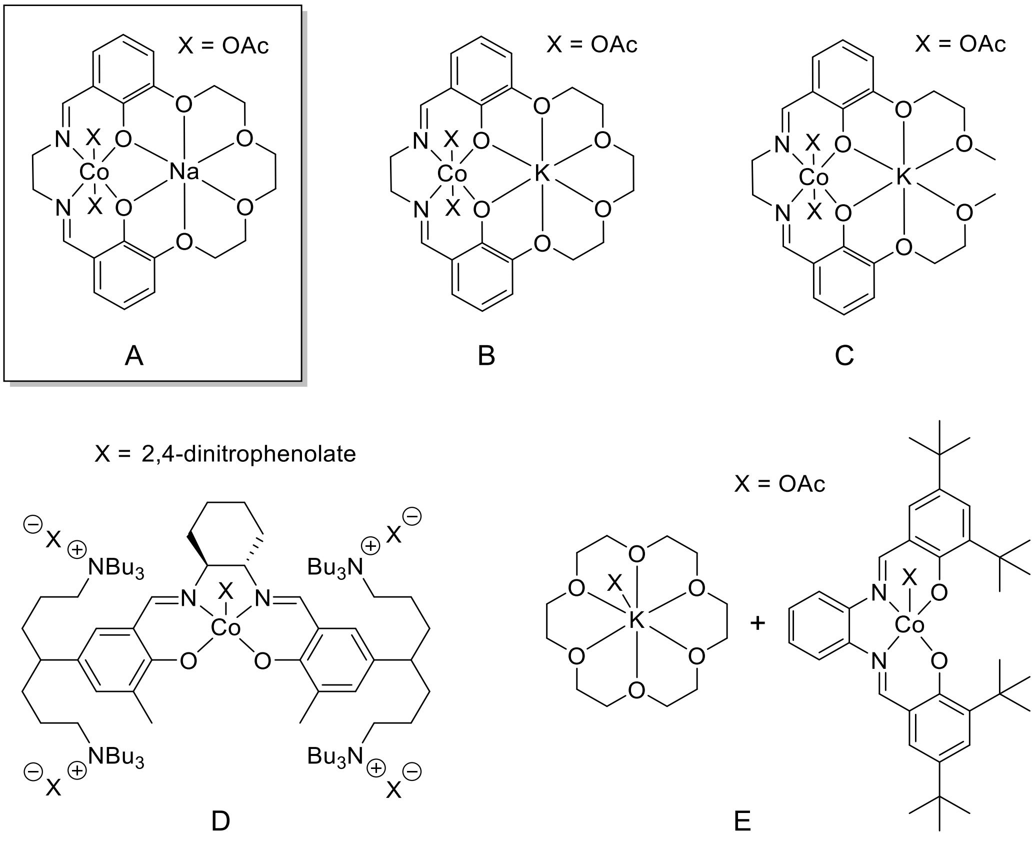

References:

(1) Deacy, A. C.; Moreby, E.; Phanopoulos, A.; Williams, C. K. Co(III)/Alkali-Metal(I) Heterodinuclear Catalysts for the Ring-Opening Copolymerization of CO_2 and Propylene Oxide. *J. Am. Chem. Soc.* **2020**, *142* (45), 19150-19160.

(2) Lindeboom, W.; Fraser, D. A. X.; Durr, C. B.; Williams, C. K. Heterodinuclear Zn(II), Mg(II) or Co(III) with Na(I) Catalysts for Carbon Dioxide and Cyclohexene Oxide Ring Opening Copolymerizations. *Chem. Eur. J.* **2021**, *27* (47), 12224-12231.

(3) Falivene, L.; Cao, Z.; Petta, A.; Serra, L.; Poater, A.; Oliva, R.; Scarano, V.; Cavallo, L. Towards the online computer-aided design of catalytic pockets. *Nat. Chem.* **2019**, *11* (10), 872-879.

(4) Falivene, L.; Credendino, R.; Poater, A.; Petta, A.; Serra, L.; Oliva, R.; Scarano, V.; Cavallo, L. SambVca 2. A Web Tool for Analyzing Catalytic Pockets with Topographic Steric Maps. *Organomet.* **2016**, *35* (13), 2286-2293.

(5) Poater, A.; Ragone, F.; Giudice, S.; Costabile, C.; Dorta, R.; Nolan, S. P.; Cavallo, L. Thermodynamics of N-Heterocyclic Carbene Dimerization: The Balance of Sterics and Electronics. *Organomet.* **2008**, *27* (12), 2679-2681.

(6) Poater, A.; Ragone, F.; Mariz, R.; Dorta, R.; Cavallo, L. Comparing the Enantioselective Power of Steric and Electrostatic Effects in Transition-Metal-Catalyzed Asymmetric Synthesis. *Chem. Eur. J.* **2010**, *16* (48), 14348-14353.

(7) Eisenhardt, K. H. S.; Fiorentini, F.; Lindeboom, W.; Williams, C. K. Quantifying CO_2 Insertion Equilibria for Low-Pressure Propene Oxide and Carbon Dioxide Ring Opening Copolymerization Catalysts. *J. Am. Chem. Soc.* **2024**, *146* (15), 10451-10464.

(8) S, S.; Min, J. K.; Seong, J. E.; Na, S. J.; Lee, B. Y. A Highly Active and Recyclable Catalytic System for CO_2/Propylene Oxide Copolymerization. *Angew. Chem. Int. Ed.* **2008**, *47* (38), 7306-7309.

(9) Fiorentini, F.; Eisenhardt, K. H. S.; Deacy, A. C.; Williams, C. K. Synergic Catalysis: the Importance of Intermetallic Separation in Co(III)K(I) Catalysts for Ring Opening Copolymerizations. *J. Am. Chem. Soc.* **2024**, *146* (33), 23517-23528.
